# Hybrid Cycloalkyl‐Alkyl Chain‐Based Symmetric/Asymmetric Acceptors with Optimized Crystal Packing and Interfacial Exciton Properties for Efficient Organic Solar Cells

**DOI:** 10.1002/advs.202206580

**Published:** 2023-01-02

**Authors:** Cong Xiao, Xunchang Wang, Tian Zhong, Ruixue Zhou, Xufan Zheng, Yirui Liu, Tianyu Hu, Yixuan Luo, Fengbo Sun, Biao Xiao, Zhitian Liu, Chunming Yang, Renqiang Yang

**Affiliations:** ^1^ Key Laboratory of Optoelectronic Chemical Materials and Devices (Ministry of Education) School of Optoelectronic Materials and Technology Jianghan University Wuhan 430056 China; ^2^ State Key Laboratory of Fine Blasting Jianghan University Wuhan 430056 China; ^3^ Hubei Engineering Technology Research Center of Optoelectronic and New Energy Materials Wuhan Institute of Technology Wuhan 430205 China; ^4^ Shanghai Synchrotron Radiation Facility Shanghai Advanced Research Institute Chinese Academy of Sciences Shanghai 201204 China

**Keywords:** asymmetric small molecule acceptors, crystal packing, organic solar cells, side‐chain engineering

## Abstract

Hybrid cycloalkyl‐alkyl side chains are considered a unique composite side‐chain system for the construction of novel organic semiconductor materials. However, there is a lack of fundamental understanding of the variations in the single‐crystal structures as well as the optoelectronic and energetic properties generated by the introduction of hybrid side chains in electron acceptors. Herein, symmetric/asymmetric acceptors (Y‐C10ch and A‐C10ch) bearing bilateral and unilateral 10‐cyclohexyldecyl are designed, synthesized, and compared with the symmetric acceptor 2,2′‐((2Z,2′Z)‐((12,13‐bis(2‐butyloctyl)‐3,9 bis(ethylhexyl)‐12,13‐dihydro‐[1,2,5]thiadiazolo[3,4‐e]thieno[2″,3″′:4′,5′]thieno[2′,3′:4,5] pyrrolo[3,2‐g]thieno[2′,3′:4,5]thieno[3,2‐b]indole‐2,10‐ diyl)bis(methanylylidene))bis(5,6‐difluoro‐3‐oxo‐2,3‐dihydro‐1H‐indene‐2,1‐diylidene))dimalononitrile (L8‐BO). The stepwise introduction of 10‐cyclohexyldecyl side chains decreases the optical bandgap, deepens the energy level, and enables the acceptor molecules to pack closely in a regular manner. Crystallographic analysis demonstrates that the 10‐cyclohexyldecyl chain endows the acceptor with a more planar skeleton and enforces more compact 3D network packing, resulting in an active layer with higher domain purity. Moreover, the 10‐cyclohexyldecyl chain affects the donor/acceptor interfacial energetics and accelerates exciton dissociation, enabling a power conversion efficiency (PCE) of >18% in the 2,2′‐((2Z,2′Z)‐((12,13‐bis(2‐ethylhexyl)‐3,9‐diundecyl12,13‐dihydro‐[1,2,5]thiadiazolo[3,4‐e]thieno[2″,3″′:4′,5′]thieno[2′,3′:4,5]pyrrolo[3,2‐g]thieno[2′,3′:4,5]thieno[3,2‐b]indole‐2,10‐diyl)bis(methanylylidene))bis(5,6‐difluoro‐3‐oxo‐2,3‐dihydro‐1H‐indene‐2,1‐diylidene))dimalononitrile (Y6) (PM6):A‐C10ch‐based organic solar cells (OSCs). Importantly, the incorporation of Y‐C10ch as the third component of the PM6:L8‐BO blend results in a higher PCE of 19.1%. The superior molecular packing behavior of the 10‐cyclohexyldecyl side chain is highlighted here for the fabrication of high‐performance OSCs.

## Graphical abstract

Here, a unique and effective hybrid side chain strategy is adopted for synthesizing symmetric/asymmetric small molecular acceptors and enables organic photovoltaic to exhibit a high efficiency of 19.1%. It endows the acceptors with planar skeleton and compact 3D network packing, contributing to optimal donor/acceptor interfacial energetics and accelerated exciton dissociation.

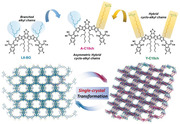

## 1 Introduction

Organic solar cells (OSCs) are a clean energy technology that has aroused wide interest because of their specific advantages, such as solution processability, selective absorption with semitransparency, and potential for flexible and large‐scale devices.^[^ ^1^, ^2^, ^3^, ^4^, ^5^, ^6^, ^7^, ^8^ ^]^ Great progress has been made in OSCs owing to comprehensive efforts in novel molecular design,^[^ ^9^, ^10^, ^11^, ^12^, ^13^, ^14^, ^15^, ^16^, ^17^ ^]^ precise morphology optimization, ^[^ ^18^, ^19^, ^20^, ^21^, ^22^, ^23^, ^24^, ^25^, ^26^ ^]^ and diverse device architecture.^[^ ^27^, ^28^, ^29^, ^30^, ^31^, ^32^ ^]^ Along with the rapid development of A–D–A‐type small‐molecule acceptors (SMAs), the understanding of the photophysical processes of converting light‐to‐electricity has advanced greatly, such as the improved utilization of near‐infrared photons and control of the interfacial energy offset.^[^ ^33^, ^34^, ^35^, ^36^, ^37^, ^38^, ^39^, ^40^ ^]^ Furthermore, the power conversion efficiency (PCE) has reached 19% in single‐junction OSCs.^[^ ^41^, ^42^, ^43^ ^]^ To further expedite the commercialization of OSCs, SMAs with low energy loss and high stability should be developed and the intermolecular packing of SMAs and interfacial exciton properties of active layers should be fundamentally understood.

Modularizing the synthesis of A–D–A‐type SMAs is a frequently used strategy that is based on the three fundamental design approaches of the central core electron‐donating unit (D),^[^ ^44^, ^45^, ^46^, ^47^ ^]^ end‐capped electron‐withdrawing unit (A),^[^ ^48^, ^49^, ^50^, ^51^ ^]^ and solubilizing side chains. Among them, the modification of the side chains, including the variation of size, topology (linear or branched), branching points, symmetry breaking, and dimension are the most commonly used approaches for precisely modulating the solubility, molecular crystallization, and packing behavior of SMAs.^[^ ^52^, ^53^, ^54^, ^55^, ^56^ ^]^ For example, Yan et al. demonstrated that different positions of alkyl‐chain‐branching can alter the molecular packing of SMAs and optimize the phase separation and exciton dissociation.^[^ ^52^ ^]^ Moreover, by replacing the straight *n*‐undecyl chain on Y6 with 2‐butyloctyl, the molecular packing behavior can be changed completely and the structural order and charge transport in thin films can be improved, delivering significantly improved open‐circuit voltage (*V*_oc_) and fill factor (FF).^[^ ^35^ ^]^ Huang et al. realized a PCE of 18.36% by designing the small molecule acceptor EH‐HD‐4F by exploiting asymmetric alkyl side chains and optimizing the miscibility and crystallinity in the bulk heterojunction layers.^[^ ^56^ ^]^ These encouraging results further inspire researchers to innovate side chains to fundamentally understand the structure–property relationships and maximize the potentials of SMAs.

In contrast to cyclic or alkyl chains, hybrid cycloalkyl‐alkyl chains are considered a unique composite side‐chain system in the construction of novel organic semiconductor materials,^[^ ^57^, ^58^ ^]^ with the potential to influence the planarity, molecular packing, and optoelectronic properties. The steric effect produced by combining the flexible alkyl unit and rigid cyclic unit can optimize the crystallization and intermolecular interactions and further influence the blend film morphology and photophysical properties. However, the fundamental properties of hybrid cycloalkyl‐alkyl chains are poorly understood, including their molecular conformations, single‐crystal intermolecular packing in SMAs, energetics, and optoelectronic properties. This knowledge is crucial for the development of fascinating Y6‐series SMAs and promotion of new material systems. In this work, the influence of SMAs with symmetric or asymmetric 10‐cyclohexyldecyl chains on the device performance was investigated from the perspectives of molecular packing in the solid state, photophysical properties, single‐crystal intermolecular packing of SMAs, morphology of neat and blend films, charge dynamic behaviors, etc.

Two new explored SMAs with symmetric and asymmetric hybrid cycloalkyl‐alkyl chains (Y‐C10ch and A‐C10ch, respectively) were compared in detail with the state‐of‐the‐art SMA 2,2′‐((2Z,2′Z)‐((12,13‐bis(2‐butyloctyl)‐3,9 bis(ethylhexyl)‐12,13‐dihydro‐[1,2,5]thiadiazolo[3,4‐e]thieno[2″,3″′:4′,5′]thieno[2′,3′:4,5] pyrrolo[3,2‐g]thieno[2′,3′:4,5]thieno[3,2‐b]indole‐2,10‐ diyl)bis(methanylylidene))bis(5,6‐difluoro‐3‐oxo‐2,3‐dihydro‐1H‐indene‐2,1‐diylidene))dimalononitrile (L8‐BO). Compared with L8‐BO, the variations to the side chain in the thiophene beta position of A‐C10ch and Y‐C10ch cause a narrower optical bandgap and slightly deeper energy level, which has the potential to balance the device parameters. The precise molecular packing was examined in single‐crystals, indicating that the 10‐cyclohexyldecyl chain endows the acceptor with a more planar skeleton and enforces more compact 3D network packing than L8‐BO, with the resulting active layer possessing higher domain purity. Furthermore, the 10‐cyclohexyldecyl chains can subtly influence the donor/acceptor interfacial energetics, thus accelerating exciton dissociation and suppressing energetic disorder. Together, these factors contribute to the remarkable device efficiencies of blends of A‐C10ch and Y‐C10ch with 2,2′‐((2Z,2′Z)‐((12,13‐bis(2‐ethylhexyl)‐3,9‐diundecyl12,13‐dihydro‐[1,2,5]thiadiazolo[3,4‐e]thieno[2″,3″′:4′,5′]thieno[2′,3′:4,5]pyrrolo[3,2‐g]thieno[2′,3′:4,5]thieno[3,2‐b]indole‐2,10‐diyl)bis(methanylylidene))bis(5,6‐difluoro‐3‐oxo‐2,3‐dihydro‐1H‐indene‐2,1‐diylidene))dimalononitrile (Y6) (PM6) as the donor polymer to afford maximum PCEs of 18.4% and 17.6%, respectively. More importantly, the application of Y‐C10ch as a third component in the PM6:L8‐BO blend resulted in a PCE of 19.1%, demonstrating great potential for high‐performance OSCs. Overall, our research demonstrates that hybrid cycloalkyl‐alkyl chains are not only a feasible approach for improving the intermolecular crystal packing of SMAs, readjusting the interfacial exciton properties and thus boosting OSC efficiency but also hold great potential for the development of novel materials in the field of organic optoelectronics.

## 2 Results and Discussion

### 2.1 Synthesis and Characterization

**Scheme 1:**
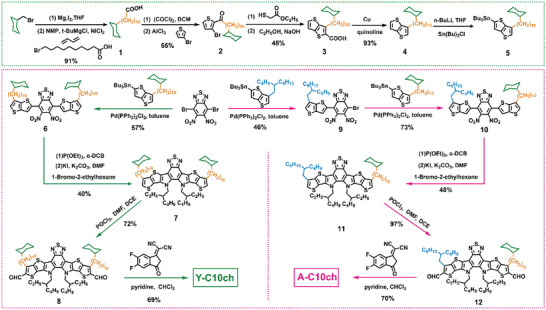
The synthetic routes of cycloalkyl‐alky side units and symmetric/asymmetric acceptors (Y‐C10ch and A‐C10ch).

The synthetic routes of cycloalkyl‐alky side units and symmetric/asymmetric acceptors (Y‐C10ch and A‐C10ch) are depicted in the **Scheme** **1**. The key synthetic procedure is to integrate and graft the cycloalkyl‐alky into the conjugated molecular backbone. The crosscoupling of bromoalkanoic acid and cyclic alkyl Grignard reagent catalyzed by nickel–butadiene was used to achieve the grafting of cyclohexyl decyl unit of compound 1 with a yield of 92%. Compound 2 was prepared by Friedel‐Crafts acylation with tribromothiophene in 55% yield. Then, ethyl thioglycolate was used to perform cyclization and hydrolysis reactions with compound 2, to give 3‐alkylthieno[3,2‐b]thiophene‐2‐carboxylic acid, which were further subjected to decarboxylation reaction with copper to give intermediate 4 in 93% yield. The symmetric/asymmetric compounds **6** and **10** were obtained by a Pd(PPh_3_)_2_Cl_2_‐catalyzed Stille coupling reaction using two organotin compounds. The coupling resultants **6** and **10** were separately subjected to an intramolecular Cadogan cyclization with a triethyl phosphite and followed by N‐alkylation with 2‐ethylhexyl as side chains to get symmetric or asymmetric central core **7** and **11**. Subsequently, the dialdehyde **8** and **12** were prepared using the Vilsmeier–Haack reaction, which were further reacted with terminal withdrawing unit 2‐(5,6‐difluoro3‐oxo‐2,3‐dihydro‐1H‐inden‐1‐ylidene) malononitrile (IC‐2F) in Knoevenagel condensation reactions to afford Y‐C10ch and A‐C10ch. The synthetic details and characterization of the intermediates and target materials (Y‐C10ch and A‐C10ch), including the ^1^H NMR and ^13^C NMR spectra, are outlined in Scheme S1 and Figures S1–S25 in the Supporting Information. Both the Y‐C10ch and A‐C10ch showed excellent solubility in common organic solvents such as dichloromethane, chloroform and chlorobenzene. From differential scanning calorimetry analysis (Figure S26, Supporting Information), it can be observed that L8‐BO, A‐C10ch, and Y‐C10ch exhibited exothermal peaks at 319.3, 293.3, and 290.4 °C, respectively. The different melting enthalpy (Δ*H*_m_) of L8‐BO (41.98 J g^−1^), A‐C10ch (29.12 J g^−1^), and Y‐C10ch (29.93 J g^−1^) implies that cyclohexyl decyl side chain modification could significantly control molecular aggregation properties.^[^ ^59^ ^]^

**Figure 1:**
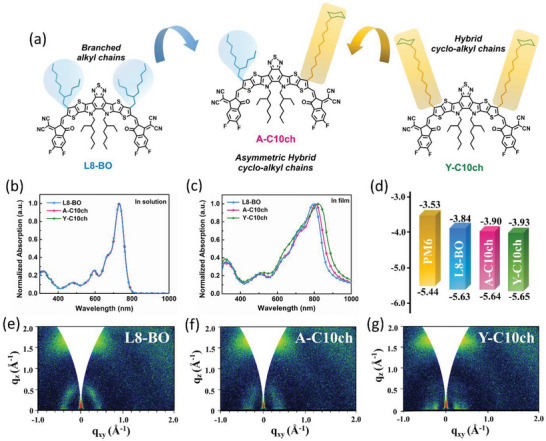
Structures and physical properties of the SMAs. a) Molecular structures of L8‐BO, A‐C10ch, and Y‐C10ch. b,c) Normalized UV–vis absorption spectra of the SMAs in chloroform solutions and in thin films. d) Energy level diagrams of PM6 and the SMAs in neat films. e–g) 2D‐GIWAXS patterns of the neat L8‐BO, A‐C10ch, and Y‐C10ch films.

The ultraviolet‐visible (UV–vis) light absorption of L8‐BO, A‐C10ch, and Y‐C10ch in solutions is displayed in **Figure** **1**b, with the data summarized in **Table** **1**. In dilute chloroform solution, the shapes of the normalized absorption curves were nearly identical for the three SMAs, with the maximum peaks located at 733 nm and comparable extinction coefficients. This may be due to the negligible contribution of the branched and cyclic alkyl chains to the conjugated or inductive effects.^[^ ^55^ ^]^ However, obvious redshifted absorption was observed from L8‐BO to A‐C10ch and Y‐C10ch in film (Figure 1c), corresponding to the stepwise narrowing of the optical bandgap (*E*_g_), indicating a greater extent of intermolecular packing and aggregation behavior in film brought about by the 10‐cyclohexyldecyl chains. Subsequently, we studied the effect of the 10‐cyclohexyldecyl chains on the molecular energy levels using cyclic voltammetry (CV) (Figure S27, Supporting Information). As presented in Figure 1d, both the highest occupied molecular orbital (HOMO) and lowest unoccupied molecular orbital (LUMO) energy levels decreased as the branched alkyl chains were replaced with 10‐cyclohexyldecyl chains. Compared with L8‐BO, the narrower electrochemical bandgaps of A‐C10ch and Y‐C10ch were consistent with the variations in their *E*_g_, which were further examined using ultraviolet photoelectron spectroscopy (Figure S28, Supporting Information).

**Table 1:** Photophysical and electrochemical properties of SMAs

Acceptors	*λ*_max_ [nm]		*λ*_onset_ [nm]	*ε*_sol_ [M^−1^ cm^−1^]	*E*_g_^opt^ [eV]	*E*_HOMO_^CV^ [eV]	*E*_LUMO_^CV^ [eV]
	In solution	Film	Film				
L8‐BO	733	800	880	2.08 × 10^5^	1.41	−5.63	−3.84
A‐C10ch	733	812	901	2.10 × 10^5^	1.38	−5.64	−3.90
Y‐C10ch	733	829	917	2.12 × 10^5^	1.35	−5.65	−3.93

Side‐chain engineering usually has a great influence on the aggregation properties of organic photovoltaic materials. Hence, 2D grazing‐incidence wide‐angle X‐ray scattering (GIWAXS) was conducted to provide insights into the effects of the 10‐cyclohexyldecyl chains on the crystallization and molecular packing. As shown in Figure 1e–g, all three SMAs exhibited well‐defined backbone peaks in the in‐plane (IP) direction and prominent (010) diffraction peaks in the out‐of‐plane (OOP) direction. The GIWAXS pattern analysis of the (h00) peak demonstrated that both A‐C10ch and Y‐C10ch showed a bimodal distribution, whereas L8‐BO showed a unimodal distribution. Moreover, the aggregation property was improved by substituting the branched alkyl chains with 10‐cyclohexyldecyl chains, as verified by the reduced *π*–*π* stacking distance, increased relative degree of crystallization values,^[^ ^60^ ^]^ and enlarged coherence length of the (010) *π*–*π* stacking (Figures S29–S30 and Table S1, Supporting Information). These variations may originate from the effect of the 10‐cyclohexyldecyl chains, in which the linear alkyl component may regulate the steric hindrance effect and the cyclic alkyl component may facilitate intermolecular interactions of the adjacent molecular chains, resulting in tight molecular packing, also observed in the single‐crystal structures (see below).

### 2.2 Molecular Packing in Single‐Crystals

**Figure 2:**
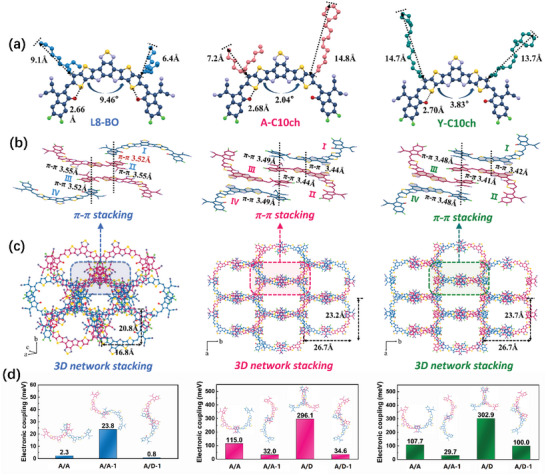
a) Monomolecular crystallographic structures of L8‐BO, A‐C10ch, and Y‐C10ch. b) Single‐crystal and *π*–*π* interactions for different dimers and c) 3D interpenetrating network packing of L8‐BO, A‐C10ch, and Y‐C10ch. d) Electronic coupling calculated values for different dimers of L8‐BO, A‐C10ch, and Y‐C10ch.

Single‐crystal X‐ray diffraction was used to investigate the effect of substituting the branched chains with 10‐cyclohexyldecyl chains on the single‐molecule geometry and intermolecular packing. The three molecules were crystallized with a 3D interpenetrating network structure in the monoclinic crystal system. Unlike the single‐crystal of L8‐BO with a C 1 2/c 1 space group, both the Y‐C10ch and A‐C10ch single‐crystals exhibited a similar C c space group (Table S2, Supporting Information). As shown in **Figure** **2**, all three SMAs showed similar Y‐shaped molecular geometry with a slight twist due to the interlocking structure with similar S···O = C intramolecular interactions. There was a slight increment in the distance from the S atom (on the external ring of the central core) to the O atom (on the end‐group) due to the substitution of the branched chains with the 10‐cyclohexyldecyl chains, which could also influence the molecular stacking. Moreover, the plane dihedral angles between the two furthest external thienyl rings of the central cores were 9.46° for L8‐BO, 2.04° for A‐C10ch, and 3.83° for Y‐C10ch, indicating that the 10‐cyclohexyldecyl chains were beneficial to the formation of efficient transport channels between adjacent molecules.

It is well known that the crystal structures of Y‐series SMAs are mainly formed by *π*–*π* interactions between end‐groups (A/A) or between the end‐group and core (A/D), resulting in the different crystal packing behaviors of the corresponding acceptors.^[^ ^61^, ^62^ ^]^ Therefore, the *π*–*π* stacking motifs of the four adjacent and independent molecular conformations corresponding to a single‐crystal are presented in Figure 2b, and different pairs of dimers are displayed in Figures S31–S33 in the Supporting Information. Compared with L8‐BO that has three types of dimers containing dimer A/A (end‐group parallel stacking), dimer A/A‐1 (end‐group antiparallel stacking) and dimer A/D‐1 (central core parallel stacking), the additional stacking motif of dimer A/D (central core antiparallel stacking) are observed in A‐C10ch and Y‐C10ch single‐crystals, which could contribute to the achievement of more hole transfer paths between adjacent molecules and the improvement of the 3D ambipolar transport network. Specifically, the *π*∙∙∙*π* distances of dimers A/A, A/A‐1, and A/D‐1 were estimated to be 3.47, 3.52, and 3.55 Å in the L8‐BO crystal, respectively. With the gradual incorporation of 10‐cyclohexyldecyl chains, the relevant *π*–*π* stacking distances decreased slightly in the single‐crystals of A‐C10ch (dimer A/A: 3.43 Å, dimer A/A‐1: 3.44 Å, dimer A/D‐1: 3.36 Å) and Y‐C10ch (dimer A/A: 3.36 Å, dimer A/A‐1: 3.41 Å, dimer A/D‐1: 3.36 Å). The reduced distances indicate stronger intermolecular interactions and may contribute to enhanced charge transport.

Further variations in the packing states induced by the 10‐cyclohexyldecyl are displayed in Figure 2c. Each elliptical frame structure of the A‐C10ch and Y‐C10ch crystals was formed under the cooperative interaction of six molecules, indicating highly ordered 3D molecular packing. These structures differ from that of the elliptical frame structure of the L8‐BO crystal with four molecules. This difference might be attributed to the unique properties of the 10‐cyclohexyldecyl chains, which can form a more planar structure since the less crowded linear alkyl component can produce inclination with a small angle and the rigid cyclic component can improve the interaction of adjacent molecular chains (Figure S34, Supporting Information). In addition, compared with L8‐BO, A‐C10ch, and Y‐C10ch both exhibited increased lateral and vertical distances (marked in Figure 2c) and thus gave rise to larger orthorhombic voids. These results reveal that the substitution of branched side chains with 10‐cyclohexyldecyl chains at the thiophene beta position can generate better planarity and tighter intermolecular packing, leading to redshifted absorption and more efficient vertical charge transport channels in the crystals.

To analyze the differences in transport properties induced by the branched and 10‐cyclohexyldecyl side‐chain patterns, the electronic couplings of all the *π*–*π* packing interactions in the single‐crystal networks of L8‐BO, A‐C10ch, and Y‐C10ch were calculated using density functional theory (B3LYP/6‐31G (d, p) basis set). As presented in Figure 2d, the electronic coupling values of the *π*–*π* stacking interactions between the two adjacent parallel end‐groups and adjacent reverse‐parallel end‐groups were 115.0 and 32.0 meV, respectively, in the Y‐C10ch crystal and 107.7 and 29.7 meV, respectively, in the A‐C10ch crystal. These values were greater than those in the L8‐BO crystal (2.3 and 23.8 meV, respectively), indicating shorter *π*–*π* packing distances and more efficient orbital overlap between the two adjacent end‐groups due to the side‐chain modification. A similar trend was observed in the electronic coupling values of the *π*–*π* packing interactions generated by the end‐group and central core in the adjacent reverse‐parallel molecules. Notably, Y‐C10ch and A‐C10ch had comparable |*J*| values of 302.9 and 296.1 meV, respectively, which were much greater than that of L8‐BO (0.8 meV). This may have originated from the different planarity of the main skeleton and the twist dihedral angle between the two furthest external thienyl rings of the central core. These results demonstrate that the 10‐cyclohexyldecyl side chains endowed the Y6‐series SMA single‐crystal with stronger *π*–*π* stacking, more efficient orbital overlap, and thus tighter 3D intermolecular packing networks, which contributed to the enhanced bulk crystallization and carrier transport properties in neat films.

### 2.3 Photovoltaic Characteristics and Carrier Mobility

**Figure 3:**
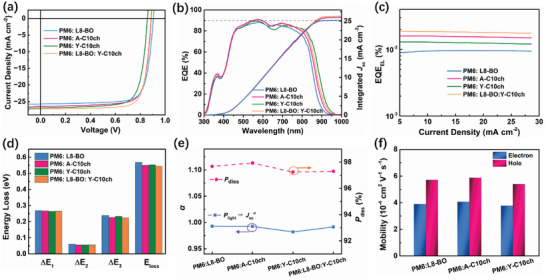
a) *J*–*V* curves of the optimal devices. b) Relevant EQE curves. c) EQE_EL_ of the optimized devices at different injected currents. d) Comparison of energy loss in the four types of devices. e) Dependences of *J*_sc_ on *P*_light_ and *J*_ph_ versus *V*_eff_. f) Hole and electron mobilities of the optimized blends.

The influence of the 10‐cyclohexyldecyl chain on the photovoltaic performances of the SMAs was further explored by fabricating OSCs with a device structure of indium tin oxide (ITO)/poly(3,4‐ethylenedioxythiophene): polystyrene sulfonate (PEDOT:PSS)/PM6:acceptor/poly[(9,9‐bis(3'‐(N,N‐dimethylamino)propyl)‐2,7‐fluorene)‐alt‐5,5′‐bis(2,2′‐thiophene)‐2,6‐naphthalene‐1,4,5,8‐tetracaboxylic‐N,N′‐di(2‐ethylhexyl)imide] (PNDIT‐F3N)/Ag. Optimization of the fabrication process and the relevant photovoltaic parameters are described in Tables S5–S14 in the Supporting Information. **Figure** **3**a presents the best current density–voltage (*J*–*V*) curves under optimal conditions and the corresponding device parameters are summarized in **Table** **2**. Although the PM6:A‐C10ch‐ and PM6:Y‐C10ch‐based devices showed slightly lower *V*_oc_ values (0.858 and 0.887 V, respectively) than the PM6:L8‐BO‐based devices (0.899 V), much higher *J*_sc_ values of 26.5 and 26.9 mA cm^−2^ were registered for PM6:A‐C10ch‐ and PM6:Y‐C10ch‐based devices respectively. This variation in device parameters corresponded to the reduced LUMO energy level and redshifted absorption edge of the SMAs. Furthermore, state‐of‐the‐art PCEs of 17.6%, 18.4%, and 18.1% were achieved by the PM6:Y‐C10ch‐, PM6:A‐C10ch‐, and PM6:L8‐BO‐based devices, respectively. To further regulate the trade‐off between *J*_sc_ and *V*_oc_ and improve device performance, Y‐C10ch was incorporated as the third component in the PM6:L8‐BO blend, which allowed the device to display a higher PCE of 19.1%, demonstrating the excellent application potential of hybrid cycloalkyl‐alkyl chains in constructing Y6‐series SMAs. As shown in Figure 3b, the external quantum efficiency (EQE) spectra of the devices based on the corresponding blends show broad EQEs of >80% from 450 to 850 nm. The integrated short circuit currents obtained from the EQE spectra match well (≈3% mismatch) with those from the *J*–*V* measurements.

**Table 2:** Optimized photovoltaic characteristics achieved by various OSCs under illumination of AM 1.5 G, 100 mW cm^−2^

Device	*J*_sc_ [mA cm^−2^]	*J*_sc_ ^a)^ [mA cm^−2^]	*V*_oc_ [V]	FF [%]	PCE^b)^ [%]
PM6:L8‐BO	25.6	25.1	0.899	78.8	18.1 (17.7 ± 0.4)
PM6:A‐C10ch	26.5	25.8	0.887	78.1	18.4 (18.0 ± 0.4)
PM6:Y‐C10ch	26.9	26.2	0.858	76.3	17.6 (17.3 ± 0.3)
PM6:L8‐BO:Y‐C10ch	27.2	26.4	0.886	79.2	19.1 (18.7 ± 0.4)

^a)^ Calculated from EQE curve

^b)^ The values in parentheses are the average values with standard deviations obtained from 15 devices.

Next, the *V*_oc_ losses in the OSCs induced by the 10‐cyclohexyldecyl chains were assessed using highly sensitive EQE (s‐EQE) and electroluminescence (EL) measurements.^[^ ^63^, ^64^ ^]^ The overall energy losses (*E*_loss_) of OSCs are caused by three factors: the charge recombination generated by unavoidable black body radiation (causing the Shockley–Queisser limit, Δ*E*_1_), radiative decay (Δ*E*_2_, radiative loss from below‐gap absorption), and nonradiative decay (Δ*E*_3_). Detailed *E*_loss_ analyses are presented in Figure 3c,d and **Table** **3**. There was no significant difference in Δ*E*_1_ for the slightly changed energy gaps of the three SMA devices. For radiative energy loss, both the Y‐C10ch‐ and A‐C10ch‐based devices exhibited slightly smaller Δ*E*_2_ (below‐gap; 0.056 eV) than the L8‐BO‐based device (0.060 eV), which may have originated from the steeper bandgap edge of the Y‐C10ch‐ and A‐C10ch‐based devices. By fitting the s‐EQE spectra beyond the bandgap edge with a linear equation, the inverse of the slope can be obtained and the Urbach energy (*E*_U_) can then be calculated (see Figure S35, Supporting Information).^[^ ^65^, ^66^ ^]^ Thus, the *E*_U_ values were calculated to be 23.9 and 24.0 meV for the symmetric Y‐C10ch‐ and L8‐BO‐based devices, respectively, which were higher than that of the asymmetric A‐C10ch‐based device (21.7 meV), indicating a lower energetic disorder in the PM6:A‐C10ch blend films.^[^ ^67^ ^]^ Furthermore, the nonradiative energy loss can be compared directly and calculated from EQE_EL_. Specifically, PM6:Y‐C10ch, PM6:A‐C10ch, and PM6:L8‐BO exhibited EQE_EL_ values of 1.2 × 10^−4^, 1.5 × 10^−4^, and 1.0 × 10^−4^, which corresponded to Δ*E*_3_ values of 0.233, 0.227, and 0.239 eV, respectively. Moreover, with the introduction of Y‐C10ch as a third component in the PM6:L8‐BO blend, the luminescence behavior and slope of linear fitting to the s‐EQE spectra beyond the bandgap edge increased, leading to smaller values of Δ*E*_2_ (0.054 eV) and Δ*E*_3_ (0.223 eV) compared with the binary blends. This indicates that the hybrid cycloalkyl‐alkyl chains are effective in lowering the total *E*_loss_ and improving the *V*_oc_ values without sacrificing photocurrent.

**Table 3:** Total energy losses and detailed energy losses of the optimized devices

Active layer	*E*_g_ [eV]	*V*_oc_ [V]	*E*_loss_ [eV]	*V*SQ oc [V]	Δ*E*_1_ [eV]	*V*rad oc [V]	Δ*E*_2_ [eV]	Δ*E*_3_ [eV]	EQE_EL_ [%]
PM6:L8‐BO	1.464	0.899	0.568	1.198	0.269	1.138	0.060	0.239	0.010
PM6:A‐C10ch	1.437	0.887	0.550	1.170	0.267	1.114	0.056	0.227	0.015
PM6:Y‐C10ch	1.409	0.858	0.553	1.144	0.265	1.088	0.056	0.233	0.012
PM6:L8‐BO:Y‐C10ch	1.429	0.886	0.544	1.163	0.267	1.109	0.054	0.223	0.018

Apart from the variation in *E*_loss_, it is crucial to reveal the reasons for the high *J*_sc_ and FF of the OSCs fabricated using the 10‐cyclohexyldecyl chain‐modified acceptors from the perspective of charge extraction and recombination.^[^ ^68^ ^]^ The effective voltage (*V*_eff_) versus saturated photocurrent density (*J*_ph_) measurements of all devices showed that the exciton dissociation probability *P*(*E*, *T*) values were ≈98% (Figure S36, Supporting Information). Furthermore, the assessed charge recombination behaviors tested by the relation between *J*_sc_ and different light intensities (*P*_light_) yielded a similar slope (*S*) of ≈1 in the equation of *J*_sc_ ∝ *P^S^* (Figure S37, Supporting Information). These results suggest that excellent exciton dissociation ability and negligible bimolecular recombination loss occurred in the PM6:L8‐BO, PM6:Y‐C10ch, PM6:A‐C10ch, and PM6:L8‐BO:Y‐C10ch devices, which were responsible for the high *J*_sc_ and FF values generated because of the incorporation of symmetric/asymmetric 10‐cyclohexyldecyl chains in the SMAs.

To elucidate the effect of the 10‐cyclohexyldecyl chains on charge mobility, space‐charge‐limited current measurements were conducted to measure the electron mobility (*µ*_e_) of the pure acceptors and hole mobility (*µ*_h_) and *µ*_e_ of the blends (Figures S38–S40 and Table S4, Supporting Information). For the pure films, the stepwise substitution of 10‐cyclohexyldecyl chains from L8‐BO to A‐C10ch and Y‐C10ch caused an increase in *µ*_e_, which is accordant with the tighter molecular packing and highly connected networks demonstrated by the single‐crystal structure and GIWAXS measurements. After blending with PM6, the L8‐BO, A‐C10ch, and Y‐C10ch blend films showed comparable *µ*_h_ and *µ*_e_, accompanied by balanced *µ*_h_/*µ*_e_ ratios of 1.46, 1.47, and 1.38, respectively, which contributed to the prevention of charge accumulation and recombination, endowing the corresponding devices with high *J*_sc_ and FF values.

### 2.4 Photodynamic Measurements

**Figure 4:**
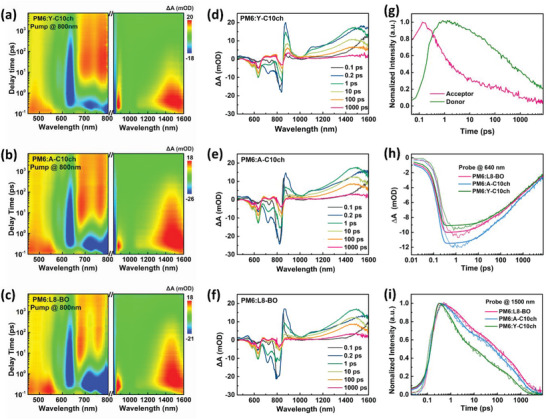
Color plots of the TA spectra of these three blends (a, b, c) in the range of 460–800 nm and in the range of 820–1600 nm under excitation wavelengths of 800 nm. Extracted plot in TA spectra of these three blends (d, e, f) at selective delay times. g) TA kinetics in blended films showing the electron transfer process. h) TA traces of the three blends probed at 640 nm (800 nm excitation). i) TA traces of the three blends probed at 1500 nm (800 nm excitation).

To further investigate the interfacial exciton dissociation and recombination dynamics, femtosecond transient absorption (TA) spectroscopy was applied to the L8‐BO, A‐C10ch, and Y‐C10ch systems. The 2D TA images and relevant spectra with varying decay times are displayed in **Figure** **4**a–f. An excitation wavelength of 800 nm was used to excite the acceptors of the D:A blends selectively. The similar spectra can be observed for these blend systems that with the decay of acceptor ground‐state‐bleach (GSB) peaks at 700–800 nm. The GSB peak of PM6 appeared at ≈640 nm and increased in the first 50 ps, which can be assigned to hole transfer dynamics from the acceptor to PM6 (Figure 4g), generating the charge transfer (CT) state in the blend. By fitting the hole transfer kinetics with a biexponential function, the L8‐BO‐, A‐C10ch‐, and Y‐C10ch‐based blends exhibited fast *τ*_1_ values of 0.26, 0.29, and 0.37 ps, respectively, and slow *τ*_2_ values of 13.2, 15.3, and 20.8 ps, respectively. In general, the ultrafast *τ*_1_ is commonly deemed as the time required for dissociation of the acceptor exciton occurring at the D:A interface and the relatively slow *τ*_2_ is attributed to the time required for exciton diffusion toward the interface before dissociation.^[^ ^69^ ^]^ The results show that introduction of 10‐cyclohexyldecyl chains slightly reduced the exciton transfer rate at the D:A interface, whereas it greatly extended a proportion of the exciton diffusion‐mediated transfer process, which could be associated with the presence of larger pure acceptor domains and a reduced mixed‐phase (vide infra). Furthermore, the GSB peak of PM6 at 636 nm decayed the slowest in the A‐C10ch blend, which indicates that the PM6:A‐C10ch blend exhibited the longest CT state lifetime. The relatively long CT state lifetime may be related to the low energy loss, with a controlled trade‐off between the *J*_sc_ and FF values.^[^ ^70^ ^]^ The exciton diffusion length (*L*_D_) in acceptors was further investigated via the exciton–exciton annihilation method with an excitation wavelength of 800 nm. The decay curves with various excitation fluences are shown in Figure S41 in the Supporting Information, and the relevant parameters are given in Table S15 in the Supporting Information. The similar *L*_D_ values (≈6.2 nm) of the three blend films indicate that A‐C10ch and Y‐C10ch (with asymmetric/symmetric 10‐cyclohexyldecyl chain patterns) had a negligible effect on the diffusion distance during the effective exciton lifetime compared with L8‐BO (with branched chain patterns).

Next, we probed the TA traces of the three blend films at 1500 nm, which represents the generation of the intra‐moiety excited state (i‐EX) from photoexcited locally excited states due to intermolecular interactions in the acceptor phase (Figure 4h). Interestingly, the PM6:Y‐C10ch film exhibited a much shorter i‐EX lifetime (36 ps) than the PM6:A‐C10ch (171 ps) and L8‐BO (223 ps) films, which might be associated with the slower charge transfer at the PM6:Y‐C10ch interface and stronger intermolecular binding energy. Hence, it is suggested that the variations of the side chain (from branched to cycloalkyl‐alkyl‐type) in the thiophene beta position can have a dramatic effect on the i‐EX state lifetimes, which probably arises from the regulation of electronic coupling caused by the different dimers and *π*–*π* stacking states.

### 2.5 Morphological Characterization

**Figure 5:**
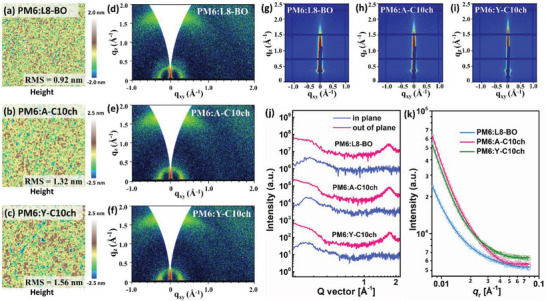
a–c) AFM images of the optimal blend films. d–f) 2D GIWAXS images of the corresponding films. g–i) 2D‐GISAXS images of the corresponding films. j) GIWAXS intensity profiles of the corresponding films along the IP and OOP directions. k) GISAXS intensity profiles of the corresponding films along the *q*_r_ axis.

To understand how the symmetric/asymmetric 10‐cyclohexyldecyl chain modalities influence the morphology and crystallization of the bulk heterojunction films, the blend films were characterized using atomic force microscopy (AFM), 2D‐GIWAXS, and 2D grazing‐incidence small‐angle X‐ray scattering (2D‐GISAXS). As presented in **Figure** **5**a–c, the height images show that all the blends formed compatible surface morphology with a gradual increase in root‐mean‐square roughness (0.92 nm for PM6:L8‐BO, 1.32 nm for PM6:A‐C10ch, and 1.56 nm for PM6:Y‐C10ch), suggesting that the 10‐cyclohexyldecyl chain patterns could alter nanoscale aggregates in the blend films. These results are consistent with the transmission electron microscopy (TEM) images, in which more‐defined phase separation with an interpenetrating network could be observed (Figure S42, Supporting Information).

The 2D GIWAXS patterns, IP and OOP 1D profiles, and *d*‐spacing of the blends are summarized in Figure 5d–f,j and Table S2 in the Supporting Information. All three blend films (PM6:L8‐BO, PM6:A‐C10ch, and PM6:Y‐C10ch) adopted a similar face‐on orientation with strong (010) diffraction peaks at *q* = 1.74, 1.76, and 1.77 Å^−1^, respectively, in the OOP direction, corresponding to the decreased *π*–*π* stacking distances of 3.61, 3.57, and 3.55 Å, respectively. Although the *π*–*π* stacking coherence lengths (CL_010_) of the three blend films were in the range of 25–28 Å, there was a large difference in the lamellar stacking coherence lengths (CL_100_), with the increased parameters of 72.8 Å for PM6:L8‐BO, 102.4 Å for PM6:A‐C10ch, and 123.7 Å for PM6:Y‐C10ch, suggesting that the 10‐cyclohexyldecyl chain moieties could endow the blend film microstructure with tighter inter‐chain packing. Thus, the PM6:A‐C10ch‐ and PM6:Y‐C10ch‐based blends showed larger crystalline domains due to the side chain modification, which is consistent with the AFM and TEM images.

2D‐GISAXS measurements were carried out to provide a more in‐depth understanding of the differences between the three blends (Figure 5g–i). The phase separation information of the amorphous phases and crystalline domains was estimated by the Debye–Anderson–Brumberger and fractal‐like network models (Figure 5k). ^[^ ^71^ ^]^ The intermixing domain spacings (*ξ*) of the L8‐BO‐, A‐C10ch‐, and Y‐C10ch‐based blends were estimated to be 28.2, 26.2, and 24.0 nm, respectively. Among the three SMAs, the smallest *ξ* value of the Y‐C10ch blend film was associated with the largest extent of crystallization. Moreover, the crystalline domain sizes of the L8‐BO, A‐C10ch, and Y‐C10ch blends were calculated to be 11.2, 13.9, and 16.0 nm, respectively, all of which fell within the range of ideal exciton diffusion lengths (10–20 nm), and thus contributed to the large *J*_sc_ and FF values of the OSCs. By combination of the above morphological results, we propose that the asymmetric/symmetric 10‐cyclohexyldecyl chain patterns have reduced steric hindrance and maintain feasible molecular packing compared with the branched side chains, which could lead to appropriate phase separation in the blend films.

## 3 Conclusion

We designed and synthesized new symmetric/asymmetric SMAs (A‐C10ch and Y‐C10ch) by introducing bilateral and unilateral 10‐cyclohexyldecyl chains and demonstrated that the use of grafted side‐chain engineering offers notable control over the molecular packing, single‐crystal structure, and blend film morphology. With the stepwise addition of hybrid side chains (from L8‐BO to A‐C10ch and Y‐C10ch), the neat films presented stepwise narrowed optical bandgaps, gradually reduced energy levels, and steadily promoted electron mobility. Compared with L8‐BO with branched alkyl chains, the single‐crystal structures of the SMAs with 10‐cyclohexyldecyl side chain exhibit different 3D network packing patterns, tighter *π*–*π* stacking distance along the conjugated backbones, and enhanced intermolecular *π*–*π* electronic coupling and orbital overlap, which contributed to stronger crystallization in the corresponding neat films. Photovoltaic measurements revealed that remarkable PCEs of 17.6–18.4% were obtained with these OSCs with trade‐off parameters. The *V*_oc_ values in the PM6:L8‐BO‐based devices were enhanced, whereas those of the PM6:A‐C10ch‐ and PM6:Y‐C10ch‐based OSCs were compromised and the *J*_sc_ values in the PM6:A‐C10ch‐ and PM6:Y‐C10ch‐based OSCs were improved. These parameter variations could be attributed to the interfacial exciton with controlled hole transfer dynamic and intramoiety excited state, slightly reduced energy loss and tunable nanofibrous morphology caused by the 10‐cyclohexyldecyl side chains. Furthermore, using the Y‐C10ch molecule as a third component in the PM6:L8‐BO blend produced a PCE of 19.10% (one of the highest efficiencies reported in single‐junction OSCs), which may further lead to higher performance OSCs. This contribution demonstrates that hybrid cycloalkyl‐alkyl chains as a new composite side‐chain strategy are a feasible and potentially useful building block for improving the intermolecular crystal packing of SMAs, readjusting the interfacial exciton properties, and expanding the development of new materials in the field of organic optoelectronics.

## Conflict of Interest

The authors declare no conflict of interest.

## Supporting information

Supporting information

Supporting information

Supporting information

## Data Availability

The data that support the findings of this study are available in the supplementary material of this article.
