# Supplementary material for: Hybrid Cycloalkyl‐Alkyl Chain‐Based Symmetric/Asymmetric Acceptors with Optimized Crystal Packing and Interfacial Exciton Properties for Efficient Organic Solar Cells
Source: Adv Sci (Weinh). 2023 Jan 2;10(7):2206580. doi: 10.1002/advs.202206580 (PMC9982590; doi:10.1002/advs.202206580)
Supplement: Supplementary file 1 — Supporting information [file ADVS-10-2206580-s002.pdf]

ADVANCED SCIENCE
Open Access

Supporting Information

for *Adv. Sci.*, DOI 10.1002/advs.202206580

Hybrid Cycloalkyl-Alkyl Chain-Based Symmetric/Asymmetric Acceptors with Optimized Crystal Packing and Interfacial Exciton Properties for Efficient Organic Solar Cells

Cong Xiao, Xunchang Wang, Tian Zhong, Ruixue Zhou, Xufan Zheng, Yirui Liu, Tianyu Hu, Yixuan Luo, Fengbo Sun, Biao Xiao, Zhitian Liu*, Chunming Yang and Renqiang Yang**

Supporting Information

Hybrid cycloalkyl-alkyl chain-based symmetric/asymmetric acceptor with optimized crystal packing and interfacial exciton properties for efficient organic solar cells

Cong Xiao, Xunchang Wang, Ruixue Zhou, Xufan Zheng, Yirui Liu, Tianyu Hu, Shiyun Lei, Fengbo Sun, Tian Zhong, Biao Xiao, Zhitian Liu,* Chunming Yang, and Renqiang Yang**

C. Xiao, Dr. X. Wang, T. Zhong, Dr. R. Zhou, X. Zheng, Y. Liu, T. Hu, Dr. B. Xiao, Prof. Dr. R. Yang
Key Laboratory of Optoelectronic Chemical Materials and Devices (Ministry of Education), School of Optoelectronic Materials & Technology, Jianghan University, Wuhan 430056, China
E-mail: wangxc@jhun.edu.cn, yangrq@jhun.edu.cn

Y. Luo, F. Sun, Prof. Dr. Z. Liu
Hubei Engineering Technology Research Center of Optoelectronic and New Energy Materials, Wuhan Institute of Technology, Wuhan 430205, China
E-mail: able.ztliu@wit.edu.cn

Prof. Dr. C. Yang
Shanghai Synchrotron Radiation Facility, Shanghai Advanced Research Institute, Chinese Academy of Sciences, Shanghai 201204, China

Dr. X. Wang
State Key Laboratory of Fine Blasting, Jianghan University, Wuhan 430056, China

1. Materials and Methods. All the reagents, unless otherwise specified, were purchased from Sigma-Aldrich Co., J&K, and Tokyo Chemical Industry Co., Ltd., and were used without further purification. PM6 was purchased from Solarmer Materials Inc. All air and water-sensitive reactions were carried out under N_2. Toluene and THF were dried by Na and then freshly distilled before use. The detailed synthetic procedures are as follows.

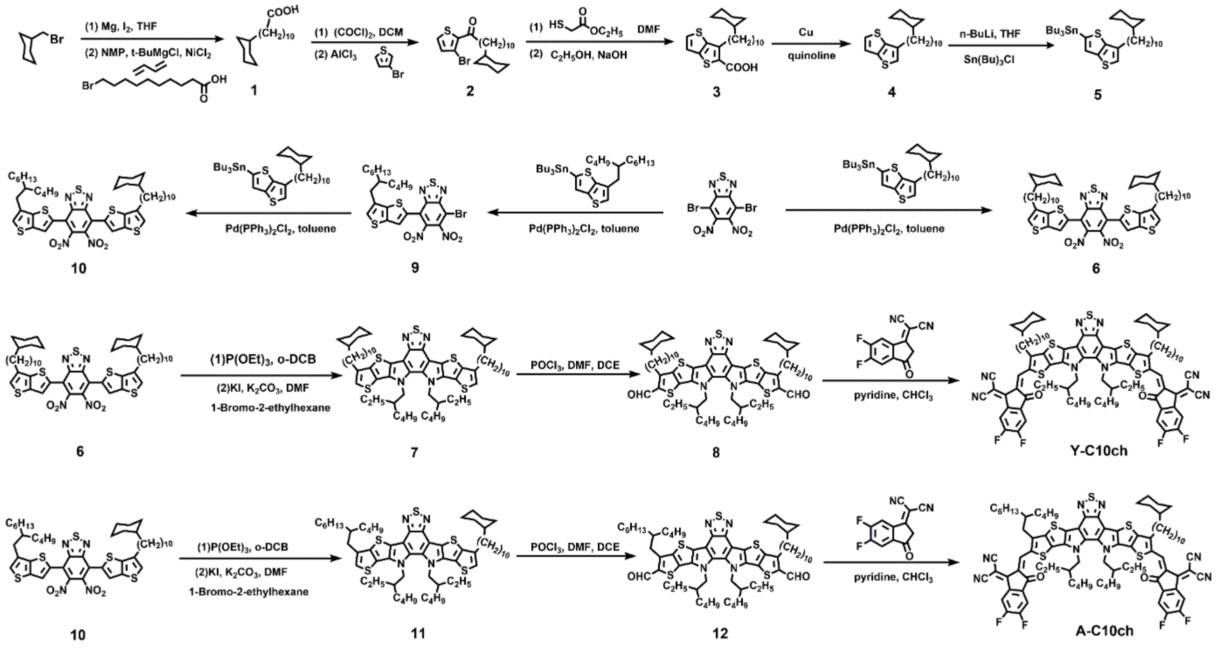

Scheme S1. The synthetic routes of Y-C10ch and A-C10ch.

Synthesis of compound 1

Under the protection of N_2 atmosphere, magnesium powder (4.7 g, 196 mmol), catalytic amount of I_2 (20 mg) and 10 mL of anhydrous THF were added into a three-necked round flask. The solution of bromomethylcyclohexane (24.8 g, 140 mmol) in THF (50 mL) was slowly dropped in the flask. The reaction mixture was refluxed for 4 h until the magnesium was fully consumed. After the reaction was cooled down to ambient temperature, the corresponding Grignard reagent was prepared.

tBuMgCl (100 mL, 1M in THF) was added into a two-necked round flask that contained a 10-bromodecanoic acid (25.1 g, 100 mmol), NMP (9.9 g, 100 mmol), and THF (150.0 mL)

at -78 °C and the mixture was stirred for 10 min under N_2 atmosphere. The Grignard reagent that was prepared, 1,3-butadiene (50 mL, 2M in THF), and $NiCl_2$ (65 mg) were added at the same temperature and the mixture was stirred with cooling in an ice-bath for 1 h. The reaction was quenched by the addition of aqueous HCl and the resulting solution was extracted with CH_2Cl_2. The combined extract was concentrated under reduced pressure, and purified by column chromatography on silica gel to yield 1 as a white solid (24.5 g, 91.3%). ^{1}H NMR (400 MHz, Chloroform-*d*): δ 2.35 (t, J = 7.5 Hz, 2H), 1.76-1.54 (m, 7H), 1.38-1.11 (m, 20H), 0.92-0.77 (m, 2H). ^{13}C NMR (101 MHz, Chloroform-*d*): δ 179.71, 37.69, 37.56, 33.99, 33.47, 29.98, 29.67, 29.59, 29.44, 29.24, 29.06, 26.87, 26.79, 26.47, 24.69.

Synthesis of compound 2

To a solution of compound 1 (24.5 g, 91.3 mmol) in CH_2Cl_2 (150 mL) was added oxalyl chloride (34.8 g, 274 mmol) at 0 °C. The mixture was stirred for 5 h at room temperature. After removal of the solvents and remaining oxalyl chloride, the crude product was then used directly for the next step.

To a stirring mixture of 3-bromothiophene (14.9 g, 91.3 mmol) and the prepared acid chloride (91.3 mmol) in dichloromethane, $AlCl_3$ (12.2 g, 91.3 mmol) was added in portions over 30 min. The resulting solution was stirred for 4 h at room temperature. Cold water (300 mL) was then slowly added to quench the reaction. The crude product was extracted from the mixture with CH_2Cl_2. The combined extract was dried over Na_2SO_4 and concentrated under reduced pressure, and purified by column chromatography on silica gel to yield 2 as orange liquid (20.8 g, 55% yield). ^{1}H NMR (400 MHz, Chloroform-*d*): δ 7.49 (d, J = 5.2 Hz, 1H), 7.10 (d, J = 5.2 Hz, 1H), 3.02 (t, J = 7.4 Hz, 2H), 1.69 (m, 7H), 1.32-1.17 (m, 20H), 0.86 (m,

2H). ^{13}C NMR (101 MHz, Chloroform-d): δ 192.78, 138.67, 133.62, 131.66, 130.58, 113.68, 41.56, 37.69, 37.57, 33.48, 30.00, 29.69, 29.61, 29.50, 29.45, 29.24, 26.88, 26.80, 26.48, 24.19.

Synthesis of compound 3

Compound 2 (20.6 g, 50 mmol) and K_2CO_3 (13.8 g, 100 mmol) were mixed with DMF (120 mL). To this mixture, ethyl thioglycolate (6.0 g, 50 mmol) was added dropwise under N_2 atmosphere at 60 °C. The mixture was stirred overnight and poured into water (200 mL). The organic material was extracted with CH_2Cl_2. The combined organic layers were washed with brine and dried over anhydrous Na_2SO_4. The crude product of compound 3 was obtained by removing organic solvents and used directly for the next step without further purification.

To a mixture solution of compound 3 in ethanol (160 mL), NaOH (4.0 g, 100 mmol) was added. The resulting solution was refluxed overnight. After cooling to room temperature, the liquid was acidified with concentrated HCl. A yellow solid was collected by filtration and washed several times with water. The solid was then washed three times with petroleum ether to give 3 as a light pink solid (9.8 g, 48% yield). ^{1}H NMR (400 MHz, Chloroform-*d*): δ 7.60 (d, J = 5.2 Hz, 1H), 7.27 (d, J = 5.2 Hz, 1H), 3.24-3.15 (m, 2H), 1.75 (m, 2H), 1.67 (m, 5H), 1.39-1.12 (m, 20H), 0.88-0.80 (m, 2H). ^{13}C NMR (101 MHz, Chloroform-*d*): δ 168.42, 145.15, 142.00, 141.67, 131.64, 126.87, 120.10, 37.70, 37.58, 33.48, 30.02, 29.72, 29.68, 29.65, 29.55, 29.41, 29.30, 26.90, 26.80, 26.48.

Synthesis of compound 4

A mixture of compound 3 (9.5 g, 23.4 mmol), copper power (1.5 g) and quinolone (100 mL) was heated under N_2 atmosphere at 260 °C in a Woods-metal bath. After 3 h, the mixture

was cooled to room temperature and hexane (200 mL) was added to the quinoline mixture. This mixture was then washed with HCl. The organic layer was dried over anhydrous Na_2SO_4 and the solvent was removed. Compound 4 was obtained by chromatography on silica gel, eluting with petroleum ether (7.9 g, 93.1% yield). ^{1}H NMR (400 MHz, Chloroform-*d*): δ 7.34 (dd, J = 5.2, 1.6 Hz, 1H), 7.23 (d, J = 5.2 Hz, 1H), 6.98 (s, 1H), 2.72 (t, J = 7.8 Hz, 2H), 1.80-1.62 (m, 7H), 1.37-1.14 (m, 20H), 0.89-0.83 (m, 2H). ^{13}C NMR (101 MHz, Chloroform-*d*): δ 139.98, 138.71, 134.91, 126.55, 121.71, 119.92, 37.71, 37.59, 33.50, 30.02, 29.98, 29.72, 29.67, 29.59, 29.42, 29.39, 28.62, 26.90, 26.81, 26.50.

Synthesis of compound 5

To a solution of compound 4 (7.8 g, 21.5 mmol) in tetrahydrofuran (120 mL), 2.4 M n-butyllithium (9.0 mL, 21.5 mmol) was added dropwise under N_2 atmosphere at -78 °C. After stirring at -78 °C for 1 h, tributylstannyl chloride (7.0 g, 21.5 mmol) was added to the mixture at -78 °C, and the mixture was gradually warmed to room temperature. After stirring overnight, the reaction was quenched with water (100 mL). The mixture was extracted with petroleum ether, and the organic layer was dried over Na_2SO_4. Removing the solvent under reduced pressure gave the crude compound 5. Without any further purification, the product was used into the following reaction.

Synthesis of compound 6

Into a 250 mL two-neck round-bottom flask, compound **5** (5.86 g, 9.0 mmol), 4,7-dibromo-5,6-dinitro[2,1,3]benzothiadiazole (1.15 g, 3.0 mmol) and $Pd(PPh_3)Cl_2$ (0.12 g, 0.18 mmol) were added. The flask was evacuated and back-filled with N_2 three times, and then freshly distilled toluene (80 mL) was injected into the mixture. The resulting reaction mixture

was refluxed for 12 h under N_2 atmosphere. The reaction mixture was allowed to cool to room temperature and then concentrated under reduced pressure. The crude product was chromatographically purified on a silica gel column eluting with petroleum ether/dichloromethane (8:1) to afford compound **6** as a red solid (1.61 g, 56.7% yield). ^{1}H NMR (400 MHz, Chloroform-*d*): δ 7.97 (s, 2H), 7.44 (s, 2H), 3.04 (t, J = 7.7 Hz, 4H), 2.05 (m, 4H), 1.97-1.88 (m, 10H), 1.66-1.40 (m, 40H), 1.12 (m, 4H). ^{13}C NMR (101 MHz, Chloroform-d): δ 152.22, 144.27, 141.79, 139.09, 135.12, 130.07, 125.18, 124.10, 121.44, 37.69, 37.57, 33.48, 30.00, 29.83, 29.70, 29.65, 29.58, 29.38, 28.57, 26.89, 26.79, 26.48.

***Synthesis of compound** 7*

In a 50 mL two-neck round-bottom flask, compound **6** (0.32 g, 0.338 mmol), triethyl phosphite (3.5 mL), and 1,2-dichlorobenzene (*o*-DCB, 1.0 mL) were added, and then evacuated and back-filled with N_2 three times. The resulting reaction mixture was refluxed overnight under N_2 atmosphere. After cooling to room temperature, the mixture was quenched with saturated aqueous solution of ammonium chloride and then extracted with dichloromethane. The organic layers were combined and dried over anhydrous Na_2SO_4. After removal of the solvent, the crude product was obtained as red oil and could be directly used for further reactions without any purification. The red intermediate was transferred to a 100 mL two-neck flask and potassium carbonate (0.47 g, 3.38 mmol), potassium iodide (22 mg, 0.135 mmol), and 1-bromo-2-ethylhexane (0.656 g, 3.4 mmol) were added. The flask was evacuated and back-filled with N_2 three times, and then freshly distilled DMF (5.0 mL) was injected into the mixture. The resulting reaction mixture was stirred at 90 °C for 12 h under N_2 atmosphere. After cooling to room temperature, the mixture was quenched with saturated

aqueous solution of ammonium chloride and then extracted with ethyl acetate. The organic layers were combined and washed with saturated brine solution and dried over anhydrous Na_2SO_4. After removal of the solvent, the crude product was purified by silica gel column chromatography, eluting with petroleum ether/dichloromethane (8:1) to obtain the product as red solid (150 mg, 40.1%). ^{1}H NMR (400 MHz, Chloroform-d): δ 7.01 (s, 2H), 4.67-4.53 (m, 4H), 2.82 (t, J = 7.7 Hz, 4H), 2.03 (m, 2H), 1.86 (m, 4H), 1.73-1.62 (m, 10H), 1.34-1.13 (m, 46H), 0.91-0.83 (m, 14H), 0.68-0.56 (m, 12H). ^{13}C NMR (101 MHz, Chloroform-*d*): δ 147.68, 142.04, 137.03, 136.87, 131.47, 123.64, 122.55, 119.25, 111.46, 54.89, 39.90, 37.70, 37.58, 33.48, 30.02, 29.71, 29.68, 29.65, 29.62, 29.51, 29.47, 28.83, 27.68, 27.66, 26.90, 26.80, 26.48, 23.09, 23.04, 22.73, 14.13, 13.70, 10.04, 10.00.

Synthesis of compound 8

To a solution of compound **7** (0.16 g, 0.144 mmol) in freshly distilled 1,2-dichloroethane (10 mL), the mixture of freshly distilled DMF (3.0 mL) and $POCl_3$ (1.0 mL) was added dropwise under N_2 atmosphere at 0 °C. After addition, the cooling bath was removed and the reaction mixture was refluxed at 80 °C for 4 h. The mixture was quenched with saturated aqueous solution of sodium acetate, stirred for another 1 h, and the reaction mixture was then extracted with dichloromethane. The organic layers were combined and washed with saturated brine solution and dried over anhydrous Na_2SO_4. After removal of the solvent, the crude product was purified by silica gel column chromatography, eluting with petroleum ether/dichloromethane (1:1) to obtain the product as orange solid (0.12 g, 71.6%). ^{1}H NMR (400 MHz, Chloroform-*d*): δ 10.14 (s, 2H), 4.69-4.58 (m, 4H), 3.20 (t, *J* = 7.7 Hz, 4H), 1.96 (m, 6H), 1.67 (m, 10H), 1.52-1.06 (m, 46H), 0.96-0.81 (m, 14H), 0.69-0.58 (m, 12H). ^{13}C

NMR (101 MHz, Chloroform-*d*): δ 181.81, 147.54, 146.89, 143.15, 137.02, 136.81, 132.75, 129.64, 127.22, 112.38, 55.16, 40.12, 37.68, 37.56, 33.47, 30.34, 29.98, 29.67, 29.63, 29.53, 29.37, 28.17, 27.51, 26.88, 26.78, 26.47, 23.08, 22.68, 13.65, 10.10, 10.05.

Synthesis of compound Y-C10ch

In a 100 mL two-neck round-bottom flask, compound **8** (85 mg, 0.073 mmol) and 2-(5, 6-difluoro-3-oxo-2,3-dihydro-1*H*-inden-1-ylidene)malononitrile (130 mg, 0.565 mmol) were added. The reaction mixture was evacuated and backfilled with N_2 three times, and then freshly degassed chloroform (15 mL) and pyridine (0.5 mL) were added the reaction mixture was stirred at room temperature for 12 h. Then the mixture was poured into methanol and the precipitate was filtered off and washed with methanol. The crude product was purified by silica gel column chromatography, eluting with petroleum ether/dichloromethane (1:2) to give the product as blue-black solid (80 mg, 69.0%). ^{1}H NMR (400 MHz, Chloroform-*d*): δ 9.14 (s, 2H), 8.55 (dd, *J* = 9.9, 6.4 Hz, 2H), 7.71 (t, *J* = 7.5 Hz, 2H), 4.85-4.71 (m, 4H), 3.22 (m, 4H), 2.16-2.07 (m, 2H), 1.92-1.81 (m, 4H), 1.72-1.60 (m, 10H), 1.55-1.46 (m, 4H), 1.39-1.00 (m, 52H), 0.88-0.72 (m, 10H), 0.71-0.62 (m, 6H). ^{13}C NMR (101 MHz, Chloroform-*d*): δ 186.16, 158.79, 153.92, 147.52, 145.17, 137.76, 135.95, 135.26, 133.86, 133.23, 130.61, 119.96, 115.06, 114.95, 114.85, 114.55, 113.62, 68.73, 55.67, 40.40, 37.69, 37.56, 33.47, 31.21, 30.01, 29.83, 29.70, 29.65, 29.52, 29.45, 27.70, 27.66, 26.90, 26.78, 26.47, 23.25, 22.83, 13.75, 10.24, 10.18.

Synthesis of compound 9

In a 100 mL two-neck round-bottom flask, 4,7-dibromo-5,6-dinitro[2,1,3]benzothiadiazole (1.15 g, 3.0 mmol), tributyl(6-(2-butyloctyl)thieno[3,2-

b]thiophen-2-yl)stannane$_{S1}$ (0.896 g, 1.5 mmol) and $Pd(PPh_3)_2Cl_2$ (84.2 mg, 0.12 mmol) were added. The flask was evacuated and back-filled with N_2 three times, and then freshly distilled toluene (20 mL) was injected into the mixture. The resulting reaction mixture was kept at 60 °C for 12 h under N_2 atmosphere. The reaction mixture was allowed to cool to room temperature and then concentrated under reduced pressure. The crude product was chromatographically purified on a silica gel column eluting with petroleum ether/dichloromethane (8:1) as the eluent to afford compound **9** as a red solid (0.85 g, 46.3 % yield). ^{1}H NMR (400 MHz, Chloroform-*d*): δ 7.71 (s, 1H), 7.15 (s, 1H), 2.71 (d, *J* = 7.0 Hz, 2H), 1.87 (s, 1H), 1.35-1.25 (m, 16H), 0.91-0.85 (m, 6H).

Synthesis of compound 10

In a 100 mL two-neck round-bottom flask, compound **10** (0.85 g, 1.39 mmol), compound **5** (1.36 g, 2.08 mmol) and $Pd(PPh_3)_2Cl_2$ (58.4 mg, 0.08 mmol) were added. The flask was evacuated and back-filled with N_2 three times, and then freshly distilled toluene (20 mL) was injected into the mixture. The resulting reaction mixture was refluxed for 12 h under N_2 atmosphere. The reaction mixture was allowed to cool to room temperature and then concentrated under reduced pressure. The crude product was chromatographically purified on a silica gel column eluting with petroleum ether/dichloromethane (8:1) to obtain the product as red solid (0.91 g, 73.3%). ^{1}H NMR (400 MHz, Chloroform-*d*): δ 7.71 (s, 2H), 7.17 (s, 1H), 7.15 (s, 1H), 2.78 (t, *J* = 7.7 Hz, 2H), 2.71 (d, *J* = 7.1 Hz, 2H), 1.89-1.84 (m, 1H), 1.81-1.75 (m, 2H), 1.71-1.61 (m, 5H), 1.33-1.25 (m, 24H), 1.20-1.10 (m, 6H), 0.91-0.83 (m, 12H). ^{13}C NMR (101 MHz, Chloroform-*d*): δ 152.22, 144.64, 144.26, 141.80, 139.09, 139.02, 135.12, 134.26, 130.08, 125.96, 125.17, 124.09, 121.47, 121.41, 37.70, 37.57, 37.30, 34.69, 33.57,

33.48, 33.27, 31.89, 30.01, 29.84, 29.70, 29.65, 29.63, 29.58, 29.38, 28.79, 28.57, 26.89, 26.79, 26.53, 26.48, 23.04, 22.69, 14.14.

Synthesis of compound 11

In a 50 mL two-neck round-bottom flask, compound **10** (0.90 g, 1.01 mmol), triethyl phosphite (6.0 mL) and 1,2-dichlorobenzene (*o*-DCB, 6.0 mL) were added, and then evacuated and back-filled with N_2 three times. The resulting reaction mixture was refluxed overnight under N_2 atmosphere. After cooling to room temperature, the mixture was quenched with saturated aqueous solution of ammonium chloride and then extracted with dichloromethane. The organic layers were combined and dried over anhydrous Na_2SO_4. After removal of the solvent, the crude product was obtained as red oil and could be directly used for further reactions without any purification. The red intermediate was transferred to a 100 mL two-neck flask and potassium carbonate (1.39 g, 10.1 mmol) and potassium iodide (65.5 mg, 0.40 mmol), 1-bromo-2-ethylhexane (1.95 g, 10.1 mmol) were added. The flask was evacuated and back-filled with N_2 three times, and then freshly distilled DMF (5.0 mL) was injected into the mixture. The resulting reaction mixture was stirred at 90 °C for 12 h under N_2 atmosphere. After cooling to room temperature, the mixture was quenched with saturated aqueous solution of ammonium chloride and then extracted with ethyl acetate. The organic layers were combined and washed with saturated brine solution and dried over anhydrous Na_2SO_4. After removal of the solvent, the crude product was purified by silica gel column chromatography, eluting with petroleum ether/dichloromethane (8:1) to obtain the product as red solid (520 mg, 48.4%). ^{1}H NMR (400 MHz, Chloroform-*d*): δ 7.01 (s, 1H), 6.99 (s, 1H), 4.66-4.54 (m, 4H), 2.82 (t, J = 7.7 Hz, 2H), 2.75 (d, J = 7.2 Hz, 2H), 2.09-1.98 (m, 3H), 1.90-

1.81 (m, 2H), 1.73-1.63 (m, 5H), 1.37-1.24 (m, 30H), 1.19-1.03 (m, 10H), 0.93-0.84 (m, 20H), 0.66-0.57 (m, 12H). ^{13}C NMR (101 MHz, Chloroform-*d*): δ 147.69, 142.31, 142.03, 137.08, 137.03, 136.87, 136.02, 131.43, 123.64, 123.55, 122.53, 120.06, 119.24, 111.49, 111.45, 54.90, 39.91, 39.88, 37.70, 37.58, 37.40, 34.68, 33.71, 33.48, 33.39, 31.89, 30.02, 29.74, 29.72, 29.68, 29.65, 29.63, 29.51, 29.47, 28.83, 27.64, 26.90, 26.80, 26.58, 26.48, 23.10, 22.82, 22.74, 22.69, 14.13, 13.69, 10.06.

Synthesis of compound 12

To a solution of compound **11** (0.5 g, 0.474 mmol) in freshly distilled 1,2-dichloroethane (10 mL), the mixture of freshly distilled DMF (5.0 mL) and $POCl_3$ (1.0 mL) was added dropwise under N_2 atmosphere at 0 °C. After addition, the cooling bath was removed and the reaction mixture was refluxed at 80 °C for 4 h. The mixture was quenched with saturated aqueous solution of sodium acetate and was stirred for another 1 h and the reaction mixture was then extracted with dichloromethane. The organic layers were combined and washed with saturated brine solution and dried over anhydrous Na_2SO_4. After removal of the solvent, the crude product was purified by silica gel column chromatography, eluting with petroleum ether/dichloromethane (1:1) to obtain the product as orange solid (0.51 g, 96.9%). ^{1}H NMR (400 MHz, Chloroform-*d*): δ 10.14 (s, 1H), 10.12 (s, 1H), 4.69-4.58 (m, 4H), 3.20 (t, *J* = 7.7 Hz, 2H), 3.11 (d, *J* = 7.5 Hz, 2H), 2.11-2.05 (m, 1H), 2.04-1.98 (m, 2H), 1.96-1.88 (m, 2H), 1.71-1.63 (m, 5H), 1.37-1.23 (m, 30H), 1.17-1.03 (m, 10H), 0.91-0.83 (m, 20H), 0.70-0.64 (m, 6H), 0.62-0.57 (m, 6H). ^{13}C NMR (101 MHz, Chloroform-*d*): δ 181.99, 181.79, 147.54, 146.87, 146.44, 143.40, 143.15, 137.77, 137.03, 136.81, 132.73, 129.64, 129.49, 127.23, 112.40, 112.37, 55.16, 40.12, 39.20, 37.69, 37.56, 33.86, 33.54, 33.47, 33.24, 31.82, 30.34,

29.98, 29.67, 29.64, 29.61, 29.53, 29.37, 28.82, 28.16, 27.55, 27.49, 26.88, 26.78, 26.60, 26.47, 23.11, 23.08, 22.99, 22.70, 22.68, 22.64, 14.09, 14.06, 13.65, 10.14, 10.06.

Synthesis of compound A-C10ch

In a 100 mL two-neck round-bottom flask, compound **12** (100 mg, 0.09 mmol) and 2-(5, 6-difluoro-3-oxo-2,3-dihydro-1*H*-inden-1-ylidene)malononitrile (165 mg, 0.72 mmol) were added. The reaction mixture was evacuated and backfilled with N_2 three times, and then freshly degassed chloroform (20 mL) and pyridine (0.5 mL) were added the reaction mixture was stirred at room temperature for 12 h. Then the mixture was poured into methanol and the precipitate was filtered off and washed with methanol. The crude product was purified by silica gel column chromatography, eluting with petroleum ether/dichloromethane (1:2) to give the product as blue-black solid (98 mg, 70.1%). ^{1}H NMR (400 MHz, Chloroform-d): δ 9.07-8.95 (m, 2H), 8.49-8.36 (m, 2H), 7.67-7.55 (m, 2H), 4.82-4.63 (m, 4H), 3.17-2.98 (m, 4H), 2.11-1.95 (m, 3H), 1.83-1.72 (m, 2H), 1.63-1.52 (m, 5H), 1.42-0.88 (m, 52H), 0.82-0.67 (m, 14H), 0.66-0.53 (m, 6H). ^{13}C NMR (101 MHz, Chloroform-*d*): δ 152.50, 146.50, 144.32, 144.12, 136.72, 134.72, 133.03, 132.86, 132.81, 132.19, 114.09, 113.53, 39.40, 39.02, 36.66, 36.54, 32.58, 32.44, 32.32, 30.80, 30.16, 28.98, 28.79, 28.67, 28.63, 28.60, 28.50, 28.43, 27.83, 26.66, 25.87, 25.76, 25.57, 25.44, 22.26, 21.96, 21.81, 21.62, 13.05, 13.02, 12.72, 9.23.

2. Measurements and characterization.

Nuclear magnetic resonance (NMR): 1H and 13C NMR were measured on a Bruker AV-500 MHz spectrometer in deuterated solvents at room temperature. Chemical shifts were recorded with tetramethylsilane (TMS) as the internal reference.

UV-vis absorption spectra: The UV–vis absorption spectra of solutions and films were recorded using a Hitachi U-4100 spectrophotometer.

Cyclic voltammetry (CV): Cyclic voltammetry measurements were carried out on a CHI660A electrochemical workstation with a three-electrode configuration, using Ag/AgCl as the reference electrode, a platinum plate as the counter electrode, and a glassy carbon as the working electrode. Tetrabutylammonium hexafluorophosphate in anhydrous acetonitrile (0.1 mol L^{-1}) was used as the supporting electrolyte. A ferrocene/ferrocenium (Fc/Fc^{+}) redox couple was used as internal standard and was assigned an absolute energy of – 4.8 eV versus vacuum. The HOMO and LUMO energy levels of the materials were determined according to the equation $E_{HOMO/LUMO} = -(E_{ox\ onset}/E_{red\ onset} - E_{1/2}^{(Fc/\ Fc+)} + 4.80)$.

Ultraviolet Photoelectron Spectroscopy Measurement: Ultraviolet photoelectron spectrometer (UPS) measurement was performed by AXIS ULTRA DLD instrument of Kratos company. The UV light source used is non-monochromatic He I, and the energy of He I light source is 21.22 eV. The basic vacuum of the analysis chamber is 3.0×10^{-8} Torr, and the bias voltage applied during the test is −9 V.

Atomic force microscopy (AFM): Standard tapping-mode AFM measurements in ambient were performed on a Scanned Probe Imaging and Development (SPID) on Park NX-10. The AFM images were confirmed from different samples and scan areas. The root-mean-square roughness (RMS) values of height images were obtained from the whole scan area (5 μm × 5 μm). All the AFM images were flattened and exported from the software.

Transmission electron microscope (TEM): Transmission electron microscopy (TEM) measurement was performed by using a HITACHI H-7650 electron microscope with an

acceleration voltage of 110 kV.

GIWAXS and GISAXS characterization: Grazing incidence wide-angle X-ray scattering (GIWAXS) and grazing incidence small-angle X-ray scattering (GISAXS) patterns were acquired by beamline BL16B1 at Shanghai Synchrotron Radiation Facility (SSRF).[1] The X-ray wavelength was chosen as 0.124 nm (E = 10 keV), and the incidence angle was set to 0.15 degree. For GIWAXS and GISAXS experiments, the sample to detector distance was 276.26 mm and 5520.51 mm.

Single Crystal X-Ray Diffraction: Single crystals data collection of Y-C10ch, A-C10ch were performed at 193 K on a Bruker D8 Venture. Single crystals data of L8-BO are from CCDC No. 2005533.[2] All calculations were performed using the SHELXL and the crystallographic software package.

3. Solar cell device fabrication and characterization: OSCs devices were fabricated with a conventional device structure of ITO/PEDOT:PSS/active layer/PNDIT-F3N/Ag. The ITO-coated glass substrates were sonicated successively with detergent, deionized water, acetone and isopropanol, and dried with nitrogen flow. Immediately prior to device fabrication, the substrates were cleaned by oxygen plasma for 10 min. And then, the PEDOT:PSS layer was spin-coated onto the ITO and annealed at 150 °C for 15 min. The PM6/L8-BO and PM6/Y-C10ch and PM6/A-C10ch solutions were stirred at room temperature before spin-coating. And then, PM6/Acceptor (different weight ratio) solution with 0.25% DIM was spin-coated on the top of PEDOT:PSS from the chloroform (CF) solution. Then PNDIT-F3N solution (methanol:acetic acid, 200:1) was spin-coated as electron transfer layer. Finally, silver (100

nm) was evaporated onto the active layer at a vacuum of ~2×10^{-4} Pa to form the top electrode.

Characterization of solar cells: The current-voltage (J-V) characteristics were measured with a Keithley 2450 source measurement unit. The OSCs were measured under an irradiation intensity of 100 mW/cm^2 (AM 1.5 G) by a Newport solar simulator.

External quantum efficiencies (EQEs): The EQE spectra were analyzed using an integrated system (LST-QE).

The High Sensitive EQE measurements: The High Sensitive EQE was measured by using an integrated system (LST-QE), where the photocurrent was amplified and modulated by a lock-in instrument.[3]

Electroluminescence (EL) measurements: Electroluminescence (EL) quantum efficiency (EQE_{EL}) measurements were performed by applying external voltage/current sources through the devices (LST-QE).

Fabrication and characterization of single-carrier devices: The hole and electron mobilities were calculated using the space charge limited current (SCLC) model with a device configuration of ITO/PEDOT:PSS/active layer/MoO_3/Ag and ITO/ZnO/active layer/ PNDIT-F3N/Ag, respectively, where the current density is calculated by:

$$J = 9\varepsilon_o\varepsilon_r\mu V^2/(8L^3)$$

where J stands for current density, ε_o is the permittivity of free space, ε_r is the relative dielectric constant of the transport medium, μ is the hole mobility, V is the voltage drop across the device ($V = V_{appl} - V_{bi} - V_{RS}$, where V_{appl} is the applied voltage to the device, V_{bi} is the built-in voltage due to the difference in work function of the two electrodes, and V_{RS} is the voltage drop due to series resistance across the electrodes), and L is the thickness of the active

layer.

Femtosecond Transient Absorption Spectroscopy Measurements: Femtosecond transient absorption spectroscopy measurements were performed on an Ultrafast Helios pump-probe system in collaboration with a regenerative amplified laser system from Coherent. An 800 nm pulse with a repetition rate of 1k Hz, a length of 100 fs, and an energy of 10 mJ pulse, was generated by an Ti:sapphire amplifier (Astrella, Coherent). Then the 800 nm pulse was separated into two parts by a beam splitter. One part was coupled into an optical parametric amplifier (TOPAS, Coherent) to generate the pump pulses at various wavelength. The other part was focused onto a sapphire plate and a YAG plate to generate white light supercontinuum as the probe beams with spectra covering 420-800 nm and 750-1400 nm, respectively. The time delay between pump and probe was controlled by a motorized optical delay line with a maximum delay time of 8 ns. The pump pulse is chopped by a mechanical chopper with 500 Hz and then focused on to the mounted sample with probe beams. The probe beam was collimated and focused into a fiber-coupled multichannel spectrometer with CCD sensor. The energy of pump pulse was measured and calibrated by a power meter (PM400, Thorlabs). The samples used for TA measurements were obtained by spin-coating the neat and blend solutions on the quartz substrates. The detailed calculation processes of exciton diffusion length were reported in previous literature.[4]

4. Additional figures and tables.

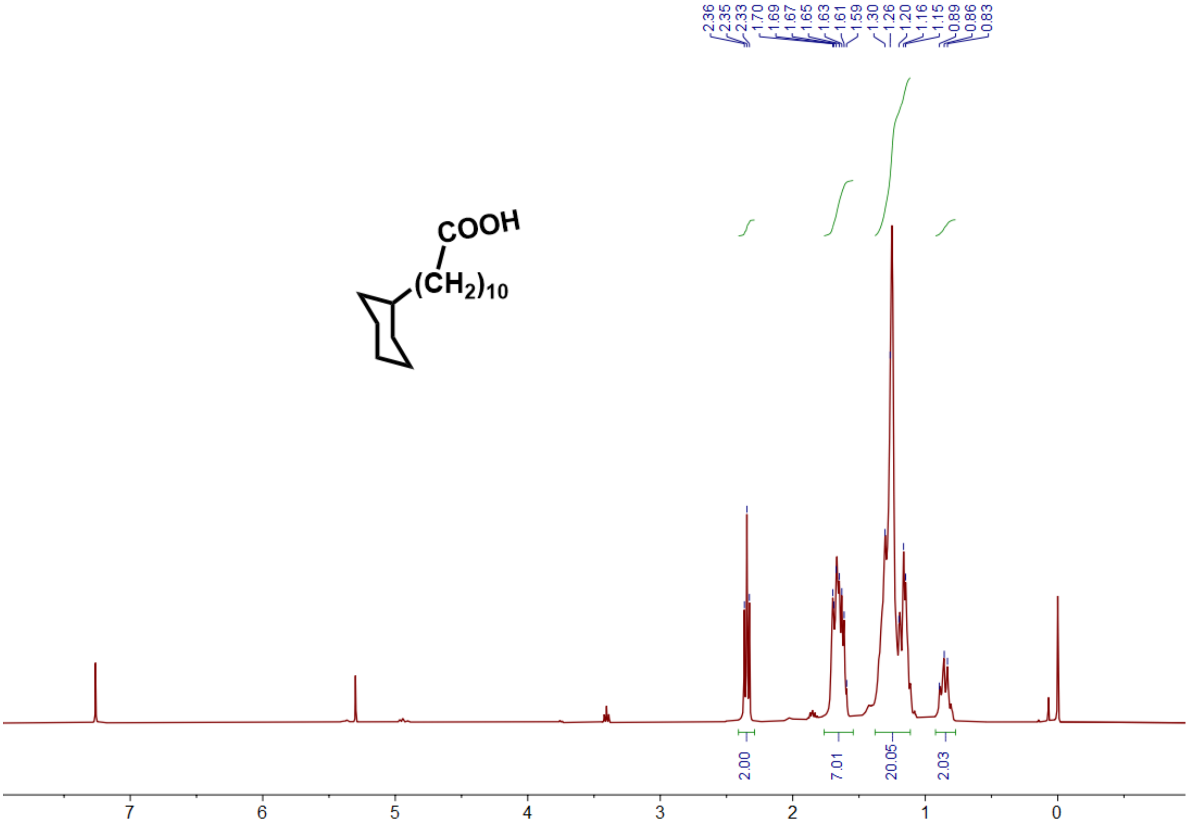

Figure S1. ^{1}H NMR spectrum of 1.

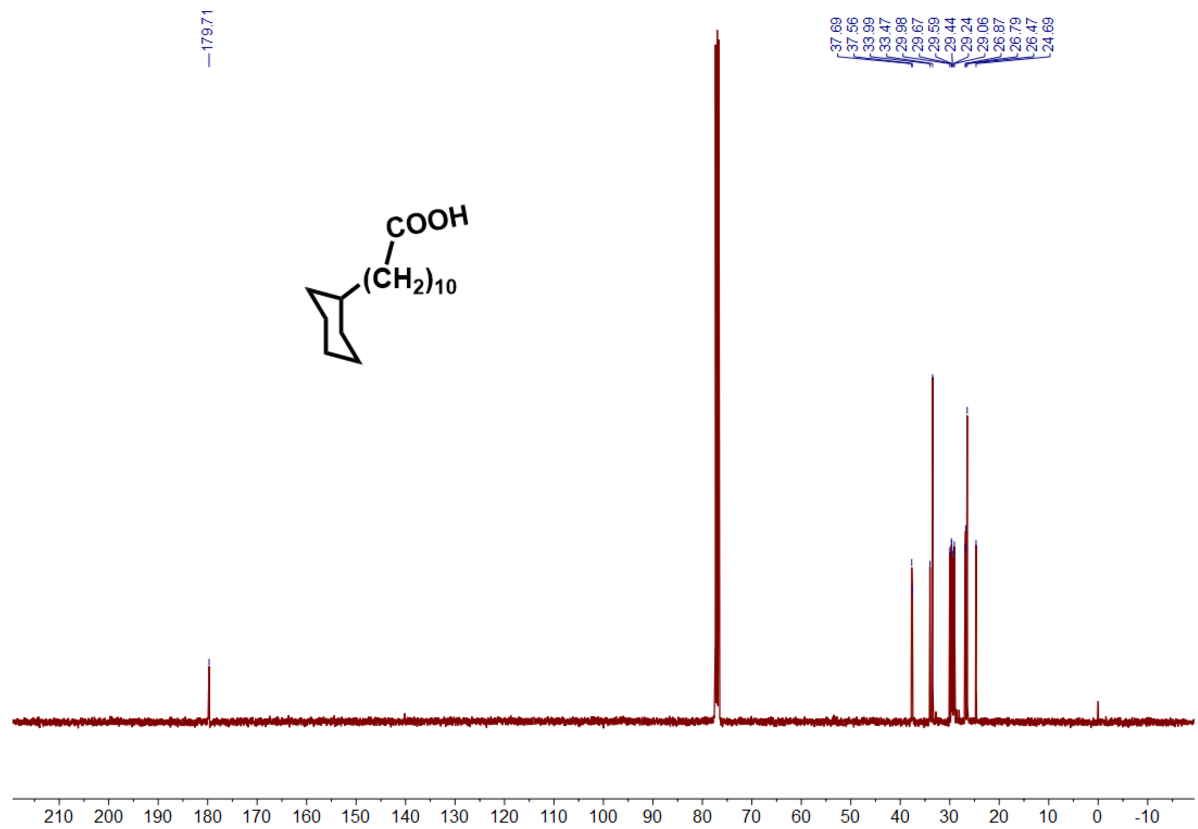

Figure S2. ^{13}C NMR spectrum of 1.

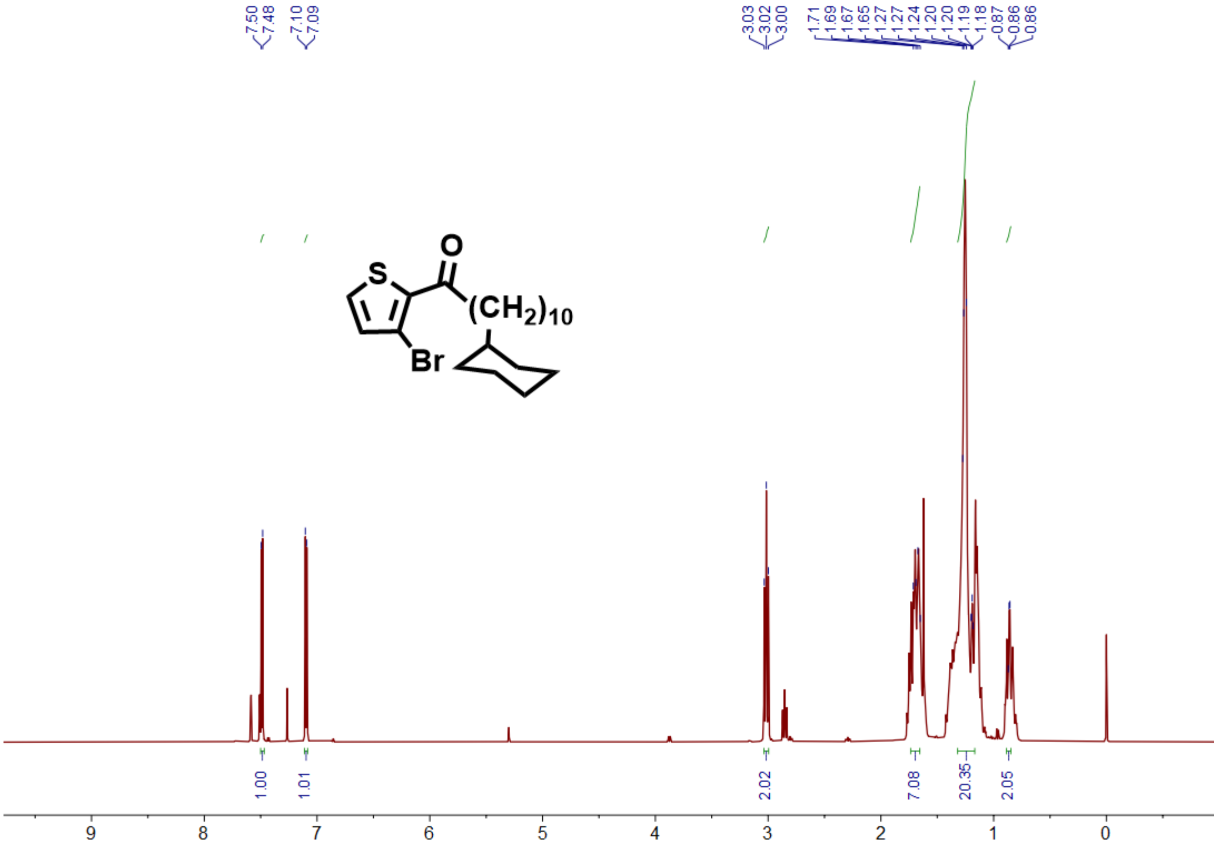

Figure S3. ^{1}H NMR spectrum of 2.

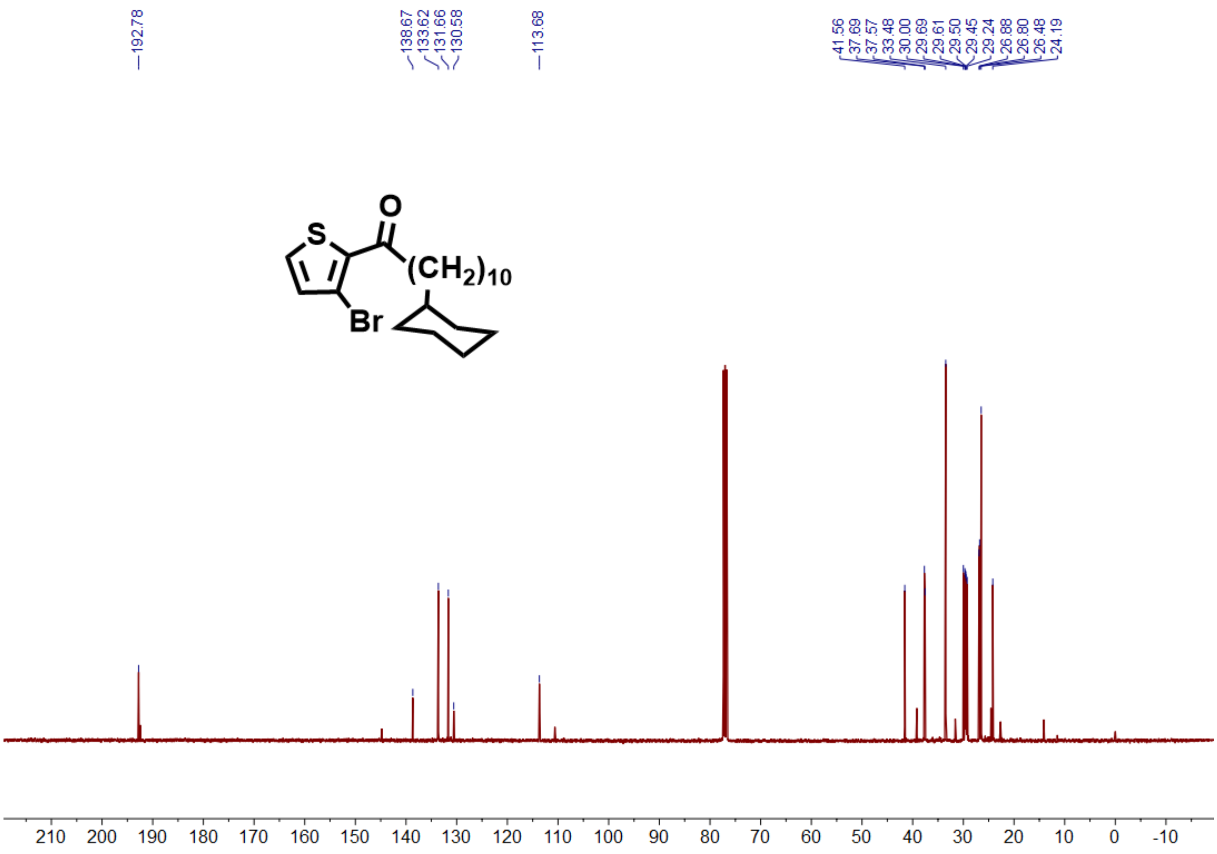

Figure S4. ^{13}C NMR spectrum of 2.

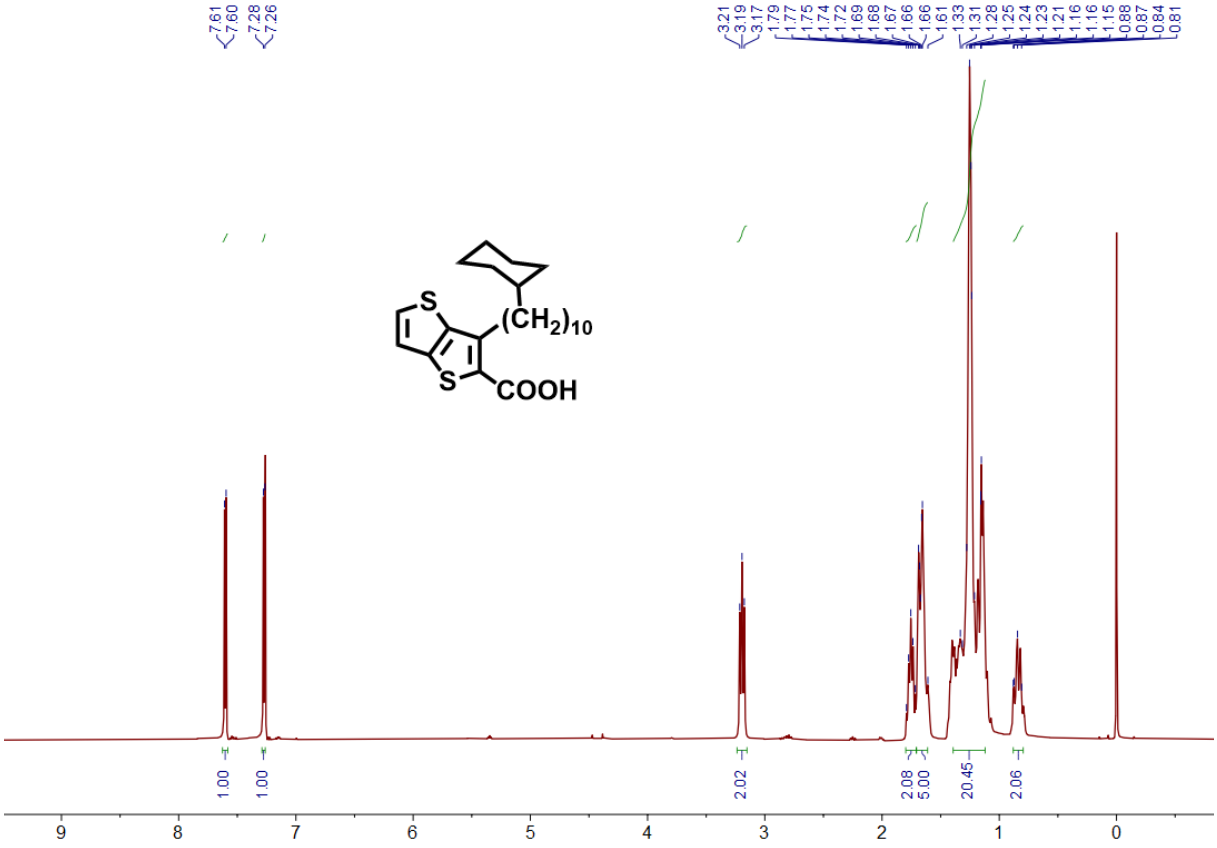

Figure S5. ^{1}H NMR spectrum of 3.

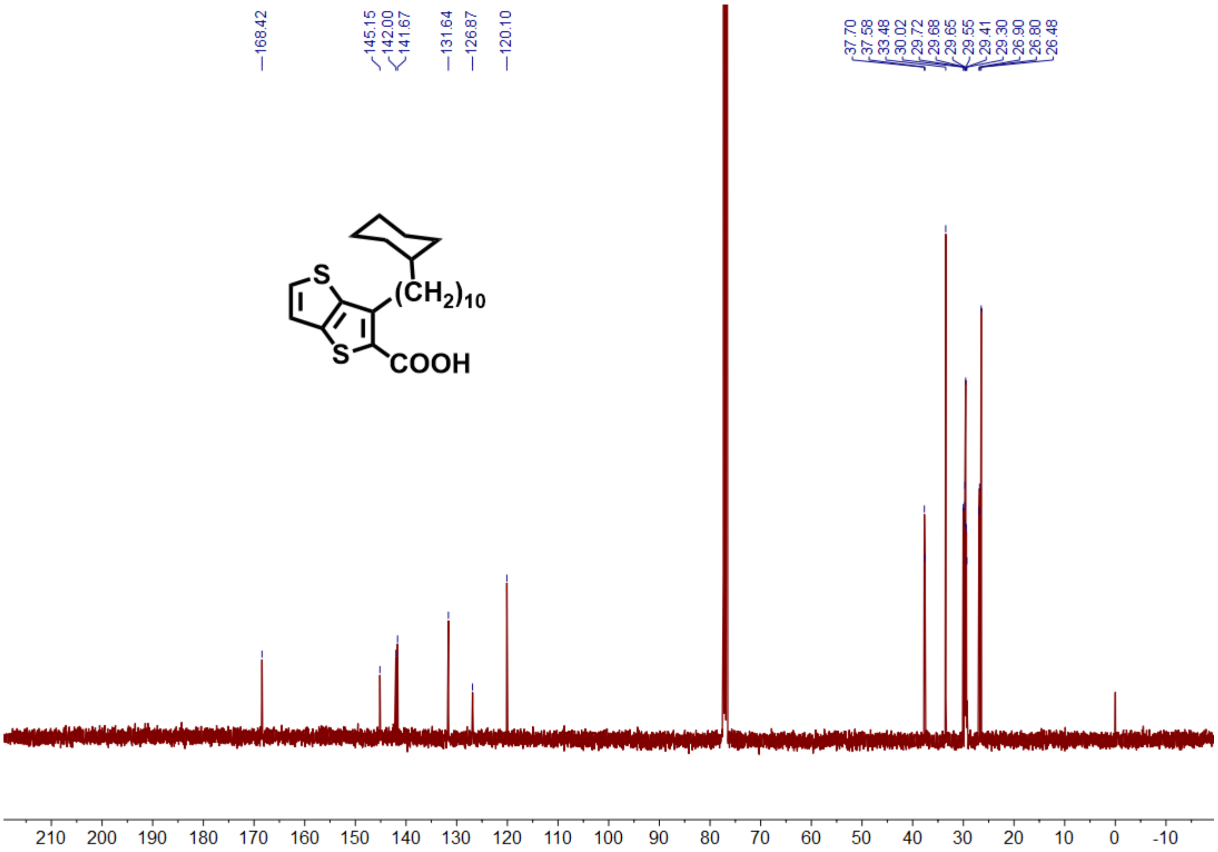

Figure S6. ^{13}C NMR spectrum of 3.

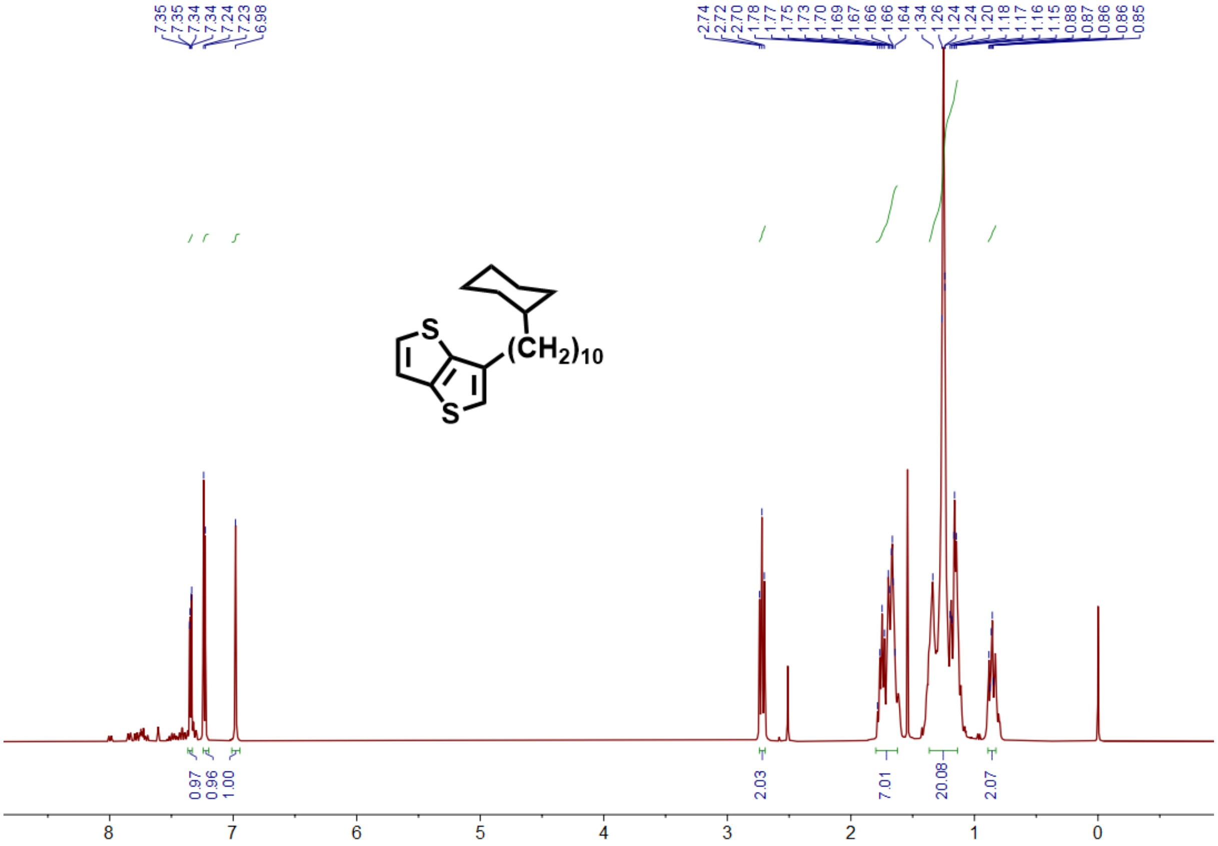

Figure S7. ^{1}H NMR spectrum of 4.

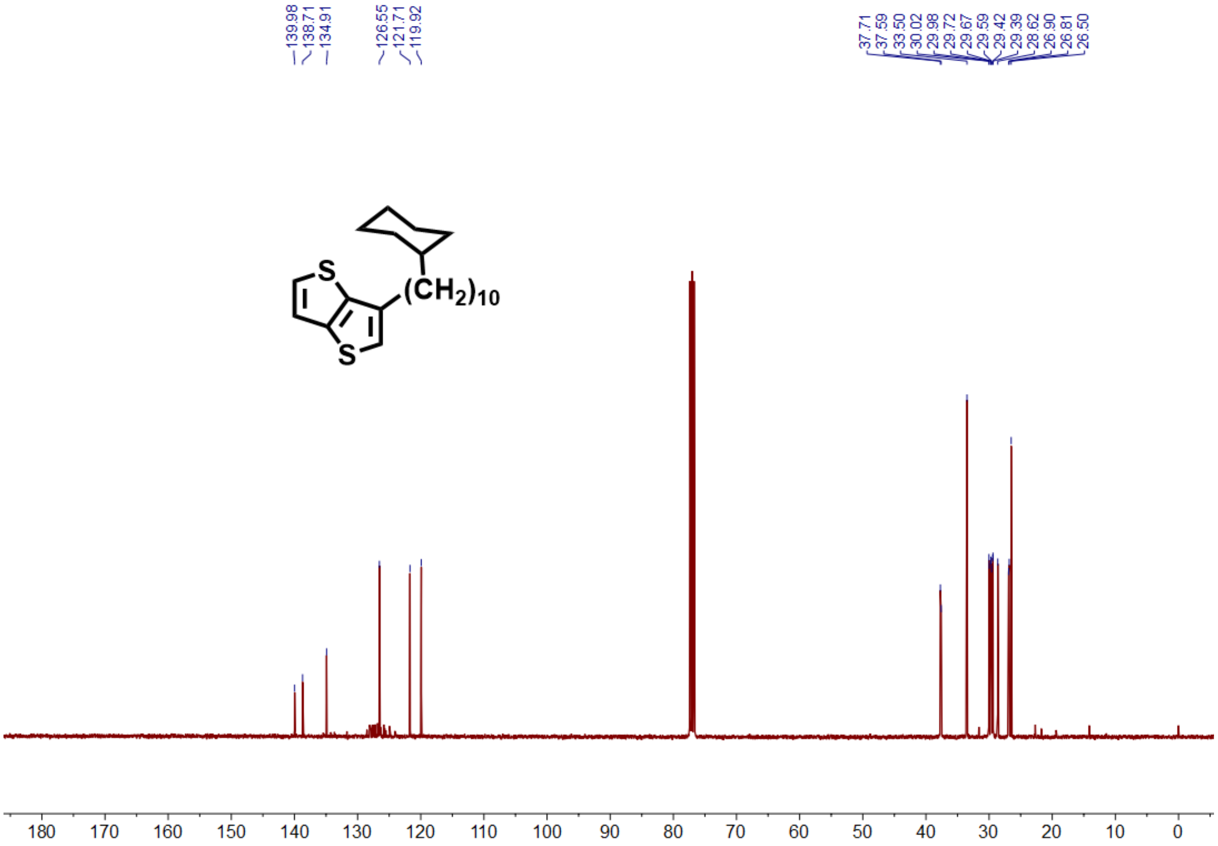

Figure S8. ^{13}C NMR spectrum of 4.

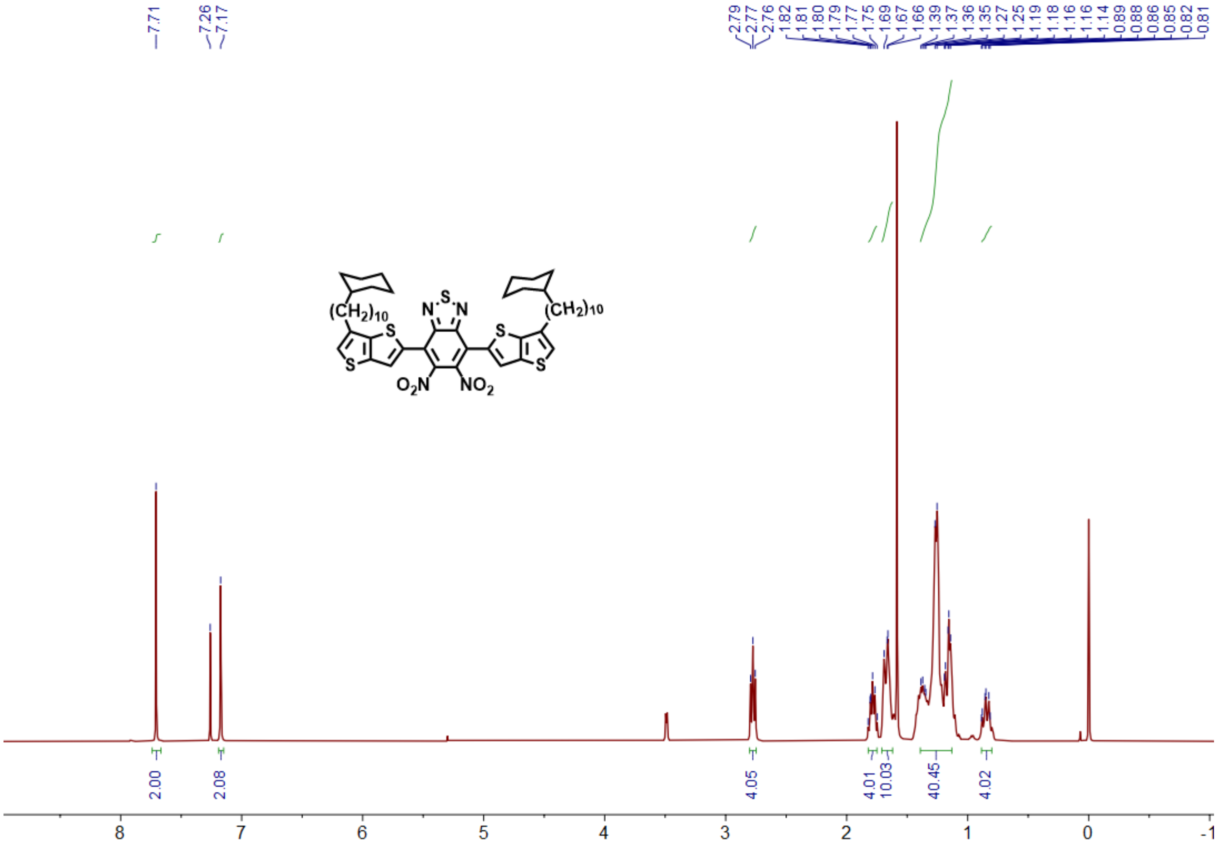

Figure S9. ^{1}H NMR spectrum of 6.

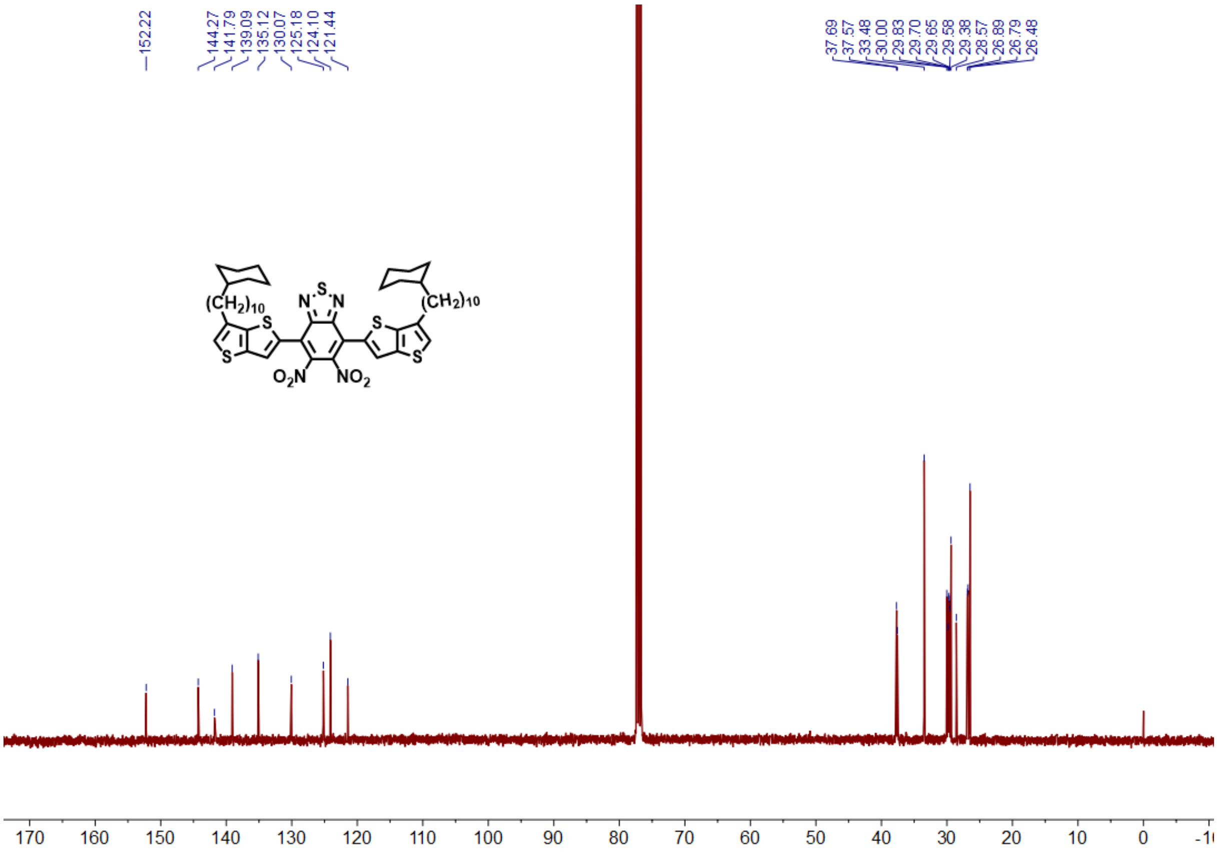

Figure S10. ^{13}C NMR spectrum of 6.

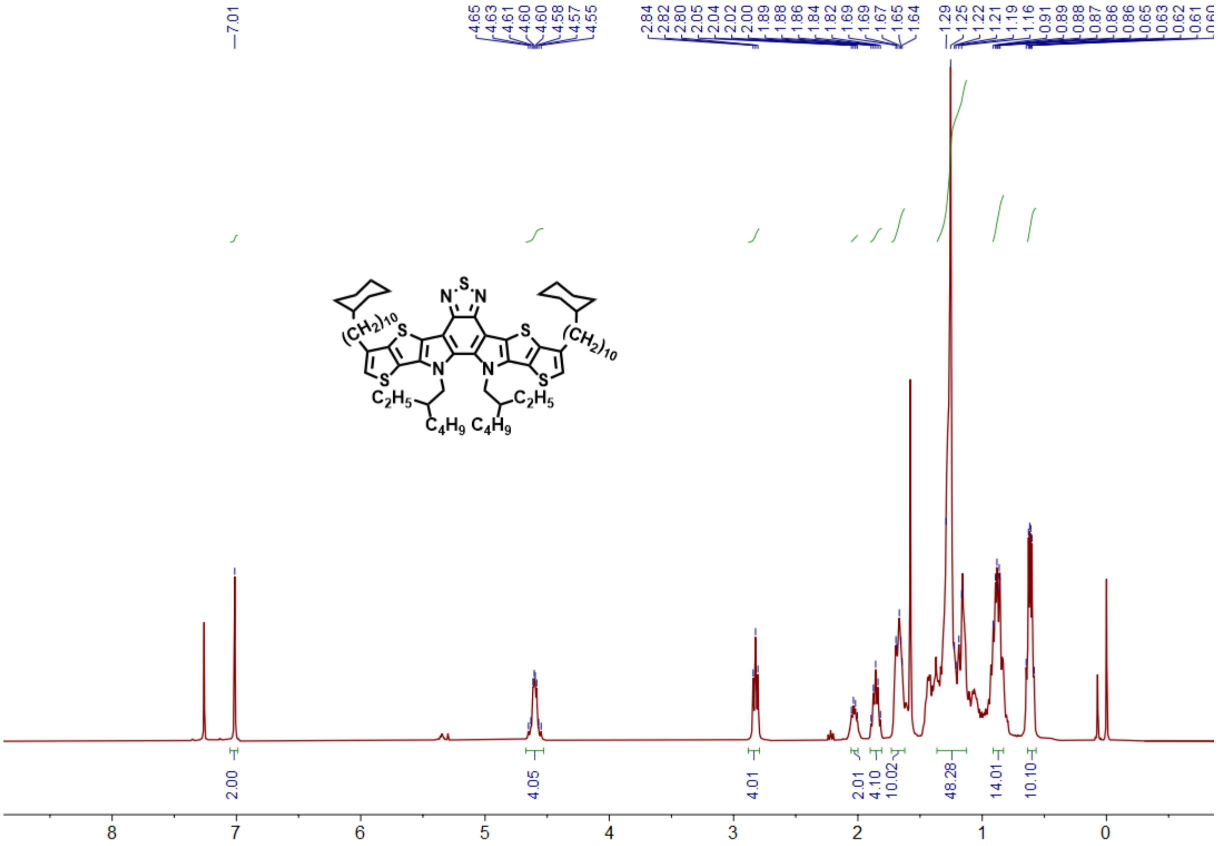

Figure S11. ^{1}H NMR spectrum of 7.

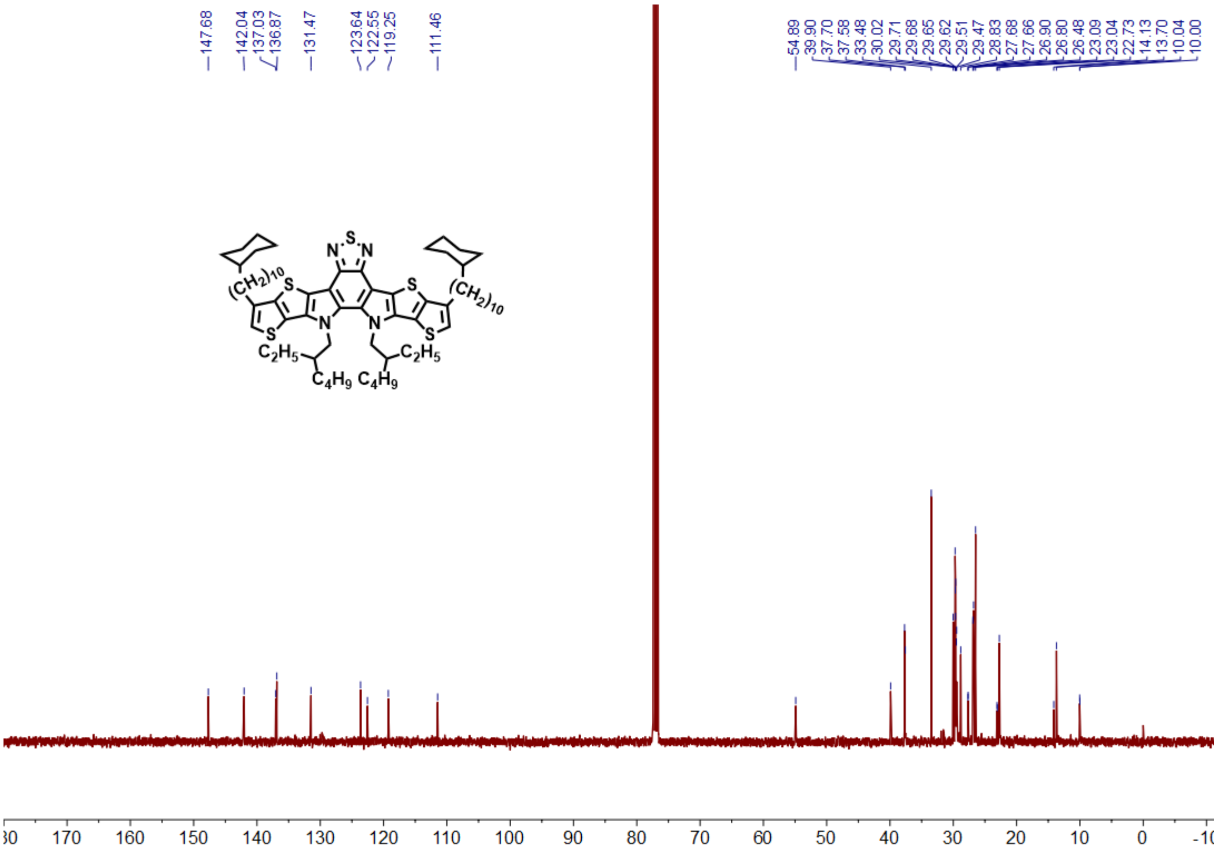

Figure S12. ^{13}C NMR spectrum of 7.

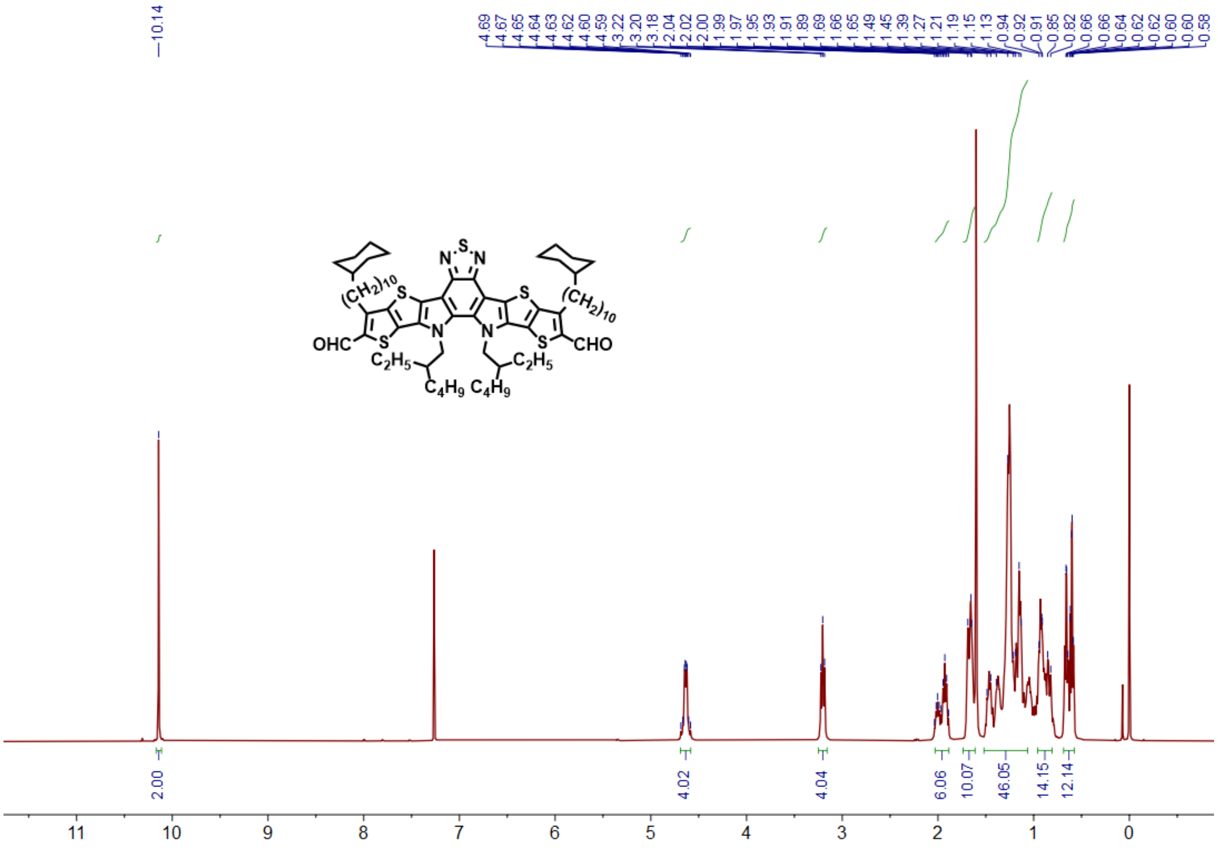

Figure S13. ^{1}H NMR spectrum of 8.

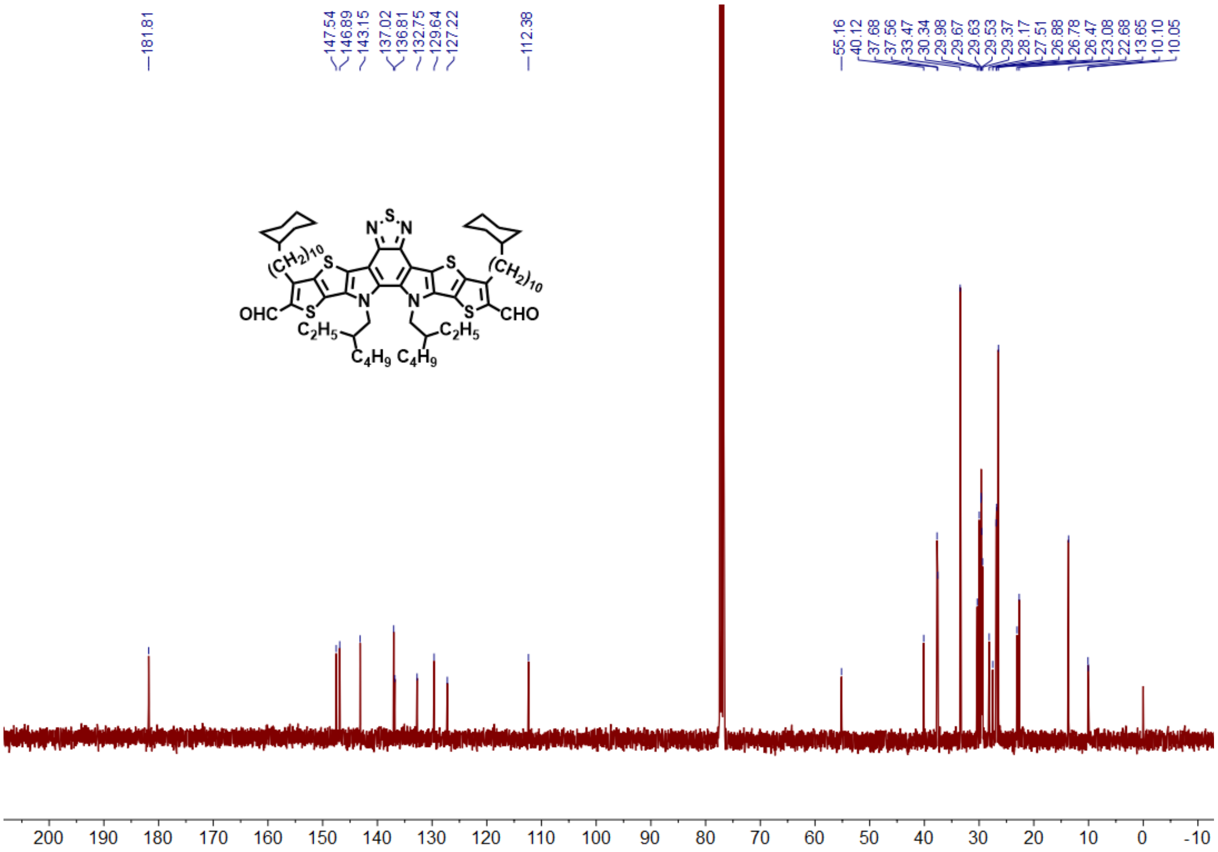

Figure S14. ^{13}C NMR spectrum of 8.

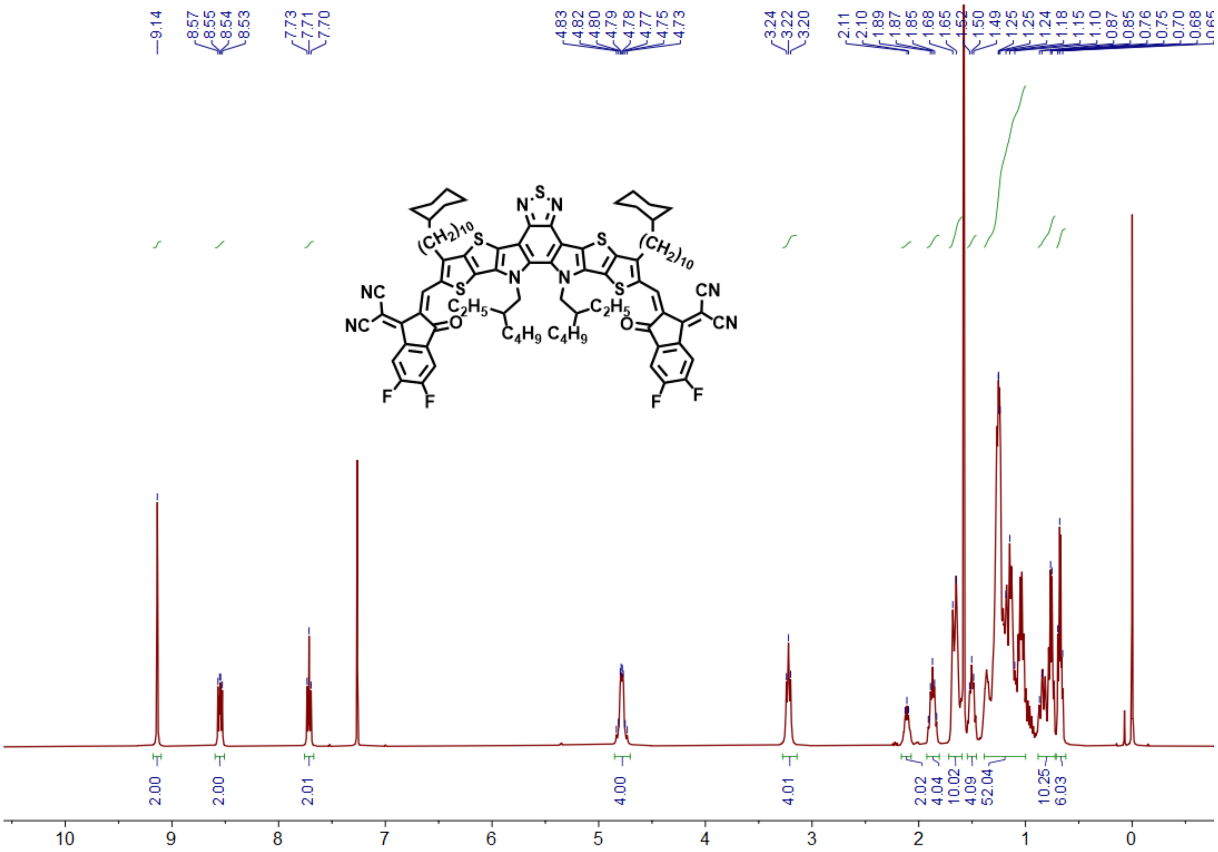

Figure S15. ^{1}H NMR spectrum of Y-C10ch.

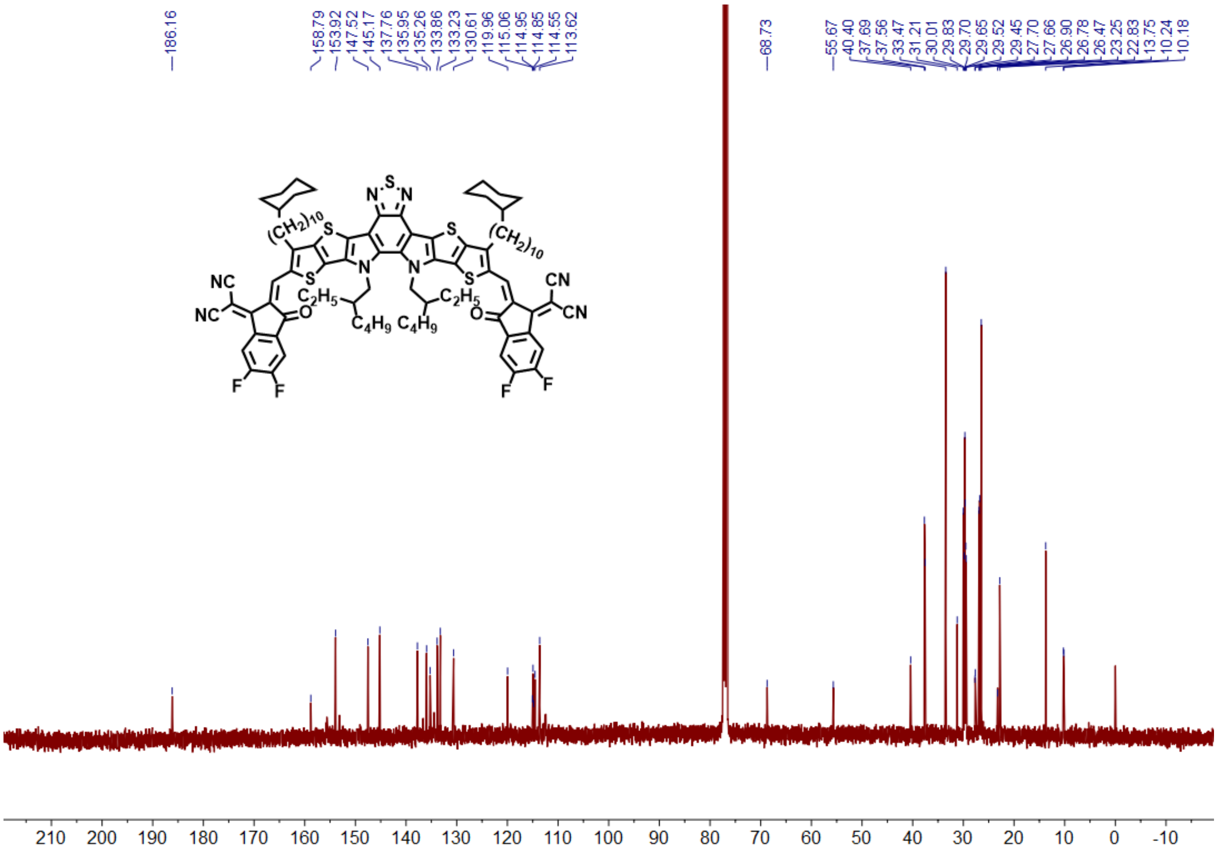

Figure S16. ^{13}C NMR spectrum of Y-C10ch.

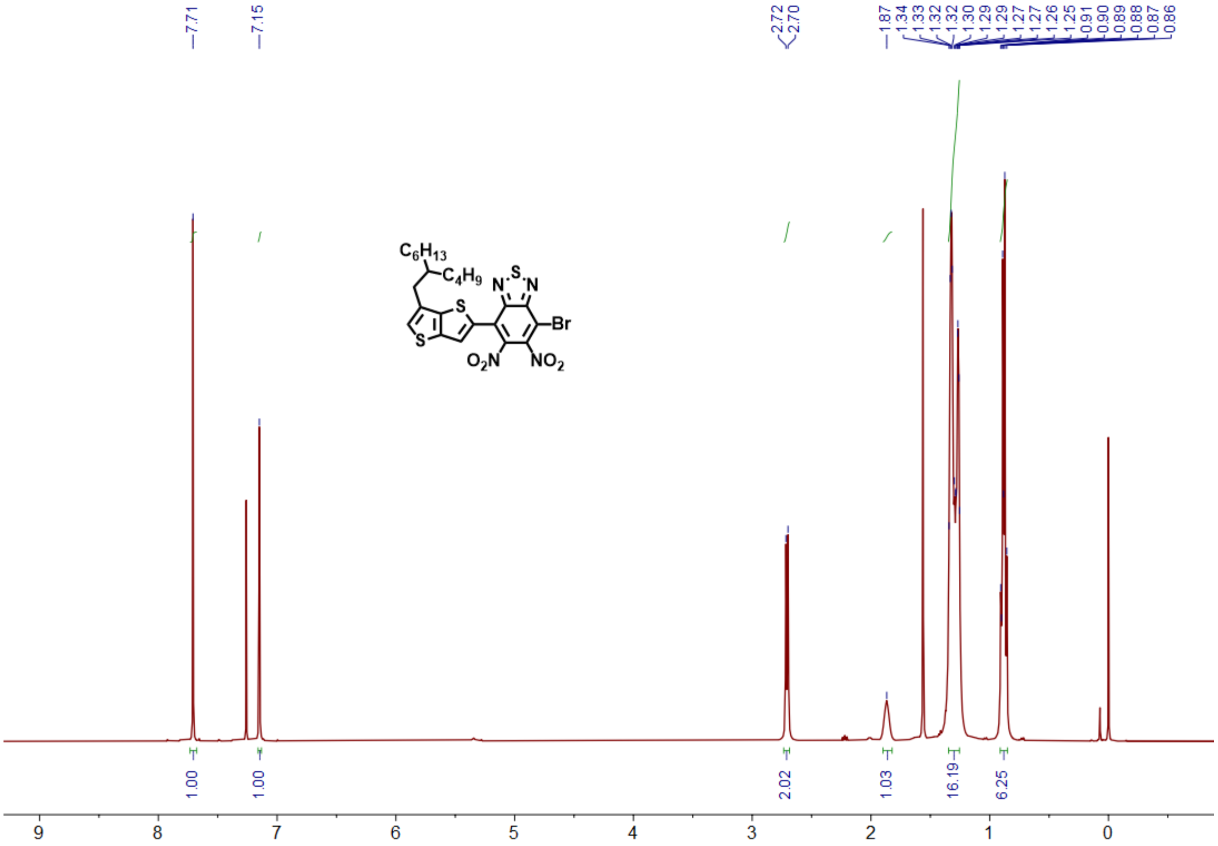

Figure S17. ^{1}H NMR spectrum of 9.

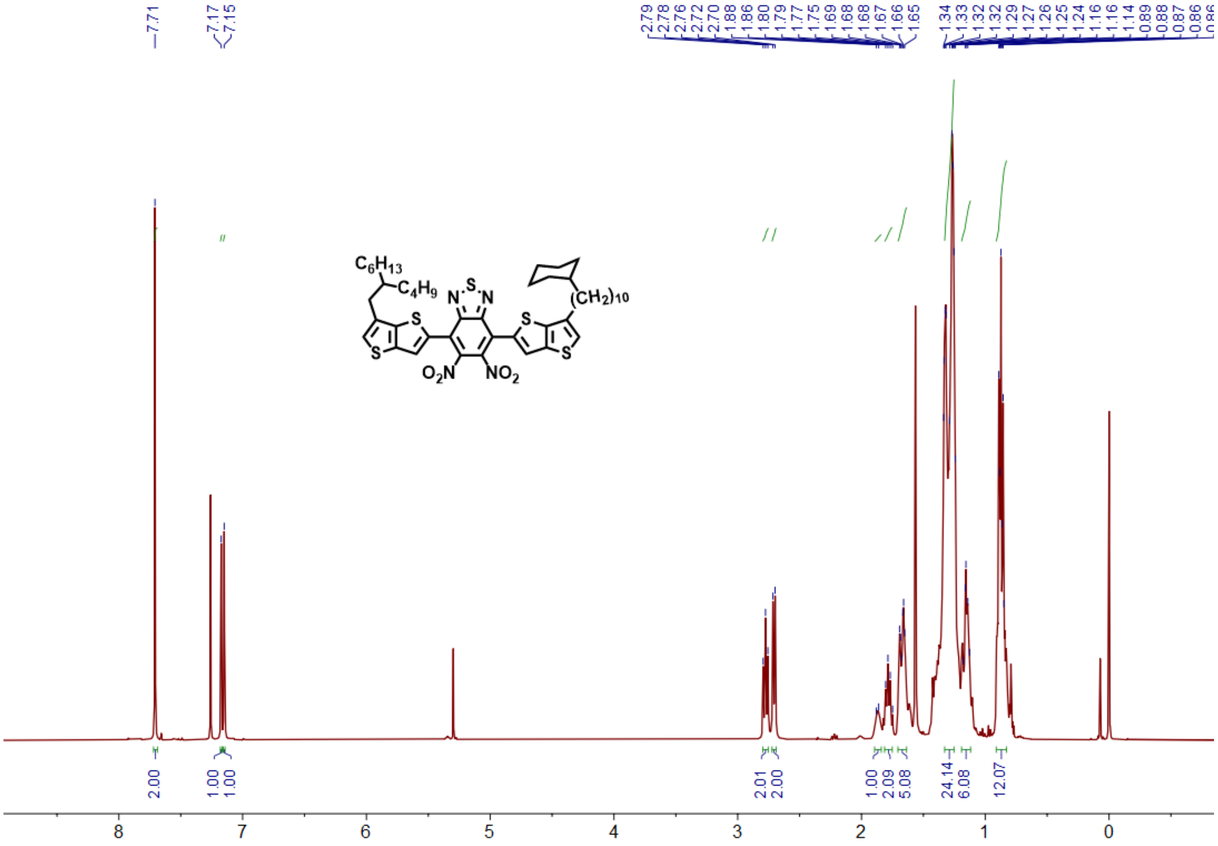

Figure S18. ^{1}H NMR spectrum of 10.

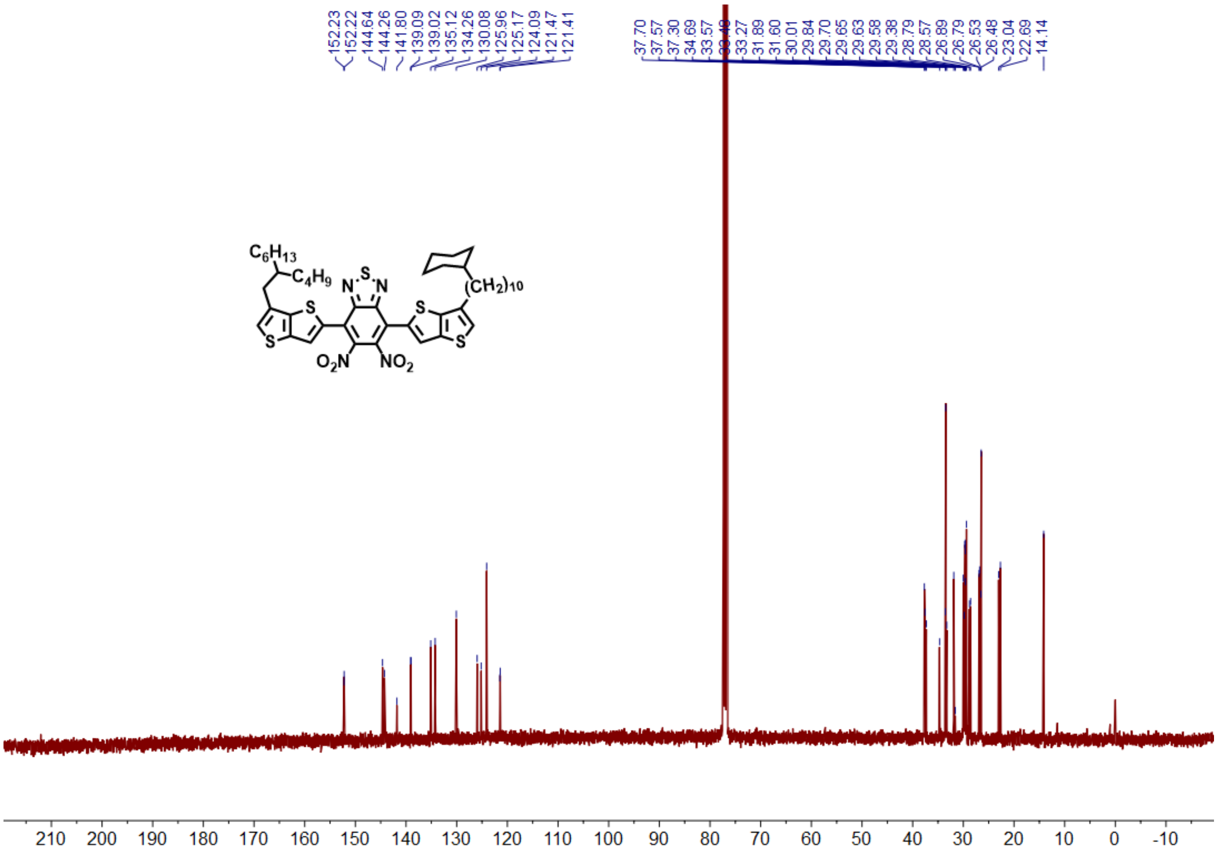

Figure S19. ^{13}C NMR spectrum of 10.

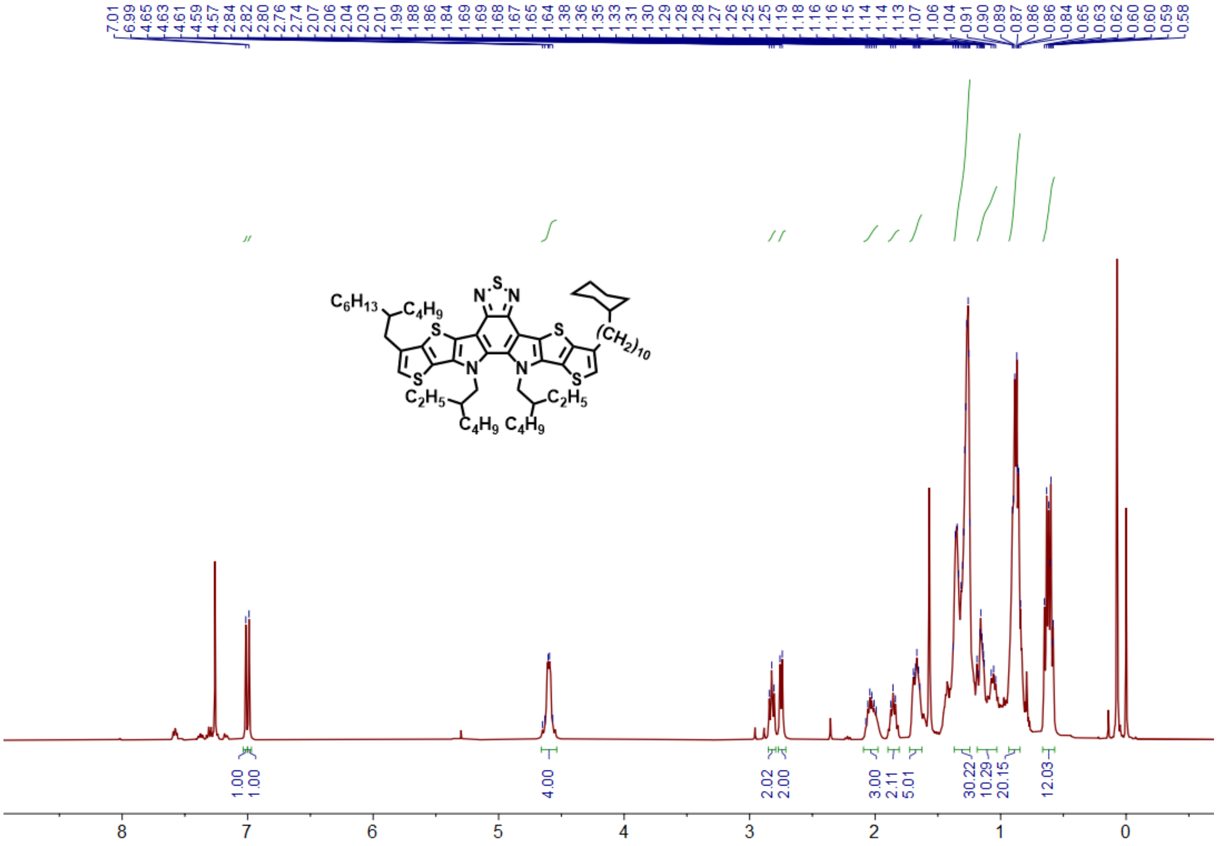

Figure S20. ^{1}H NMR spectrum of 11.

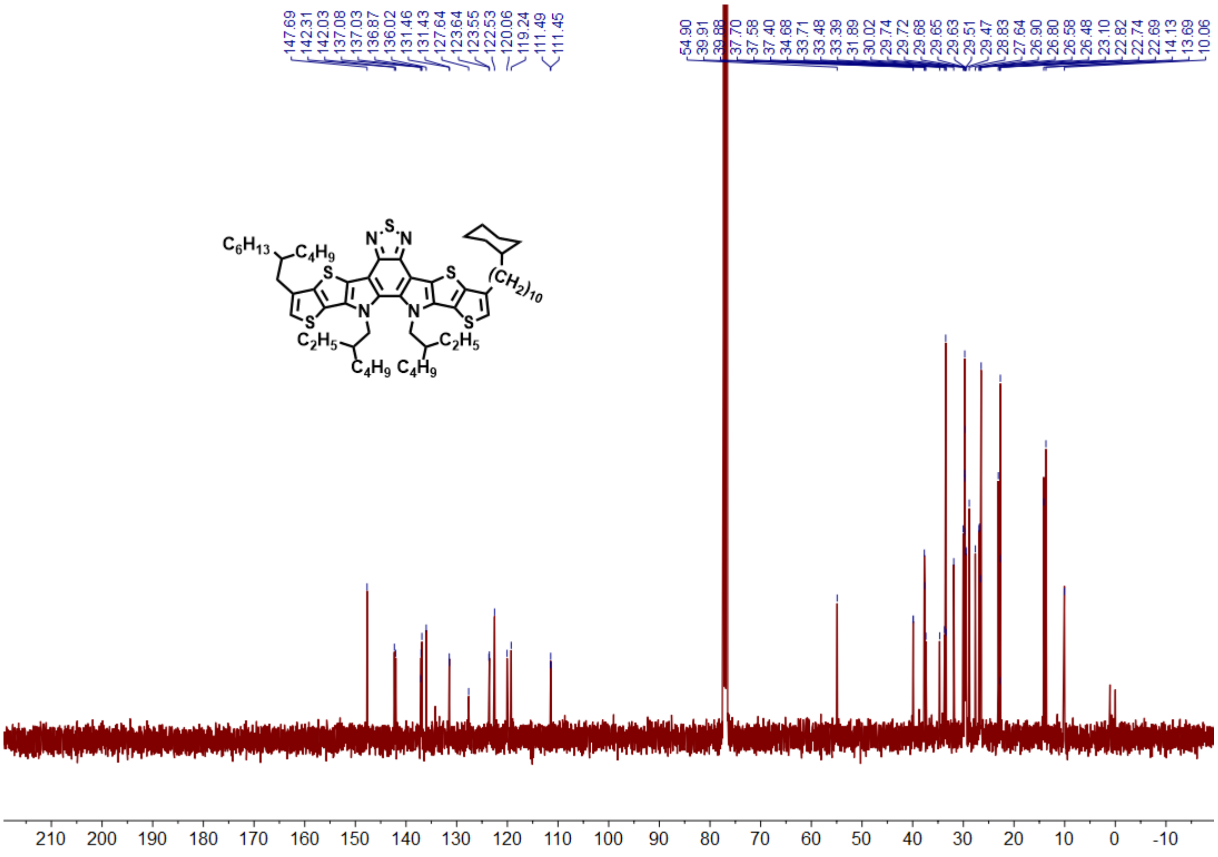

Figure S21. ^{13}C NMR spectrum of 11.

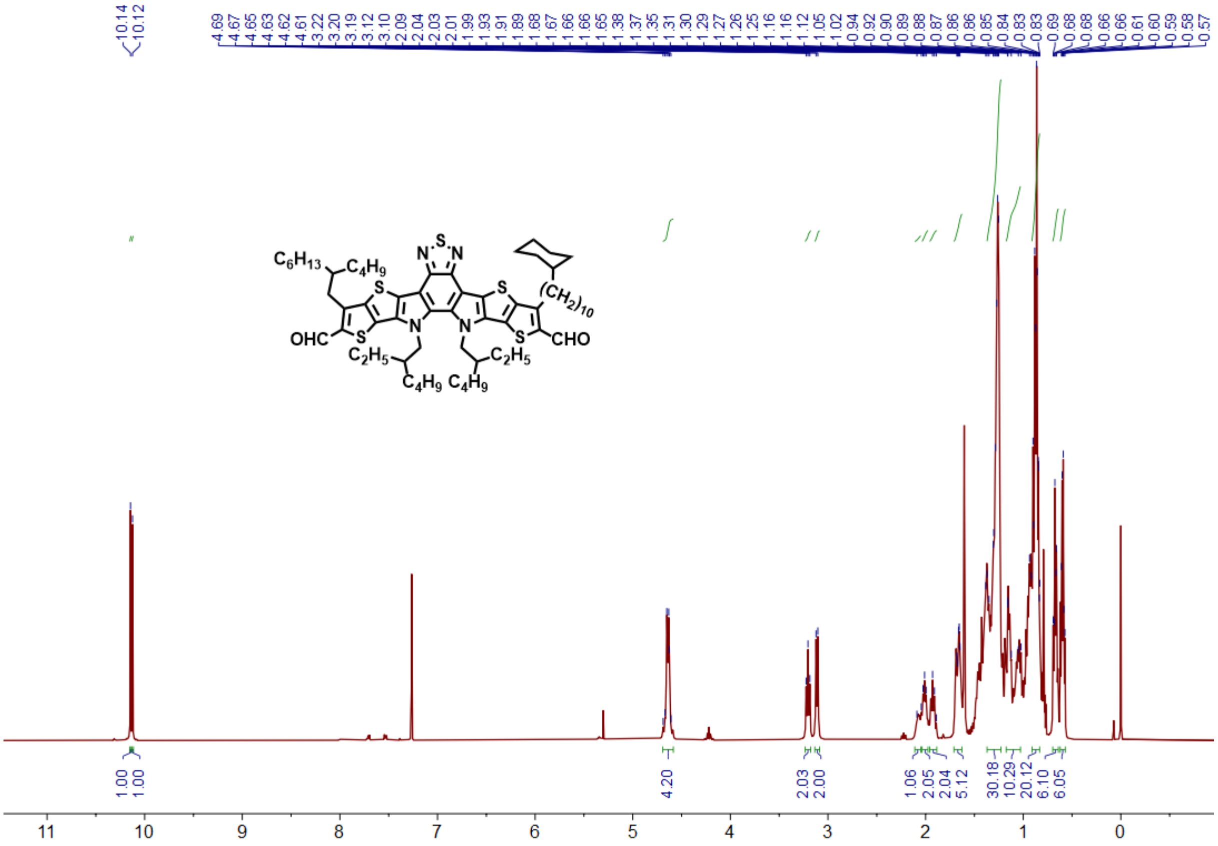

Figure S22. ^{1}H NMR spectrum of 12.

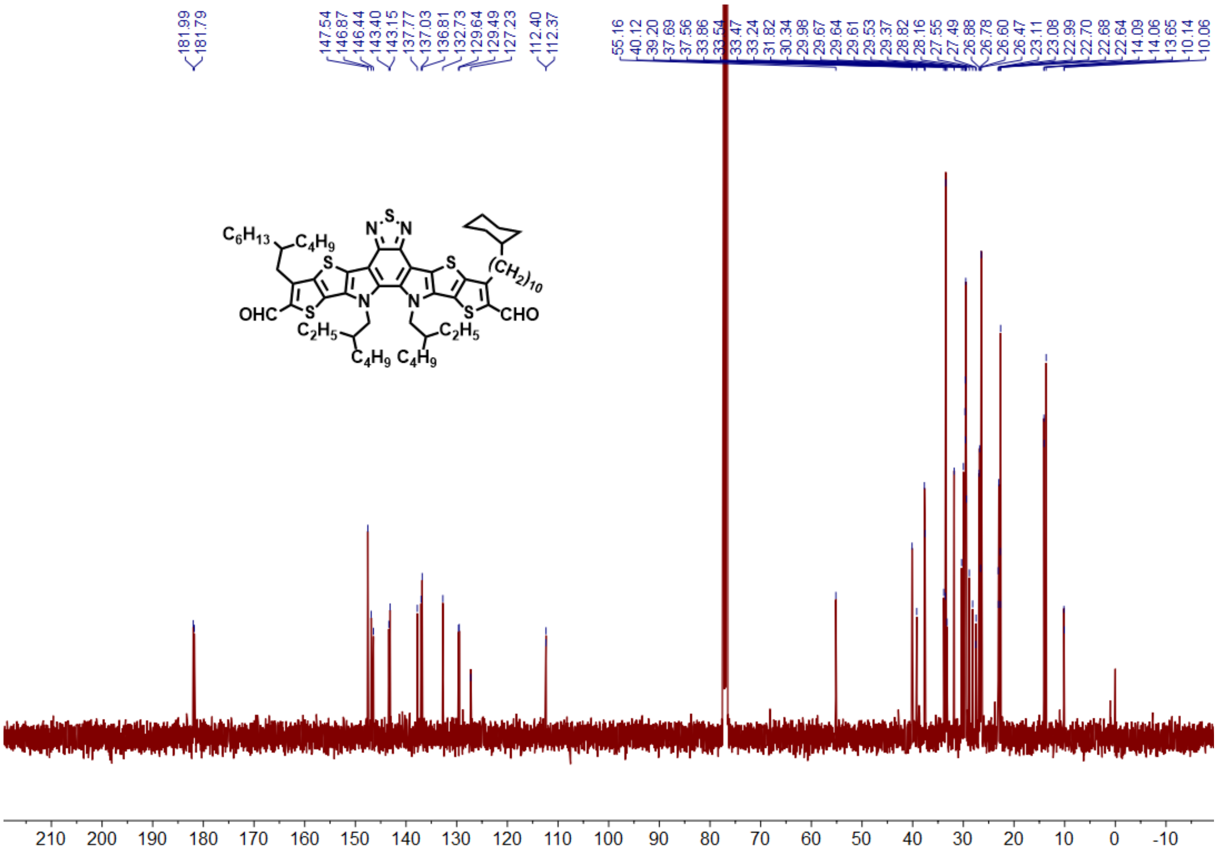

Figure S23. ^{13}C NMR spectrum of 12.

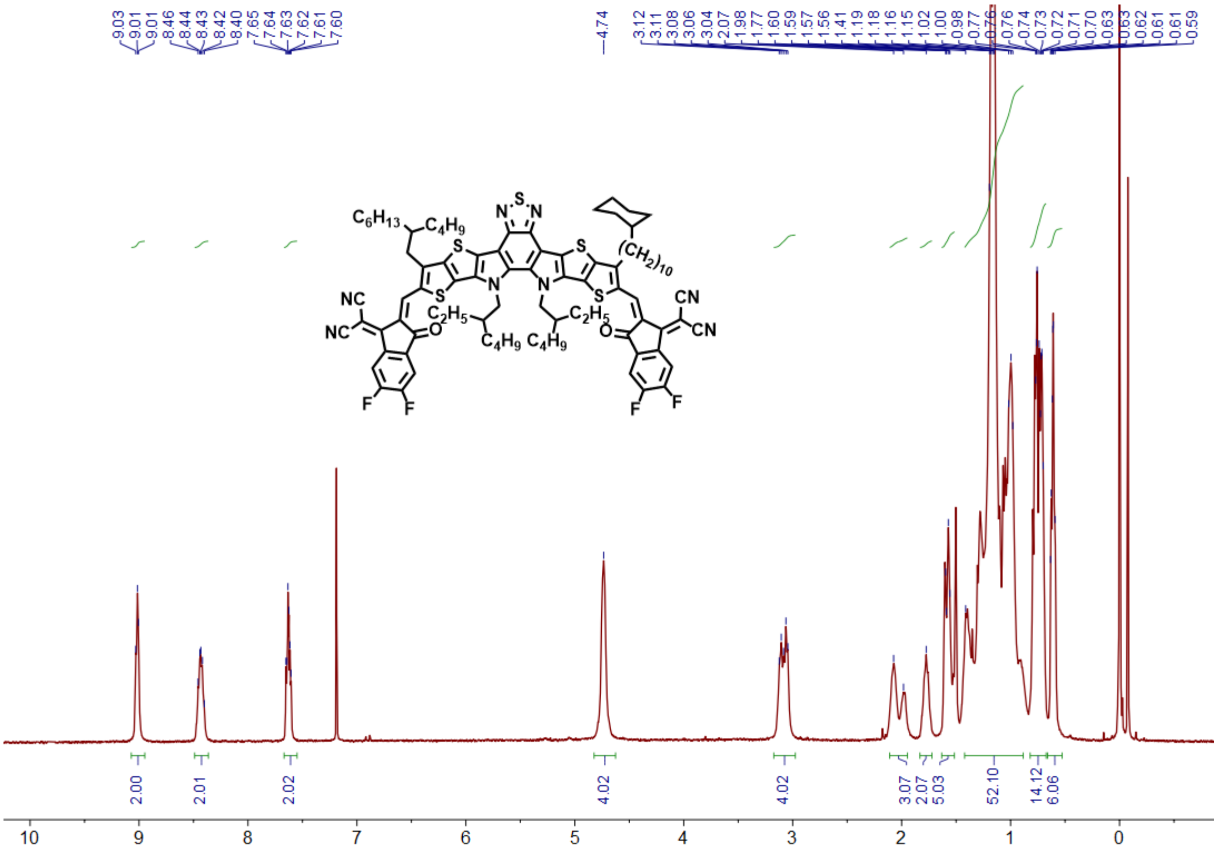

Figure S24. ^{1}H NMR spectrum of A-C10ch.

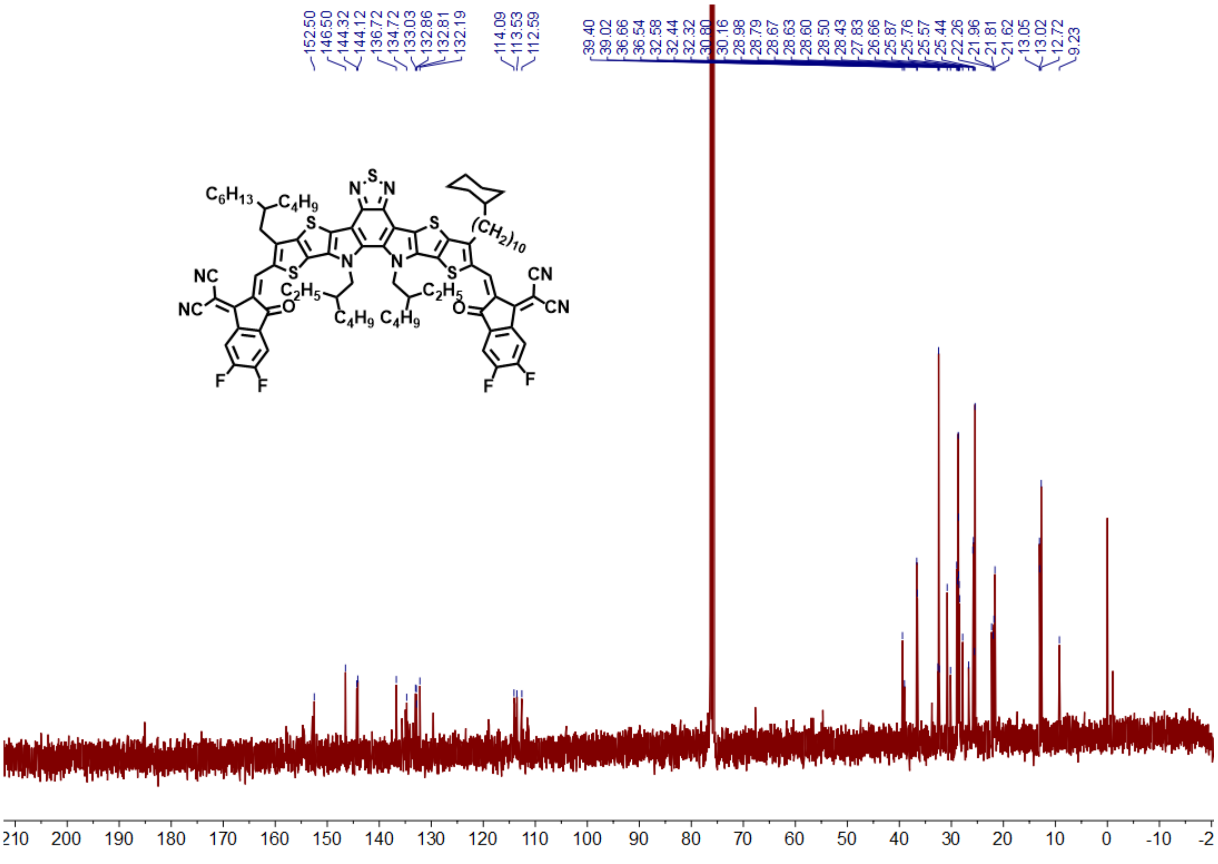

Figure S25. ^{13}C NMR spectrum of A-C10ch.

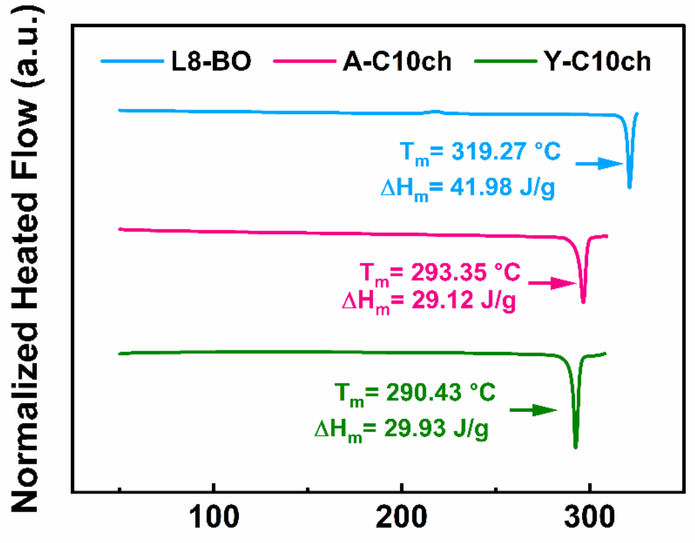

Figure S26. First heating DSC curves of L8-BO, A-C10ch and Y-C10ch under nitrogen atmosphere.

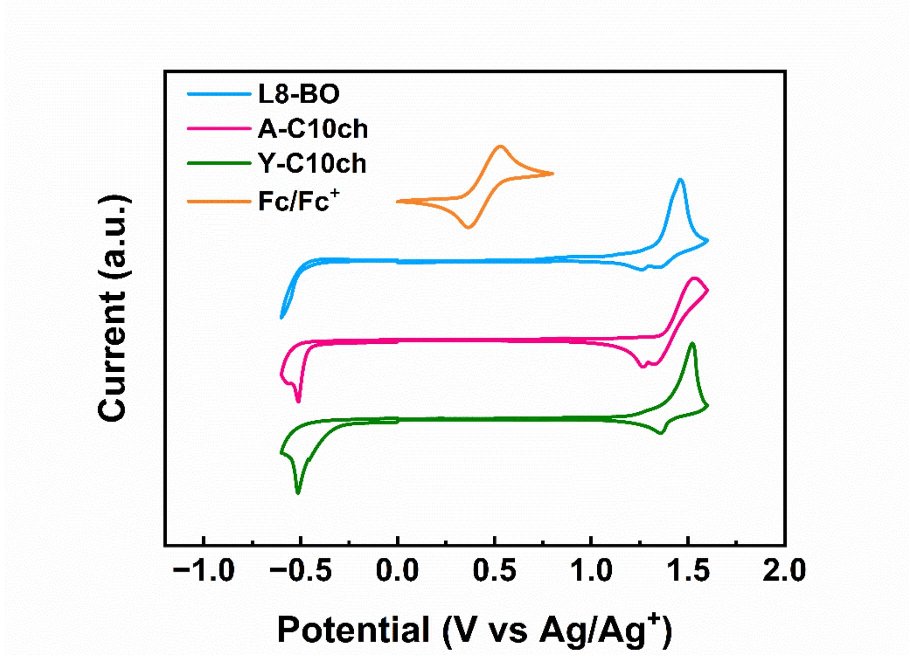

Figure S27. Electrochemical cyclic voltammogram curves of L8-BO, A-C10ch and Y-C10ch.

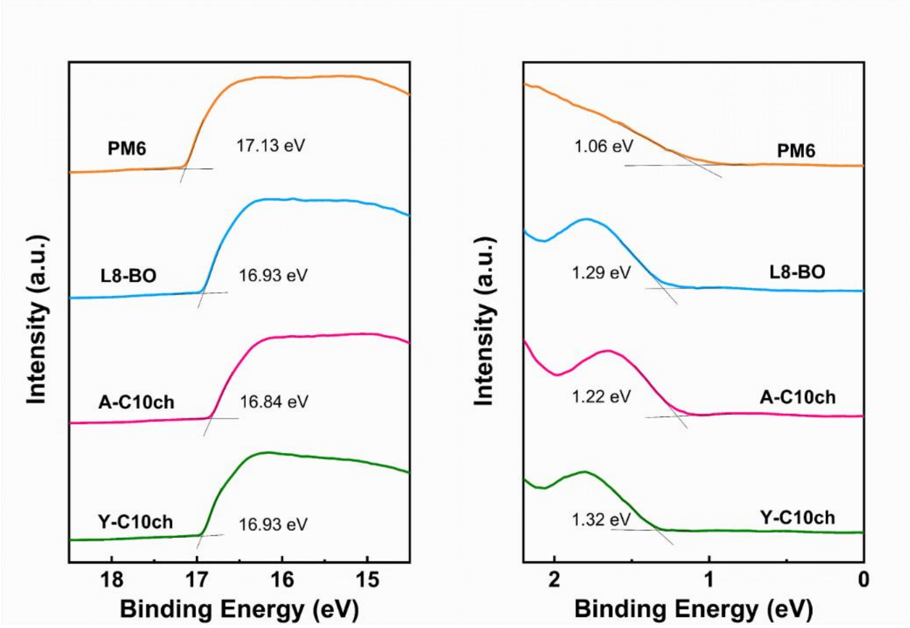

Figure S28. Cutoff and valence band regions of the UPS spectra of the donor and acceptors. Fermi energy was determined by linear extrapolation of the high binding energy portion of the spectrum, and HOMO energy level was referred to low binding energy onset.

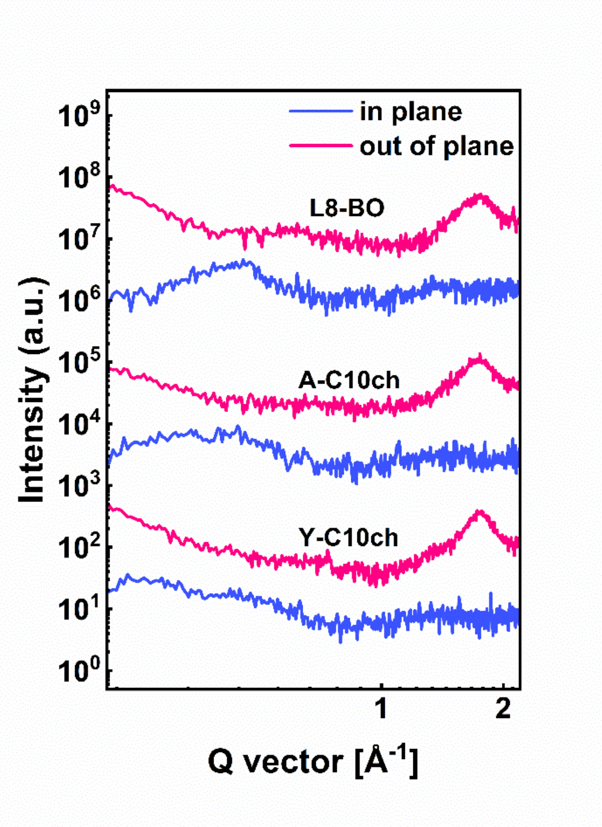

Figure S29. GIWAXS intensity profiles of the L8-BO, A-C10ch and Y-C10ch films along the IP and OOP directions.

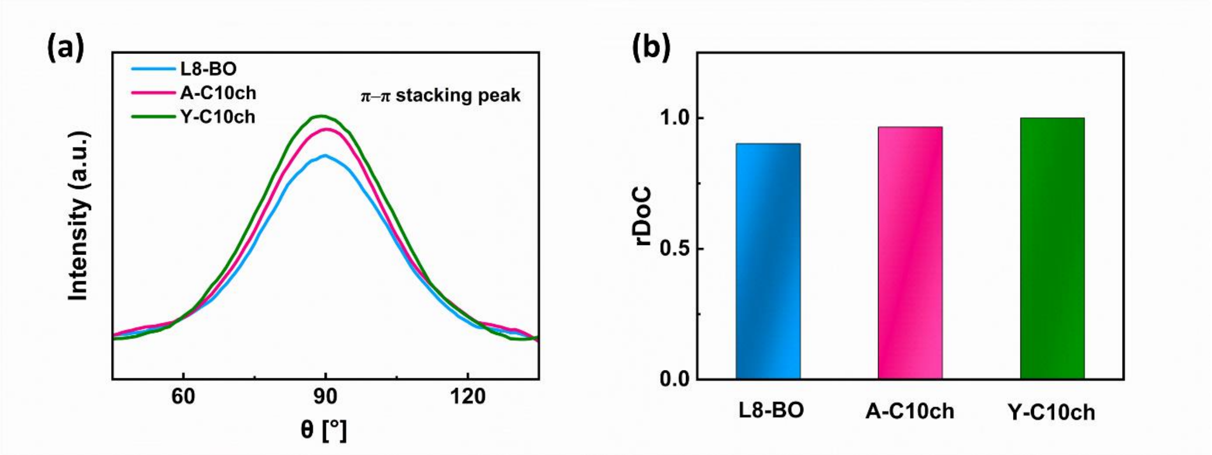

Figure S30. (a) Pole figures calculated from π–π stacking peak. (b) Relative degree of crystallinity of the three neat films obtained by integrating the pole figures.

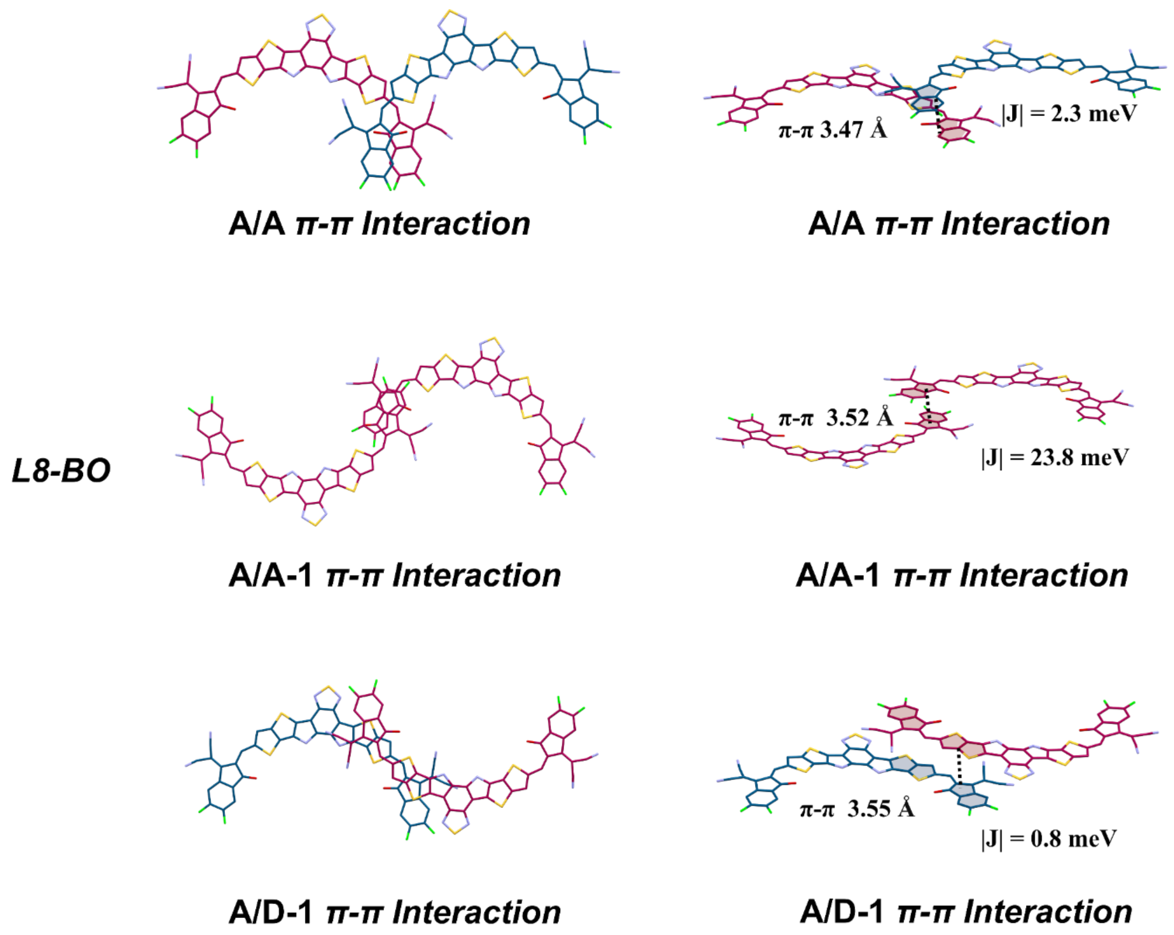

Figure S31. Dimer configurations of L8-BO.

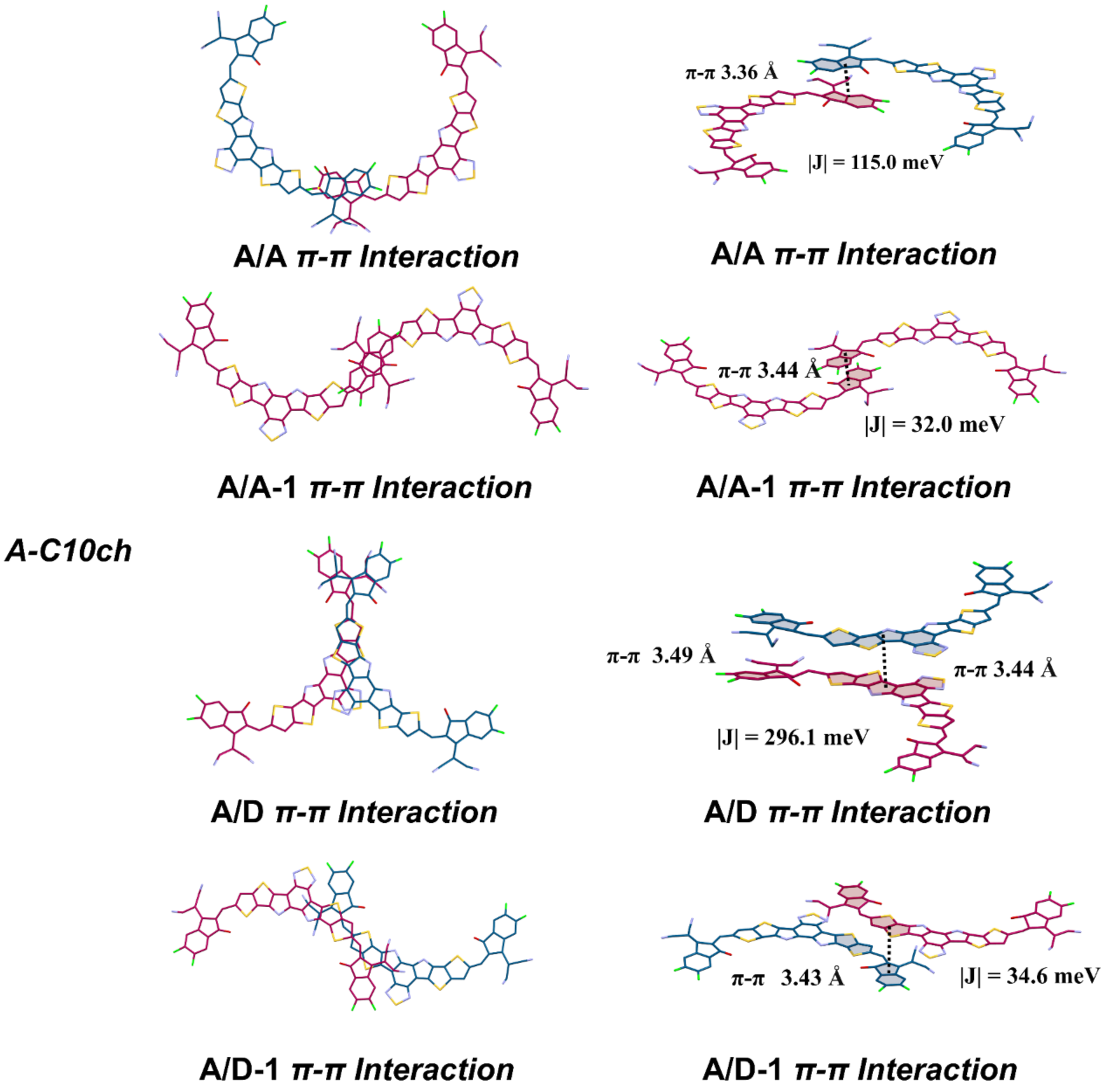

Figure S32. Dimer configurations of A-C10ch.

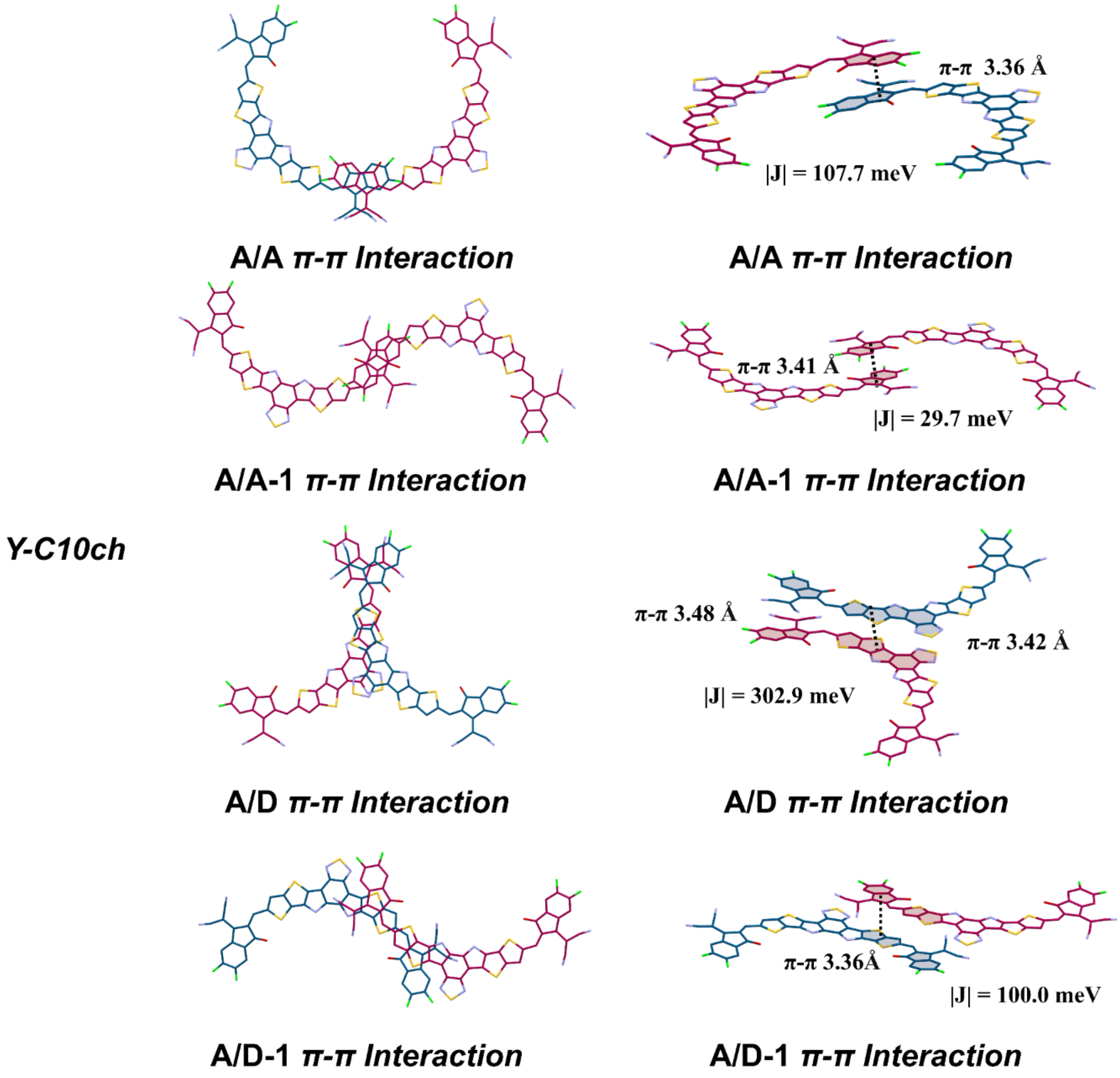

Figure S33. Dimer configurations of Y-C10ch.

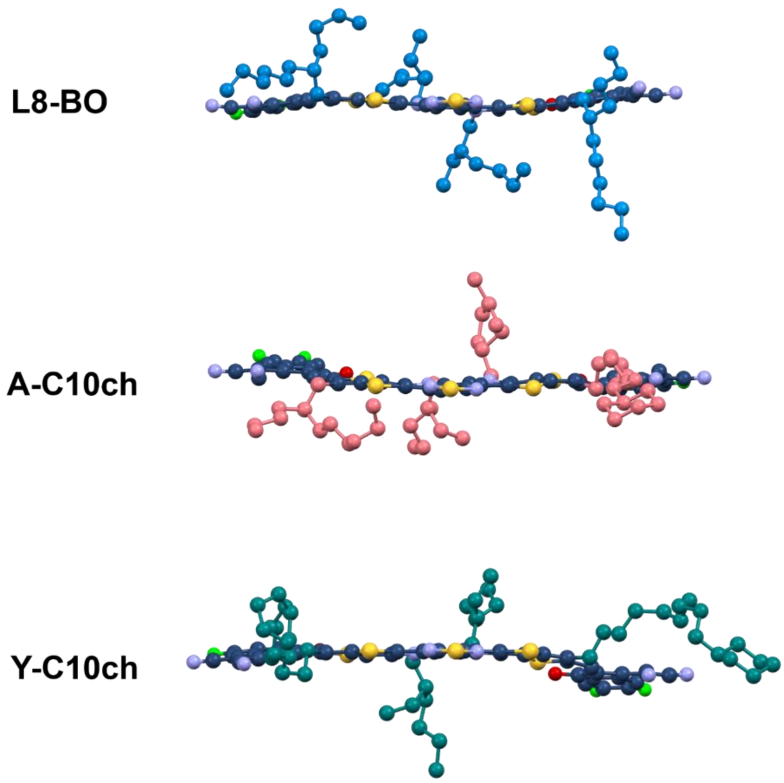

Figure S34. The side view of molecular conformations of L8-BO, A-C10ch and Y-C10ch single crystal.

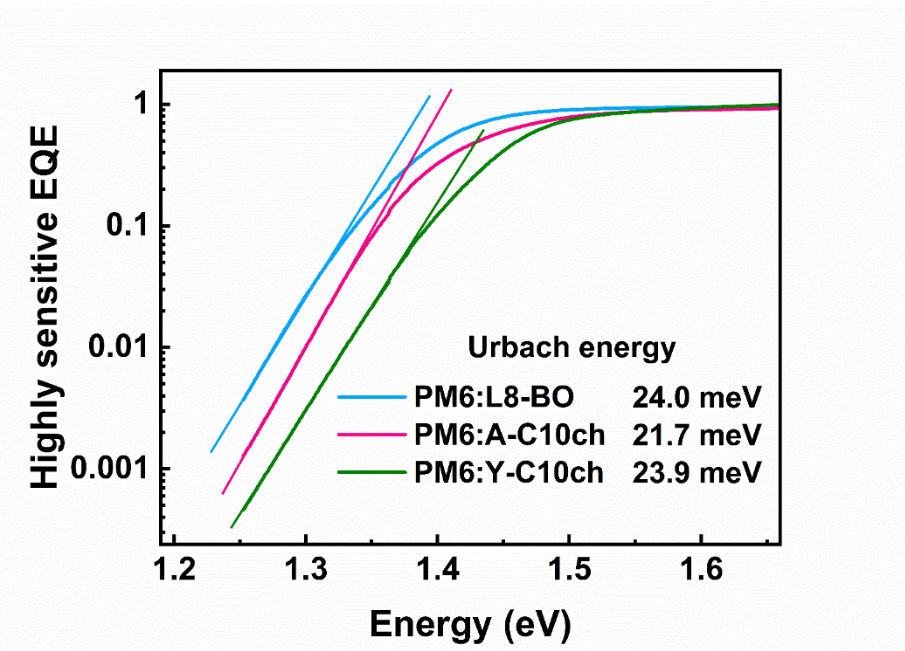

Figure S35. Highly sensitive EQE spectra of the devices.

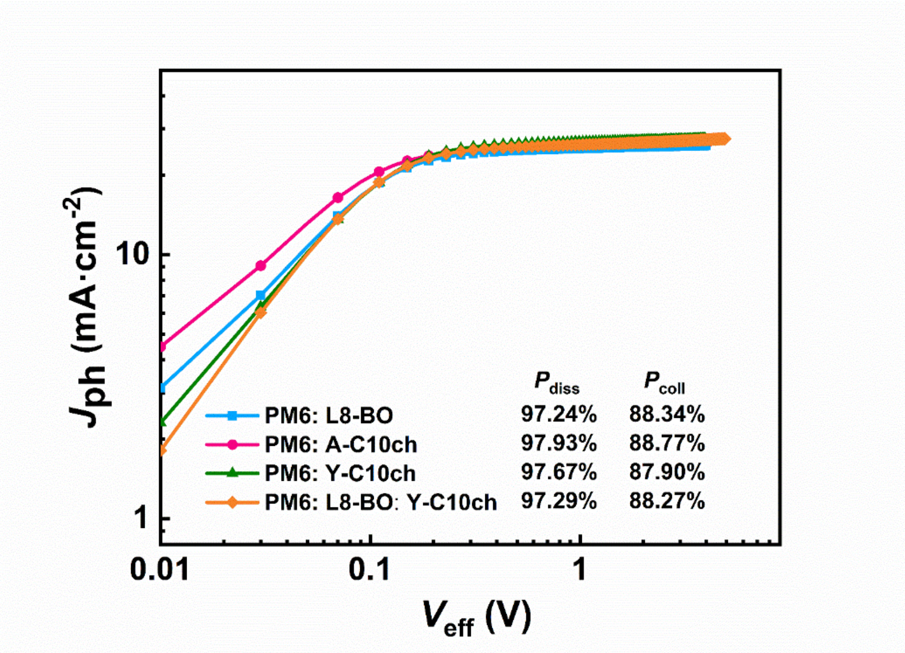

Figure S36. The photocurrent density (J_{ph}) versus effective voltage (V_{eff}) curves of the optimum OSC devices.

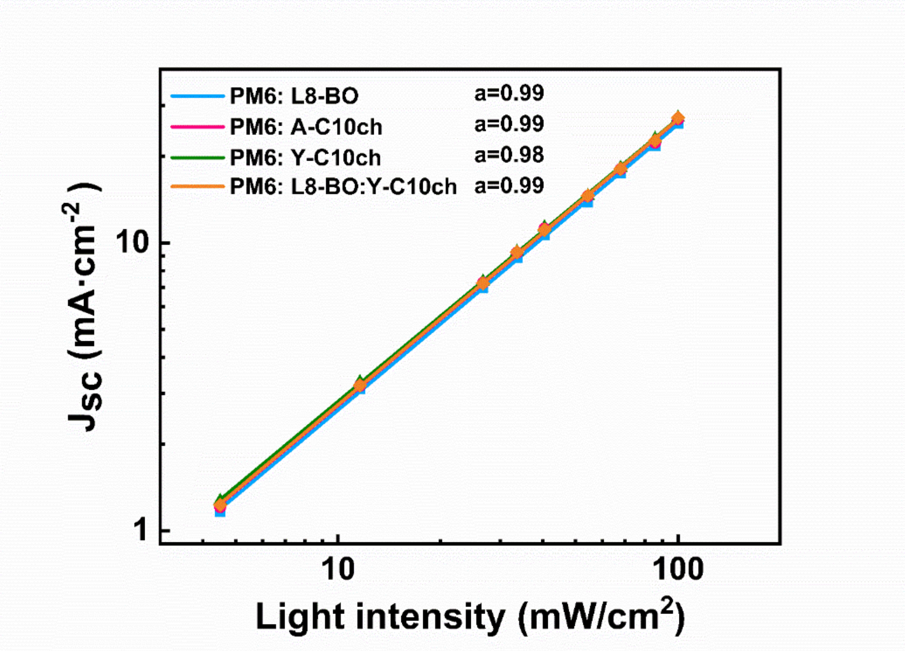

Figure S37. J_{sc} as a function of the light intensity of the solar cells based on L8-BO, A-C10ch, Y-C10ch and ternary OSCs device.

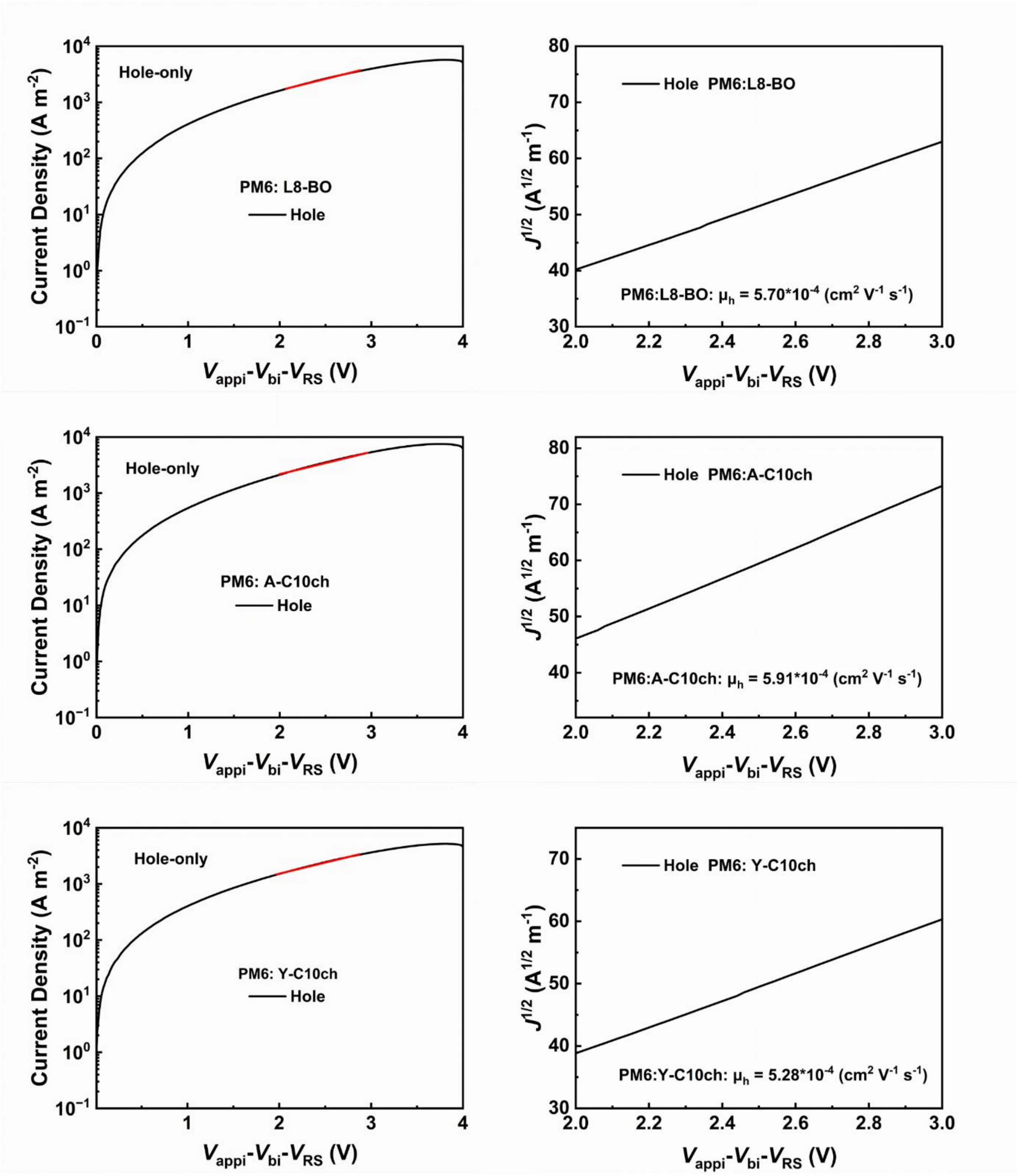

Figure S38. Current-voltage (J-V) characteristics of the optimized blend films in hole-only devices: ITO/PEDOT:PSS/blend film/MoO_3/Ag.

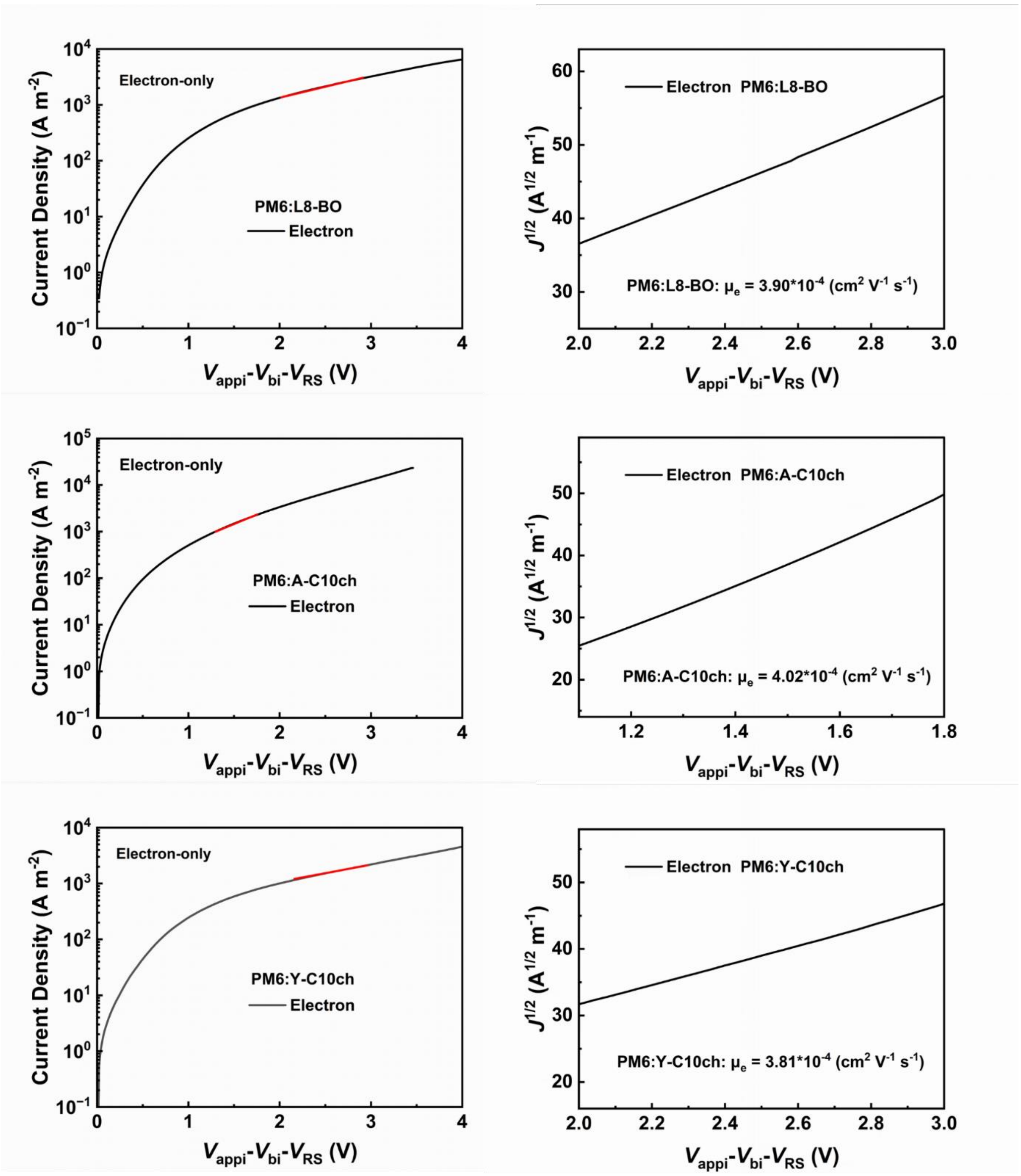

Figure S39. Current-voltage (J-V) characteristics of the optimized blend films in electron-only devices: ITO/ZnO/blend film/PNDIT-F3N/Ag.

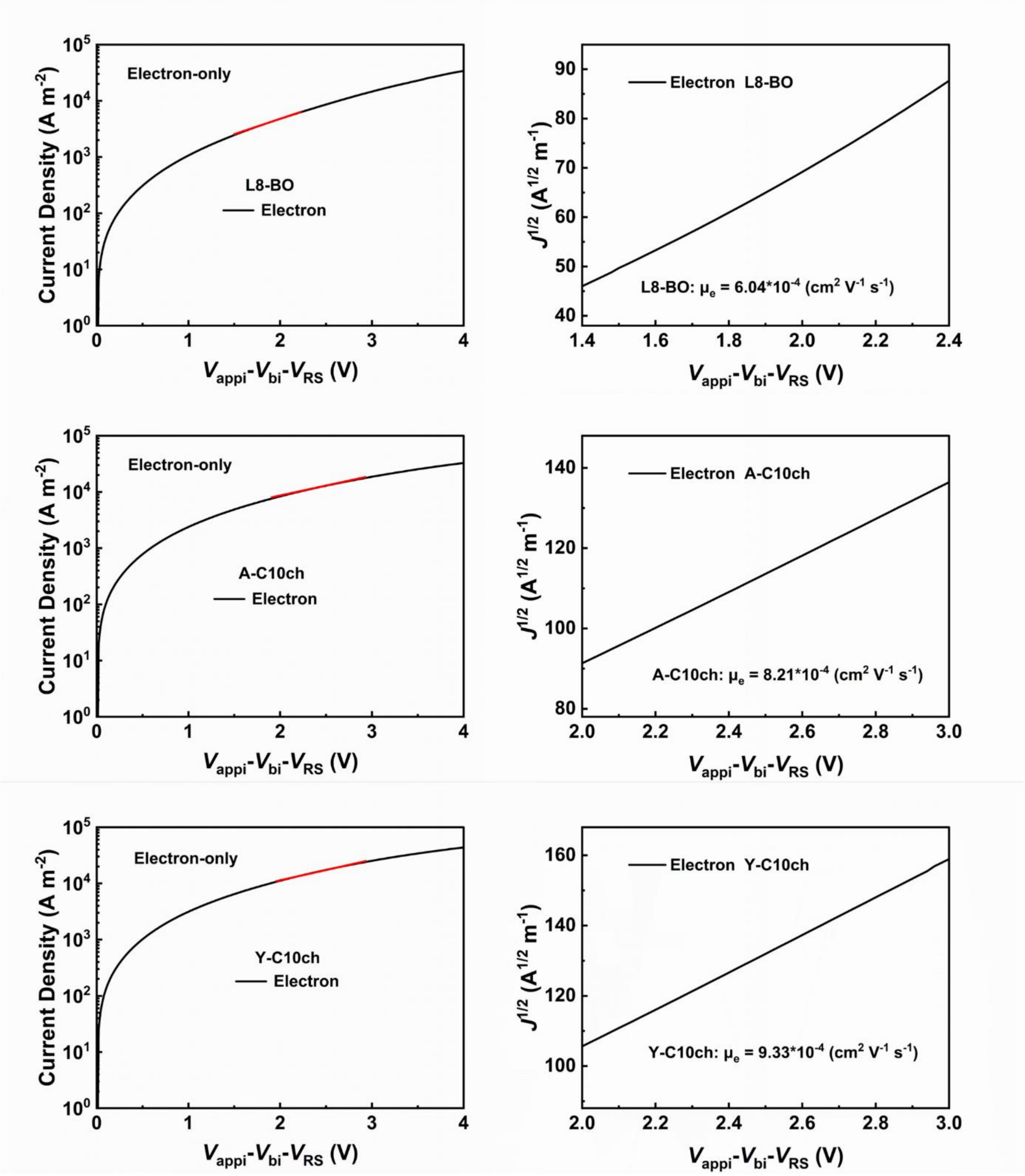

Figure S40. Current-voltage (J-V) characteristics of the pure neat films in electron-only devices: ITO/ZnO/acceptors/ PNDIT-F3N /Ag.

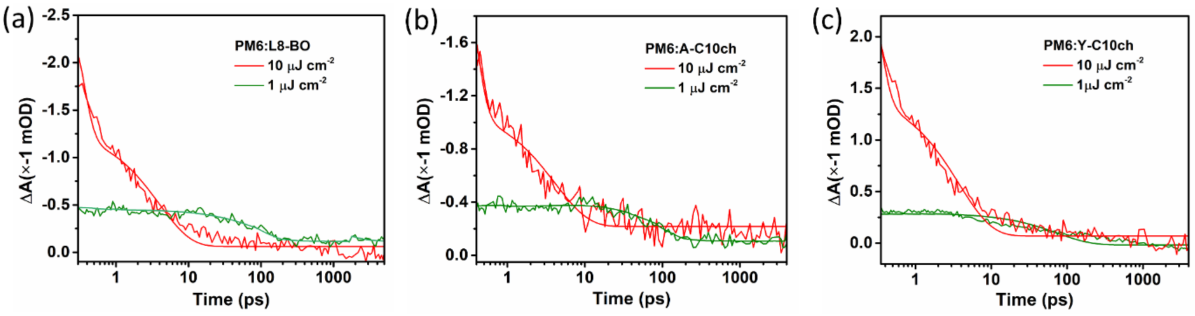

Figure S41. Singlet exciton decay dynamics of PM6:L8-Bo, PM6:A-C10ch and PM6:Y-C10ch probed at 820 nm (800 nm excitation) with different energy of pulse (1 μJ cm^{-2} and 10 μJ cm^{-2}).

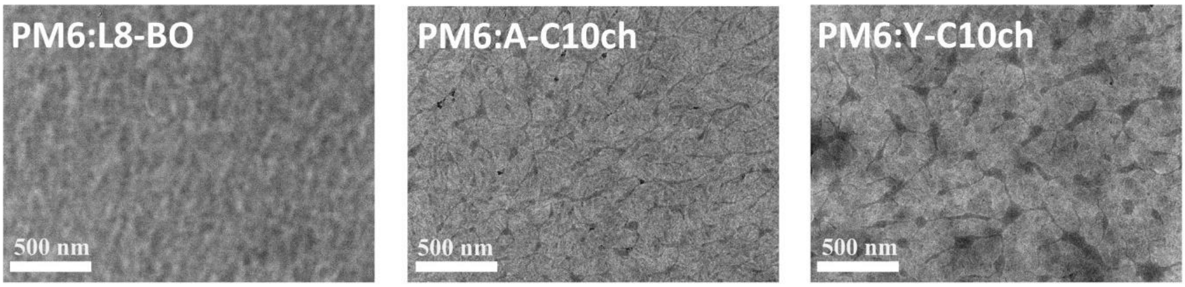

Figure S42. TEM images of the optimal blend films.

Sample	Out-of-plane			In-plane		
	q (Å^{-1})	d-spacing (Å)	CCL (Å)	q (Å^{-1})	d-spacing (Å)	CCL (Å)
L8-BO	1.72	3.65	25.4	0.43	14.6	74.1
A-C10ch	1.74	3.61	32.3	0.38	16.5	96.5
Y-C10ch	1.76	3.57	36.7	0.31	20.3	112.3

Table S1. Morphology data of the SMAs in the pristine films

Table S2. Morphology data of donor and acceptor in the blend films

Sample	Out-of-plane			In-plane		
	q (Å^{-1})	d-spacing (Å)	CCL (Å)	q (Å^{-1})	d-spacing (Å)	CCL (Å)
PM6: L8-BO	1.74	3.61	25.6	0.32	19.6	72.8
PM6: A-C10ch	1.76	3.57	25.2	0.30	20.9	102.4
PM6: Y-C10ch	1.77	3.55	27.4	0.28	22.4	123.7

Table S3. Crystal data and structure refinement for L8-BO, A-C10ch and Y-C10ch

Compound	L8-BO	A-C10ch	Y-C10ch
CCDC number	2005533		
Empirical formula	$C_{84}H_{90}F_4N_8O_2S_5$	$C_{88}H_{96}F_4N_8O_2S_5$	$C_{92}H_{102}F_4N_8O_2S_5$
Formula weight	1479.93	1534.08	1588.18
Temperature / K	150 K	193 K	193 K
Crystal system	monoclinic	monoclinic	monoclinic
Space group	C2/c	C c	C c
a/Å	27.704(3)	23.253(3)	23.7893(12)
b/Å	20.855(2)	58.467(6)	57.844(3)
c/Å	28.363(3)	14.3753(15)	14.4584(7)
α/°	90	90	90
β/°	105.949(10)	112.058(6)	118.056(2)
γ/°	90	90	90
Volume	15756(3)	18113(3)	17557.8(15)
Z	8	8	8
Dx, g cm^{-3}	1.248	1.128	1.205
μ (mm^{-1})	1.85	1.064	1.108
F(000)	6256	6524.0	6768.0
h, k, lmax	25,19,26	27,68,16	27,67,16
R(reflections)	0.1476	0.1773	0.1291
wR2(reflections)	0.3349	0.4456	0.3489
S	1.115	1.151	1.070

Table S4. Charge transport properties of L8-BO, A-C10ch and Y-C10ch neat films and the blend films

Active layer	Hole mobility (10^{-4} cm^2 V^{-1} s^{-1})	Electron mobility (10^{-4} cm^2 V^{-1} s^{-1})	μ_h/μ_e
L8-BO		6.04	
A-C10ch		8.21	
Y-C10ch		9.33	
PM6: L8-BO	5.70	3.90	1.46
PM6: A-C10ch	5.91	4.02	1.47
PM6: Y-C10ch	5.28	3.81	1.38

Table S5. Photovoltaic parameters of PM6:Y-C10ch OSCs under different optimized condition

D/A (w/w)	TA treatment (ºC)	1-CN (%)	V_{oc} (V)	J_{sc} (mA/cm^2)	FF (%)	PCE (%)
1:1.2	25	0.25	0.86	26.8	70.6	16.3
1:1.2	80	0.25	0.86	26.8	71.9	16.6
1:1.2	80	0.4	0.85	27.0	75.7	17.0
1:1.2	80	0.5	0.85	26.9	71.9	16.5
1:1.2	100	0.5	0.83	27.2	67.7	15.3

Table S6. Photovoltaic parameters of PM6:Y-C10ch OSCs under different optimized condition

D/A (w/w)	TA treatment (ºC)	DIO (%)	V_{oc} (V)	J_{sc} (mA/cm^2)	FF (%)	PCE (%)
1:1.2	25	0.25	0.85	27.0	73.4	16.9
1:1.2	80	0.25	0.84	27.1	74.0	16.9
1:1.2	80	0.4	0.83	27.2	76.4	17.3
1:1.2	100	0.25	0.84	27.3	74.7	17.2
1:1.2	100	0.4	0.83	27.5	74.5	17.1

Table S7. Photovoltaic parameters of PM6:Y-C10ch OSCs under different optimized condition

D/A (w/w)	TA treatment (ºC)	DIM (%)	V_{oc} (V)	J_{sc} (mA/cm^2)	FF (%)	PCE (%)
1:1.2	25	0	0.88	26.4	69.0	16.2
1:1.2	25	0.25	0.86	27.0	74.7	17.4
1:1.2	80	0.25	0.86	26.9	76.3	17.6
1:1	80	0.25	0.86	27.0	73.0	16.9
1:1.4	80	0.25	0.85	27.5	72.9	17.1
1:1.2	80	0.5	0.85	27.0	74.7	17.4
1:1.2	80	0.75	0.85	26.8	74.6	16.9
1:1.2	100	0.25	0.86	27.3	75.2	17.6

Table S8. Photovoltaic parameters of PM6:A-C10ch OSCs under different optimized condition

D/A (w/w)	TA treatment (ºC)	1-CN (%)	V_{oc} (V)	J_{sc} (mA/cm^2)	FF (%)	PCE (%)
1:1.2	25	0.25	0.88	26.30	75.5	17.5
1:1.2	80	0.25	0.88	26.55	77.5	18.2
1:1.2	80	0.4	0.86	27.03	78.2	18.1
1:1.2	80	0.5	0.86	26.54	76.6	17.5
1:1.2	100	0.5	0.85	26.10	75.1	16.6

Table S9. Photovoltaic parameters of PM6:A-C10ch OSCs under different optimized condition

D/A (w/w)	TA treatment (ºC)	DIO (%)	V_{oc} (V)	J_{sc} (mA/cm^2)	FF (%)	PCE (%)
1:1.2	25	0.25	0.88	26.3	75.4	17.5

1:1.2	80	0.25	0.88	25.9	76.1	17.4
1:1.2	80	0.4	0.86	26.9	76.9	17.9
1:1.2	100	0.25	0.87	26.2	76.8	17.5
1:1.2	100	0.4	0.86	25.8	70.67	15.9

Table S10. Photovoltaic parameters of PM6:A-C10ch OSCs under different optimized condition

D/A (w/w)	TA treatment (ºC)	DIM (%)	V_{oc} (V)	J_{sc} (mA/cm^2)	FF (%)	PCE (%)
1:1.2	25	0	0.90	26.0	64.5	15.2
1:1.2	25	0.25	0.89	26.4	77.9	18.2
1:1.2	80	0.25	0.89	26.6	78.1	18.4
1:1	80	0.25	0.89	26.1	75.5	17.4
1:1.4	80	0.25	0.88	26.7	77.0	18.1
1:1.2	80	0.5	0.88	26.7	76.4	18.1
1:1.2	80	0.75	0.88	26.6	76.4	18.0
1:1.2	100	0.25	0.87	26.6	77.2	17.8

Table S11. Photovoltaic parameters of PM6:L8-BO OSCs under different optimized condition

D/A (w/w)	TA treatment (ºC)	1-CN (%)	V_{oc} (V)	J_{sc} (mA/cm^2)	FF (%)	PCE (%)
1:1.2	80	0.25	0.89	24.5	75.7	16.4
1:1.2	80	0.4	0.88	24.4	75.8	16.3
1:1.2	80	0.5	0.87	25.5	73.4	16.3
1:1.2	100	0.5	0.87	25.7	75.3	16.9

Table S12. Photovoltaic parameters of PM6:L8-BO OSCs under different optimized condition

D/A	TA treatment	DIO (%)	V_{oc}	J_{sc}	FF	PCE

(w/w)	(ºC)		(V)	(mA/cm^2)	(%)	(%)
1:1.2	80	0.25	0.88	25.9	78.0	17.8
1:1.2	80	0.4	0.88	25.2	75.9	16.7
1:1.2	100	0.25	0.88	25.7	78.1	17.6
1:1.2	100	0.4	0.87	25.9	75.1	17.1

Table S13. Photovoltaic parameters of PM6:L8-BO OSCs under different optimized condition

D/A (w/w)	TA treatment (ºC)	DIM (%)	V_{oc} (V)	J_{sc} (mA/cm^2)	FF (%)	PCE (%)
1:1.2	80	0	0.91	24.9	73.2	16.6
1:1.2	80	0.25	0.90	25.6	78.8	18.1
1:1.2	80	0.5	0.90	25.2	78.0	17.7
1:1.2	100	0.25	0.90	25.6	77.6	17.8
1:1.2	100	0.5	0.89	25.2	77.9	17.4

Table S14. Photovoltaic parameters of PM6:L8-BO:Y-C10ch OSCs under different optimized condition

D/A (w/w)	TA treatment (ºC)	DIM (%)	V_{oc} (V)	J_{sc} (mA/cm^2)	FF (%)	PCE (%)
1:1.2(10wt%)	80	0.25	0.90	26.5	77.8	18.5
1:1.2(25wt%)	80	0.25	0.90	27.1	79.2	19.1
1:1.2(25wt%)	80	0.5	0.89	27.0	78.1	18.7
1:1.2(30wt%)	80	0.25	0.89	27.2	75.8	18.2
1:1.2(50wt%)	80	0.25	0.88	27.3	75.3	18.1

Table S15. Detailed parameters of single exciton decay dynamic for blend films

Materials	Pump energy	n_o (×10^{17} cm^{-3})	$t_{1/2}$ (ps)	k (×10^9 s^{-1})	a	γ (×10^{-9})	D (×10^{-3}	L_D (nm)

	(μJ cm^{-2})					cm^{-2} s^{-1})	cm^{-2} s^{-1})	
PM6:L8-BO	1	0.20	138	5.0				
	10	3.02	7.5		0.054	5.0	1.98	6.3
PM6:A-C10ch	1	0.17	109	6.3				
	10	1.89	8.2		0.074	6.0	2.39	6.2
PM6:Y-C10ch	1	0.14	95	7.3				
	10	2.20	6.4		0.066	6.8	2.70	6.1

Table S16. Fitting parameters of 1D GISAXS profiles of three cells

Blend film	ξ (Å)	D	η (Å)	Rg (nm)
PM6:L8-BO	282	2.99	53.3	11.2
PM6:A-C10ch	262	2.97	70.7	13.9
PM6:Y-C10ch	240	2.99	74.4	16.0

References

[1] H.-C. Liao, C.-S. Tsao, Y.-T. Shao, S.-Y. Chang, Y.-C. Huang, C.-M. Chuang, T.-H. Lin, C.-Y. Chen, C.-J. Su, U. S. Jeng, Y.-F. Chen, W.-F. Su, *Energy Environ. Sci.* **2013**, *6*, 1938.

[2] C. Li, J. Zhou, J. Song, J. Xu, H. Zhang, X. Zhang, J. Guo, L. Zhu, D. Wei, G. Han, J. Min, Y. Zhang, Z. Xie, Y. Yi, H. Yan, F. Gao, F. Liu, Y. Sun, *Nat. Energy* **2021**, *6*, 605.

[3] D. Qian, Z. Zheng, H. Yao, W. Tress, T. R. Hopper, S. Chen, S. Li, J. Liu, S. Chen, J. Zhang, X.-K. Liu, B. Gao, L. Ouyang, Y. Jin, G. Pozina, I. A. Buyanova, W. M. Chen, O. Inganäs, V. Coropceanu, J.-L. Bredas, H. Yan, J. Hou, F. Zhang, A. A. Bakulin, F. Gao, *Nat. Mater.* **2018**, *17*, 703.

[4] P. Bi, S. Zhang, Z. Chen, Y. Xu, Y. Cui, T. Zhang, J. Ren, J. Qin, L. Hong, X. Hao, J. Hou, *Joule* **2021**, *5*, 2408.
